# Proximity Labeling of the Tau Repeat Domain Enriches RNA-Binding Proteins That Are Altered in Alzheimer's Disease and Related Tauopathies

**DOI:** 10.1016/j.mcpro.2025.101458

**Published:** 2025-11-07

**Authors:** Sarah M. Shapley, Anantharaman Shantaraman, Joshna Gadhavi, Masin A. Kearney, Eric B. Dammer, Duc M. Duong, Christine A. Bowen, Caroline M. Watson, Pritha Bagchi, Qi Guo, Srikant Rangaraju, Nicholas T. Seyfried

**Affiliations:** 1 Center for Neurodegenerative Diseases, Emory School of Medicine, Atlanta, Georgia, USA; 2 Department of Biochemistry, Emory School of Medicine, Atlanta, Georgia, USA; 3 Emory Integrated Proteomics Core, Emory School of Medicine, Atlanta, Georgia, USA; 4 Department of Neurology, School of Medicine, Yale University, New Haven, Connecticut, USA

**Keywords:** Alzheimer’s disease, proteomics, proximity labeling, tau

## Abstract

In Alzheimer's disease (AD) and other tauopathies, tau dissociates from microtubules and forms toxic aggregates that contribute to neurodegeneration. Although some of the pathological interactions of tau have been identified from postmortem brain tissue, these studies are limited by their inability to capture transient interactions. To investigate the interactome of aggregate-prone fragments of tau, we applied an *in vitro* proximity labeling technique using split TurboID biotin ligase (sTurbo) fused with the tau microtubule repeat domain (TauRD), a core region implicated in tau aggregation. We characterized this sTurbo TauRD tagging interactors with the requirement for both ligase fragment co-expression for robust enzyme activity and nuclear and cytoplasmic localization of the recombinant proteins. Following enrichment of biotinylated proteins and mass spectrometry, we identified over 700 TauRD interactors. Gene ontology analysis of enriched TauRD interactors highlighted processes often dysregulated in tauopathies, including spliceosome complexes, RNA-binding proteins, and nuclear speckles. The disease relevance of these interactors was supported by integrating recombinant TauRD interactome data with human AD tau interactome datasets and protein co-expression networks from individuals with AD and related tauopathies. This revealed an overlap with the TauRD interactome and several modules enriched with RNA-binding proteins and increased in AD and progressive supranuclear palsy. These findings emphasize the importance of nuclear pathways in tau pathology, such as mRNA surveillance and RNA splicing, establishing the sTurbo TauRD system as a valuable tool for exploring the tau interactome.

## Highlights

• Proximity labeling revealed >700 proteins interacting with Tau repeat domains (TauRD).
• TauRD protein interactors show strong enrichment for nuclear RNA-splicing factors.
• Disease relevance was shown by linking TauRD interactors to human AD brain networks.
• Pathologic tau associates with nuclear RNA-binding proteins implicated in disease.

## Graphical Abstract

**Figure:**
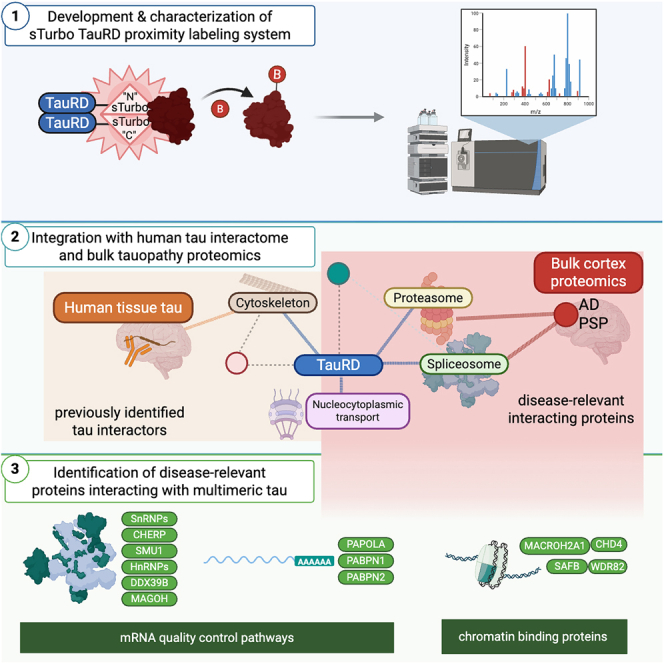

## In Brief

An *in vitro* proximity labeling technique using the conditional split TurboID biotin ligase identified over 700 proteins interacting with tau upon its oligomerization. The study highlighted tau's role in RNA processing, splicing, and nuclear transport pathways, validating these interactions by leveraging bulk human cortex proteomics from tauopathies. Overall, this study particularly emphasized potential co-aggregating RNA-binding proteins elevated in Alzheimer's disease and progressive supranuclear palsy, underscoring the association of nuclear dysfunction alongside pathological tau.

Tau protein, which is encoded by the microtubule-associated protein tau (*MAPT*) gene (1, 2), plays a crucial role in maintaining the dynamic nature of the cytoskeleton (3, 4). The misfolding and accumulation of Tau in the brain is the key characteristic of a group of disorders classified as Tauopathies (5, 6). In rare instances, mutations in the *MAPT* gene can lead to specific forms of frontotemporal dementia (7, 8). The aggregation of tau can also result in cognitive, behavioral, linguistic, and motor impairments, depending on the specific area of pathology. A significant feature of these conditions, including Alzheimer's disease (AD) and other tauopathies, is the formation of neurofibrillary tangles (NFTs) (9, 10), which are strongly correlated to the onset of cognitive decline (11).

Gain or loss of interaction partners is also an important consequence of disease pathophysiology and may result in “rewiring” of protein–protein interaction (PPI) networks, with associated alteration in function (12). Accordingly, in a pathological state, tau dissociates from microtubules and forms NFTs, leading to a loss and aberrant gain of tau PPIs. To identify tau interacting or co-aggregating partners, techniques such as affinity purification (AP) using tau antibodies (Abs), along with laser capture microdissection and mass spectrometry (MS), have been employed on postmortem brain tissue from AD cases and revealed numerous pathways and interactors associated with tau, highlighting its roles in translation, energy metabolism, long-term potentiation, and mitochondrial function (13, 14, 15, 16). Moreover, within large-scale proteomic analysis of human postmortem brain tissues, RNA-binding proteins (RBPs) have emerged as a class of proteins increased in abundance in AD compared to controls (CTLs) and enriched in modules that correlate with tau tangle pathology and splicing defects (17, 18, 19). To this end, several lines of evidence have demonstrated that tau interacts with RBPs under both normal and pathological conditions (20, 21). For example, tau immunoprecipitated (IP) from AD brain interacts with RBPs, including U1 snRNP proteins (U1-70K, U1A) (16). Many of these core RBPs, including U1-70K, mislocalize to the neuronal cytoplasm and co-aggregate with AD tau (22, 23). These findings have been confirmed through various MS methods and validated by immunohistochemistry in human AD brain (16, 24). Other RBPs found in tau aggregates include SRRM2 (25), PNN (16), TIA1 (26, 27, 28, 29), HNRNPA2B1 (30), Musashi 1/2 (31), SFPQ (32), and serine/arginine-rich splicing factors (33). Collectively, these findings imply that tau regulates RNA and RBP function and the sequestration of RBPs in tau aggregates. Although some of the pathological interactions of tau have been identified from postmortem brain tissue, these studies in tissue are limited by their ability to capture transient interactions.

To overcome these limitations, recent *in vitro* interactome studies have leveraged proximity labeling (PL) as a tool to dissect both transient and stable interacting partners of proteins of interest using a bioengineered promiscuous biotin ligase (14, 34, 35, 36). One such enzyme engineered from *E*.*coli*, TurboID, covalently binds biotin to lysine (Lys) residues of proteins within a 10 nm range in minutes. IP methods limit the use of ionic detergents, such as those used in insoluble protein preparations, for identifying aggregate-prone proteins in disease (37). In contrast, the use of biotin leverages its strong affinity to streptavidin for enrichment of these biotinylated interacting proteins in strong, denaturing lysis conditions. Overall, PL has proved to be a useful tool in characterizing protein interactors across cell signaling pathways, aberrant interactions in disease models, and protein trafficking (36, 38, 39, 40).

Here, we applied a PL technique using split TurboID, a modified form of TurboID that activates only when two domains are in close proximity (40, 41). We focused on the microtubule-binding repeat domain (MTBR) of tau, previously shown to form aggregate-like structures in mammalian cell culture upon overexpression (42, 43). Expression of two MTBR fragments carrying the N- and C-terminal of split TurboID (sTurbo) yielded robust biotinylation, confirmed by immunoblotting and immunocytochemistry (ICC). Using affinity capture MS, the sTurbo tau microtubule repeat domain (TauRD) system captured both known tau-interacting proteins and predominantly nuclear proteins, many of which are involved in RNA binding and had not been previously identified as tau interactors. Many of the sTurbo-interacting RBPs were also detected in post-mortem tissues and found to be enriched in the sarkosyl-insoluble fractions of AD brain tissues. Integrating these findings with independent tau interactome studies from human brain and proteomic co-expression analysis in AD and related tauopathies further highlighted a strong correlation between NFT burden and RBPs, many of which directly interact with tau in our sTurbo TauRD cell culture. Collectively, our study identified several novel tau-interacting proteins with relevance to human disease, providing promising candidates for mechanistic exploration.

## Experimental procedures

### Experimental Design and Statistical Rationale

#### Characterization of N- and C-sTurbo TauRD

Three biological replicates were collected for experiments where transfection conditions included: N-terminal split TurboID (N-sTurbo) TauRD, C-terminal split TurboID (C-sTurbo) TauRD, both N-and C-sTurbo TauRD, or reagent alone (mock). Each transfection condition also contained a biotin treatment and biotin negative group to determine background biotinylation (*i.e.,* endogenous biotinylated proteins).

#### In Vitro Mass Spectrometry Proteomics

Biological replicates represent different passage numbers of the same cell line, with conditions including transfected with sTurbo TauRD encoding plasmid and a mock group treated with reagent alone. HEK cells were prepared with four biological replicates in two experimental batches and a third batch consisting of two biological replicates, totaling 10 per transfection condition. Following quality control, six mock samples were analyzed for proteomics after streptavidin affinity purification (SA-AP). Thus, 16 cell lysates were included in the study. SH-SY5Y cells were prepared with four biological replicates for each transfection condition. All 16 samples were included for MS upon passing quality control. An *a priori* power analysis for sample size was not performed, although the highest sample size afforded was included. As previously detailed (35), mock peptides were run before sTurbo TauRD for MS to reduce column contamination between sample types in HEK runs. Details for further statistical methods are discussed in the “*Differential abundance and ontological enrichment*” and “*Human validation analyses*” subsection.

### Cloning and Plasmid Isolation

Five sTurbo TauRD plasmid constructs, N-sTurbo TauRD, C-sTurbo TauRD, and 2A sTurbo TauRD (P301L, WT, and KXGA), were created in coordination with Emory University's Integrated Genomics Core. The original N- and C-terminal sTurbo fragments correspond to Addgene #153002 and Addgene #153003, respectively, with the MTBR based on tau amino acids 243 to 375 (2N4R, 441 isoform). N-sTurbo TauRD includes the MTBR, a 25 amino acid SG-rich linker, a V5 tag, and N-terminal sTurbo ligase fragment and is driven by a human ubiquitin (hUb) promoter. C-sTurbo TauRD shares the MTBR and hUb promoter but features a 17 amino acid linker, an HA tag, and the larger C-terminal sTurbo fragment. Restriction enzyme sites were introduced with respective forward and reverse primers, outlined in Table S1. The pLEX-MCS UBC promoter was a kind gift from Oskar Laur (Addgene plasmid #128045 http://n2t.net/addgene:128045;RRID:Addgene_128045). The N- and C-sTurbo TauRD PCR products were then subcloned into a FUGW backbone with a puromycin-resistance ORF placed instead of GFP. The KXGA construct was generated by site-directed mutagenesis of residues S262, S293, S324, and S356 to encode for alanine residues. The 2A sTurbo TauRD (P301L, WT, and KXGA) PCR product was subcloned into a custom pLEX MCS vector. The 2A FRET TauRD plasmid [pLenti-UBC-TauRD(P301L)-CFP-2A-TauRD(P301L)-YFP plasmid] was a kind gift from Dr Derek Oakley. 2A plasmids (P301L, WT, KXGA, and FRET TauRD) were transformed into DH10B competent *E. coli* cells (Invitrogen, #18297010) according to manufacturer's instructions, while DH5-a *E. coli* was used for N- and C-sTurbo TauRD plasmids. Plasmid DNA was purified following the PureLink HiPure Plasmid Filter Maxiprep Kit instructions (Invitrogen, #K210016) and resulting concentration measured *via* Nanodrop.

### Cell Maintenance

HEK293T cells (ATCC and CRL3216) or SH-SY5Y cells (ATCC and CRL2266) were cultured in DMEM supplemented with 10% fetal bovine serum and 1% penicillin-streptomycin at 37 °C with 5% CO2, following standard protocol (35, 38, 44, 45). For passaging, Trypsin-EDTA 0.05% (Gibco, 25300054) was utilized to detach cells, which were subsequently resuspended and washed with 1× PBS. Cell counts were performed using a hemocytometer prior to experimental plating.

### DNA Transfection

Plasmid DNA was transfected into cells at 60 to 80% confluency using the jetPRIME Polyplus transfection reagent (VWR, #89129) based on the manufacturer's protocol. For 24-well dishes and 8-well chamber slides (Nunc Lab-Tek II Chamber Slide System, Thermo Scientific #154453), transfection was performed with 50 μl jetPRIME buffer, 1 μl jetPRIME reagent, and 0.5 μg of plasmid DNA per transfection in 500 μl of media. For cotransfection, equal amounts of N- and C-sTurbo TauRD plasmids were combined to reach the total recommended concentration. Mock transfections included the same ratio of reagents without plasmid. Cells were harvested or fixed 48 h post-transfection.

### Biotin Treatment

On the same day as cell collection or fixation, biotin labeling was performed following standard procedure, with minimal modification (38). Biotin (Sigma, B4639) was prepared as a 10 mM stock solution in 1× PBS (pH 9.0) and stored at 4 °C. A fresh 200 μM biotin solution was made by diluting the stock in serum-free (SF) media. After aspirating the complete media, cells underwent a biotin wash-out period in SF media for approximately 3 h. Subsequently, biotin positive groups were incubated in the 200 μM biotin supplemented media for 1 h before continuing to respective collection or fixation conditions.

### Cellular Lysis

Cells were lysed according to standard laboratory protocol (38, 44). Briefly, cells were collected in fresh lysis buffer (8 M urea, 10 mM Tris, and 100 mM NaH2PO4, pH 8.5) for proteomic assays with 1× HALT protease/phosphatase inhibitor (Thermo Sci, #78446). Lysates were sonicated at 30% amplitude for three 5-s intervals, with the sonication tip cleaned between samples. Following centrifugation at 18,000*g* for 15 min at 4 °C, supernatants were collected. Protein concentrations were determined using the Bicinchoninic acid assay (Pierce) and stored at −20 °C.

### Subcellular Fractionation

Cell pellets were collected according to established protocols (46). Briefly, cells were rinsed three times with room temperature 1× PBS, the last wash containing 1× HALT protease, and phosphatase inhibitors. PBS was aspirated, then cells were scraped into Lo-Bind Eppendorf microcentrifuge tubes and gently pelleted by centrifugation at 400*g* for 5 min at 4 °C. Remaining PBS was aspirated, and then the cell pellet was flash frozen on dry ice and stored at −80 °C until subsequent fractionation. Pellets were thawed on wet ice and then washed once with ice-cold 1× PBS. Subcellular fractionation was carried out in accordance with standard procedure (44). Briefly, cell pellets were centrifuged for 1000*g* for 5 min at 4 °C and then washed once with 1× PBS. 10% of resuspended whole cell slurry was collected as the total fraction. Remaining cells were centrifuged again, supernatant aspirated, then incubated for 5 min on ice in hypotonic lysis buffer (10 mM HEPES pH 7.9, 20 mM KCl, 0.1 mM EDTA, 1 mM dithiothreitol (DTT), 5% glycerol, 0.5 mM PMSF, 10 μg/ml aprotinin, 10 μg/ml leupeptin, 0.1% NP-40, 1× HALT protease, and phosphatase inhibitor). The cytoplasmic (C) fraction was collected upon centrifugation of lysates for 10 min at 15,000*g* at 4 °C. The nuclear fraction was collected after a 30 min incubation of the resulting pellet in high salt buffer (20 mM HEPES pH 7.9, 0.4 M NaCl, 1 mM EDTA, 1 mM EGTA, 1 mM DTT, 0.5 mM PMSF, 10 μg/ml aprotinin, 10 μg/ml leupeptin, 1× HALT protease, and phosphatase inhibitor). All lysates were sonicated for 5s at 25% and centrifuged at 18,213*g* for 10 min at 4 °C. The nuclear supernatant was retained as the nucleoplasm fraction, while the chromatin pellet was resuspended and sonicated in nuclei lysis buffer (50 mM Tris-HCl pH 8.0, 10 mM EDTA, and 1% SDS). Only cytoplasm and nucleoplasm fractions were used for Western blot (WB) analyses.

### SDS-PAGE, WB, and Semiquantification Analysis

Lysate total protein amount was normalized and diluted to 1× Laemmli sample buffer (Bio-Rad) and 355 mM β-mercaptoethanol (Sigma-Aldrich) as per Table S2. SDS-PAGE and WB were run as previously described (35, 44, 47). Samples were boiled and loaded onto 1.0 mm 4 to 12% Bis-Tris NuPage gels (NW04122, Invitrogen) alongside a molecular weight marker (New England Biosciences, P7719S, or ThermoFisher, #26616). Electrophoresis was carried out in 1× MES running buffer until the molecular weight marker reached the end of the gel. Proteins were then transferred to a 0.2 μm nitrocellulose membrane at 20 V for 7 min using the iBlot dry transfer stack system (Invitrogen) and blocked for 30 min in StartingBlock buffer (Thermo Scientific, #37543). Abs were diluted in StartingBlock buffer and incubated per Table S2. Membranes were washed twice with TBS-T, followed by TBS washes, before imaging on the Licor Odyssey M system. Validation APs and WBs were conducted essentially as described with minor modifications. Samples with minimal nonspecific binding shown by silver staining were selected for validation blots.

#### WB and Silver Stain Intensity Semi-quantification

Protein band intensities were quantified using ImageJ FIJI software (v2.14.0, National Institutes of Health), essentially as previously described (47).

#### WB N- and C-sTurbo TauRD Signals From 2A Construct

Biotinylated N- and C-sTurbo TauRD fragments, overlapping with V5 and HA signals, respectively, were selected as ROIs. Intensity measurements were selected from the Streptavidin 680 blot to reduce bias from Ab signals or channel intensity variation. Inverted intensities were analyzed in GraphPad Prism (v10.1.1), and the percentages of each streptavidin C-sTurbo and N- sTurbo value by the sample total were visualized as stacked bar plots.

### ICC and Microscopy

Acid-etched sterile coverslips (Bellco Glass SKU: 1943-10012A) were coated with Poly-L-Lysine (Sigma P1524, MW > 300,000) according to manufacturer's instructions. Cells were transfected with experimental plasmids, then underwent biotin labeling protocol the same day as fixation with 4% paraformaldehyde. Cells were prepared for immunofluorescence as previously reported (38). All Abs were diluted in Ab buffer (1× PBS in 2% normal horse serum). Abs for ICC were as follows: 1:500 rabbit anti-V5 (abcam: 206566), 1:500 mouse tau 2E9 (Novus Biologicals, NBP2-25162), incubated for 1 h at room temperature, and 1:300 rabbit anti-HA tag Ab (abcam: ab9110) incubated overnight at 4 °C. Secondary Abs were as follows: 1:500 Donkey anti-Rabbit FITC conjugated (Invitrogen, A16030), 1:500 Streptavidin DyLight 594 (Invitrogen, #21842), and 1:500 Donkey anti-Mouse FITC conjugated (Invitrogen, A24507) and incubated at room temperature for 90 min in the dark. Primary and secondary only controls were incorporated to ensure specificity of fluorescence signal. All wells were counterstained with 1 μg 4′,6-diamidino-2-phenylindole, DAPI (Molecular Probes, D3571) in the first 5-min 1× PBS wash in the dark for 5 minutes to stain for nuclear (N) compartment. Cells were imaged with a 40× objective on a Keyence BZX-800 wide-field microscope.

### Streptavidin Affinity Purification

SA-AP was conducted according to standard protocol (34, 35, 38) Briefly, for HEK cell lysates, 1 mg of protein was normalized to 500 μl with cold RIPA buffer (pH 7.5, composed of 50 mM Tris-HCl, 150 mM NaCl, 0.1% SDS, 0.5% sodium deoxycholate, and 1% Triton X-100) and then underwent a 1 h incubation at 4 °C on a rotator with 83 μl prewashed streptavidin-conjugated magnetic beads (ThermoFisher, 88817). SH-SY5Y cell lysates were scaled to 500 μg input lysate to 41.5 μl streptavidin beads. Sequential 1 ml washes were performed to reduce nonspecific protein binding to magnetic beads: RIPA for 8 min, 1M KCl for 8 min, fresh 0.1M Na2CO3 for 10s, 2M Urea in 10 mM Tris-HCl (pH 8.0) for 10s, and then two 8-min RIPA washes. Two more 1× PBS washes were performed, after which the beads were resuspended in the original volume. Approximately, 10% of the beads were collected, PBS removed, and then resuspended in 30 μl of Laemmli sample buffer containing 2 mM biotin and 20 mM DTT. Proteins were boiled from the beads at 95 °C for 15 min. Ten microliter of this aliquot was retained for WB analysis, while 20 μl was allocated for silver stain. The remaining beads were prepared for protease digestion and MS.

### Silver Staining Procedures and Semiquantification

Silver staining was conducted before MS and WB validation to assess the effectiveness of the SA-AP. The silver stain protocol followed the manufacturer's instructions (Pierce, 24,612). Following fixation, washing, and staining steps, the gel was incubated in developer solution until protein ladder turned brown and protein bands were visualized the quickly rinsed with silver stain stop solution (5% acetic acid) and then incubated in fresh stop solution before imaging on the Odyssey M with the RGB Trans channel.

#### Silver Stain Quality Control Gel

FIJI intensities were obtained from the entire lane as ROI. These values were assessed in GraphPad Prism and visualized in a scatter dot plot for mock and sTurbo TauRD groups. Mock intensities above background levels were excluded from further analysis.

### Protease Digestion

Mock transfection and sTurbo TauRD SA-AP samples were digested essentially as described (34, 35, 38). Briefly, samples were normalized to 300 μl in 100 mM Tris-HCl pH 8 buffer. Lysates were reduced with 1 mM DTT and alkylated with 5 mM iodoacetamide at room temperature for 30 min, respectively. Proteins were digested overnight at room temperature with 2 μg Lys-C protease (Wako). The next day, 2 μg trypsin was added and incubated overnight. The following day, enzyme activity was quenched with acidifying buffer (1:9, v/v; 10% formic acid and 1% trifluoroacetic acid) and desalted by loading the peptides onto a 10 mg Oasis PRiME HLB 96-well plate (Waters), followed by washing twice with buffer A (0.1% TFA) and then eluted with buffer C (50% acetonitrile, ACN, and 0.1% TFA). Desalted peptides were lyophilized with a CentriVap Centrifugal Vacuum Concentrator (Labconco) overnight.

### Liquid Chromatography–Tandem Mass Spectrometry

#### Data-Dependent Acquisition

Peptides were resuspended and sonicated in Buffer A for loading. Twenty microliter was loaded onto an Evosep One tip, then separated by an Evosep One Liquid Chromatography (LC) system using a 15 cm × 150 μm i.d. Water's 1.7 μm CSH capillary with a preprogrammed 88 min gradient procedure (15 samples per day). MS procedure was executed essentially as described (34, 35). MS was performed with an Orbitrap Fusion Lumos Tribrid (ThermoFisher) in positive ion mode for data-dependent acquisition (DDA) MS. The mass spectrometer was run at top speed mode with each cycle lasting for 3 s and consisted of one full MS scan and as many MS/MS events as possible within the allowed cycle time. MS scans were collected at a resolution of 60,000, with a 400 to 1600 m/z range, 4 × 10^5^ automatic gain control (AGC), and a maximum ion injection time of 118 ms. All higher energy collision-induced dissociation MS/MS spectra were acquired at a resolution of 30,000, with a 1.6 m/z isolation width, 30% collision energy, 5 × 10^4^ AGC target, and 54 ms maximum ion injection time. Previously sequenced peaks were dynamically excluded for 30 s within a 10-ppm isolation window.

#### Data-Independent Acquisition

Each sample was loaded onto Evotips and analyzed by LC coupled to tandem MS. Peptide eluents were separated on IonOptick's Aurora Elite column (15 cm × 75 μM internal diameter (ID) packed with 1.7um resin) by a Evosep One (Evosep). Buffer A was water with 0.1% (v/v) formic acid, and Buffer B was 100% (v/v) ACN in water with 0.1% (v/v) formic acid. Elution was performed using the preset 40 samples per day Whisper Zoom method. Peptides were monitored on a Orbitrap Astral mass spectrometer (ThermoFisher Scientific) fitted with a high-field asymmetric waveform ion mobility spectrometry (FAIMS Pro) ion mobility source (ThermoFisher Scientific). One compensation voltage of −40 was chosen for the FAIMS. Each cycle consisted of one full scan (MS1) performed with an m/z range of 380 to 980 at 240,000 resolution, 500% AGC, and 3 ms injection time. The higher energy collision-induced dissociation data-independent acquisition (DIA) scans were collected with a 3 m/z isolation windows over the entire precursor range (380-980 m/z) with a time of 0.6 s and 3 ms injection time. Collision energy was set to 27%, and scan range set to 150 to 2000 m/z with a total of 199 scan events. Instrument mass list provided in Table S3.

### Database Search and Quantification

DDA raw files were searched with FragPipe (v 21.1). FragPipe relies on its integration with MSFragger (v 4.0) for peptide identification from a closed database. The proteins were identified by searching against the February 2024 canonical Uniprot database with 20,597 proteins and four independent protein sequences aligning to the N- sTurbo, C-sTurbo, V5 tag, and HA tag ORFs to identify the sTurbo TauRD recombinant protein. Fifty-one total contaminant sequences and all 20,652 reverse sequences were added for false discovery rate (FDR) calculation, which was set to 1%. For protein identification used for statistical analyses, the default FragPipe Label-Free Quantitation–Match Between Runs workflow was loaded with default parameters with minimal modifications.

Percolator (v3.6.4) filtered peptide spectral matches (PSMs) using a support vector machine algorithm to control PSMs matched to peptides from decoy proteins and best discern true PSMs to be kept in final protein assembly. Peptide-to-spectra matches were rescored with MSBooster and Percolator for predicting retention time and spectra with a minimum probability of 0.5. Briefly, precursor mass tolerance was −20 to 20 ppm, fragment mass tolerance of 20 ppm, with mass calibration and parameter optimization selected, and isotope error was set to 0/1/2. Enzyme specificity was set to strict-trypsin with up to four missed cleavages allowed due to the potential Lys-biotin modification impacting tryptic cleavage. Peptide length ranged from 7 to 50 amino acids and peptide mass from 500 to 5000 Da. Variable modifications included methionine oxidation (+15.9949 Da), N-terminal acetylation (+42.0106 Da), and phosphorylation on STY residues (+79.96633 Da) with a maximum of three variable modifications per peptide. Fixed modification included cysteine carbamidomethylation (+57.02146 Da).

Quantification was conducted by the IonQuant module (v1.10.12) with MBR permitted. Abundances were also reported using with the topN method with topN peaks set to 200. IonQuant parameters were as follows: two minimum ions, three minimum scans, minimum matched fragments of 4, and a retention time tolerance of 0.4 min. After FDR filtering with Philosopher (v 5.1.0), a total of 60,847 PSMs, 54,148 modified peptides, 50,674 peptides, and 5262 protein groups were detected. HEK sample protein coverage (%) and peptide-level information are included in Table S4. Peptides identified by 1 spectral hit were annotated with ProtViz by adapted from https://github.com/edammer/MQ1pepAnnotate for FragPipe file outputs. Annotated spectra are provided as supplemental. The original DDA search was modified to capture biotinylated peptides with parameters as follows: modified Label-Free Quantitation–Match Between Runs workflow with enzyme specificity set to semi-tryptic with up to four missed cleavages. Peptide length ranged from 7 to 60 amino acids. Variable modifications included methionine oxidation, N-terminal acetylation, with Lys (K) and peptide terminus biotinylation (+226.0776). Peptides with more than two spectral matches (n = 50) across samples were explicitly annotated.

DIA raw files were searched with DIA by neural networks (DIA-NN) software (v 2.2.0) upon generating an in silico spectral library generated with the previously used FASTA, including unique inputs for P301L and KXGA sequences. The following parameters were applied, following DIA-NN documentation recommendations (https://github.com/vdemichev/DiaNN): peptide length between 7 and 30, precursor range 300 to 1800 m/z and charges of 1 to 4, fragment range of 200 to 1800 m/z, tryptic cleavage at K and R residues with up to two missed cleavages, up to two variable modifications allowed, unimod 4 (cysteine carbamidomethylation, +57.02146 Da) included as a fixed modification, N-terminal methionine excision enabled, mass accuracy of 10.0, mass accuracy MS1 of 4.0, peptidoform scoring mode, normalization disabled, and FDR output filtered to 1%. The resulting library contained 20,706 proteins from 20,469 unique gene groups and 6,901,065 precursors. The library was reannotated and contained common contaminants. DIA scan events and resulting DIA-NN.parquet output are included as Tables S3 and S5, respectively.

### Differential Abundance and Ontological Enrichment

After filtering out proteins absent in 50% or more across HEK samples (17), 2167 proteins were retained for downstream analyses. An in-house function performed sample-wise imputation of missing values similar to the Perseus program (48) in R (v 4.3.3, 2024-02-29). Briefly, values were imputed according to a normal distribution falling between ±0.3 standard deviations from the noise level. The noise level was assumed to be −1.8 standard deviations from the mean of the Log2(LFQ intensity) values. The resulting abundance matrix was used for differential abundance and bioinformatics analyses. Raw intensity values are provided for pre- and post-missingness thresholding (Tables S6 and S7). A Student's two-tailed *t* test was implemented to determine differentially enriched proteins across groups (Table S8). After filtering out proteins absent in 50% or more across each SH-SY5Y condition, 4083 proteins were retained for downstream analyses. An in-house Perseus-style imputation of missing values was implemented (Table S9). Bootstrap regression of replicates was performed to remove batch variation (Table S10) (49, 50). The resulting abundance matrix was used for differential abundance of sTurbo TauRD groups comparison to mock samples. To compare between sTurbo TauRD conditions, the average intensity value of N and C sTurbo protein intensities was used for normalization (Table S11).

Volcano plots of -Log10 unadjusted *p*-values were generated using the custom plotVolc function, accessible *via* https://www.github.com/edammer/parANOVA. This script also provides the parANOVA.dex function for fast parallel calculation of one-way ANOVA with Tukey-corrected pairwise comparison *p* values when there are more than two groups compared. Bonferroni correction was employed instead of Tukey post hoc test in cases where Tukey *p* value estimates were less than 1 × 10^−8.5^, avoiding a ceiling effect due to imprecise small Tukey *p* value estimation.

Ontologies enriched by module were determined by Fisher's exact test (FET) of enriched sTurbo TauRD proteins using the GOparallel function. This script calculates one-tailed enrichment *p* value for input lists into over 13,000 human Gene ontology (GO) annotated gene lists from a GMT-formatted file obtained from the Bader Lab site December 1st 2023, as previously described (47, 51, 52, 53, 54, 55, 56, 57, 58, 59). Supporting online resources are available *via* https://www.github.com/edammer/GOparallel.

### Human Brain Tissue Proteomics

#### University of Pennsylvania (UPenn) Protein Quantification and Subset by Disease Condition

The University of Pennsylvania cohort raw files and metadata were obtained from synapse.org syn53177242. Brain tissue from the dorsolateral prefrontal cortex (BA 9) preparation, isobaric tandem mass tag (TMT) labeling, subsequent MS data acquisition, and protein quantification is originally detailed in Shantaraman *et al*, 2024 (60). All 364 UPenn samples underwent technical artifact batch normalization using a Tunable Approach for Median Polish of Ratio (61) leveraging the central tendency of only true GIS samples for achieving the final converged Log2(abundance) central tendency at 0 relative Log2(abundance) units. Then, the resulting abundance matrix was subset to include CTL (n = 46), AD (n = 49), and progressive supranuclear palsy (PSP, n = 26) cases. Age, sex, and postmortem interval covariates were then modeled and covariance removed *via* nonparametric bootstrap regression with the protein-specific coefficient for each modeled variable set to the median from 1000 bootstraps (49).

#### Weighted Gene Co-Expression Network Analysis

The weighted gene co-expression analysis (WGCNA) algorithm (62) was applied to execute co-expression network analysis to obtain modules of highly correlated protein groups of cases with neuropathological diagnoses of AD, PSP, and CTL, as described (18, 19, 52, 60, 63, 64). The WGCNA::blockwiseModules function was implemented for network generation. The following settings were used: soft threshold power = 9, deepSplit = 2, minimum module size = 25, merge cut height = 0.07, mean topological overlap matrix denominator, signed network with partitioning about medoids per the dendrogram, and a module reassignment threshold of *p* < 0.05. The signedkME function was also employed to determine biweight midcorrelation (bicor) of protein to module membership (Table S12). Proteins with high kME values are considered to be module hubs. Module i-Graphs were generated with the buildIgraphs function, available from https://www.github.com/edammer/netOps. i-Graphs with PPI database (BioGrid) integration contain information for sTurbo TauRD–interacting proteins across network hubs. Additional visualization layout and formatting for dataset hits were performed using Illustrator 2024.

#### FET Analyses

*Gene Ontology—*A hypergeometric overlap test was performed for each module protein's membership using the above-detailed GOparallel function and human reference GMT file.

##### Cell Type Marker Enrichment

One-tailed FET FDR adjusted -Log10^(*p*-values)^ for the enrichment of brain cell type marker proteins in human AD and PSP bulk frontal cortex proteomics modules were visualized as heatmaps using the single-list geneListFET function from open source code available online at https://www.github.com/edammer/CellTypeFET, as previously published (17, 18, 51, 55, 57, 59, 60, 65, 66, 67, 68, 69).

##### Cellular Compartment Enrichment Analyses

This geneListFET script was adapted to visualize the enrichment of module proteins to a curated list of subcellular components downloaded from Uniprot November 2024 (Subcellular localization GO-SL IDs: 0516, 0515, 0243, 0090, 0091, 0191, 0048, 0158, 0132, 0173, 0039).

##### Tau Interactome Mapping to Human Network

Human tissue tau interactomes (immuno-enriched proteomes) from human postmortem brain tissue (15, 16), neuronal tau AP studies (13, 14), and *in vitro* sTurbo TauRD interactors to human brain proteomic modules were separately considered as input lists for independent runs of the geneListFET against module membership.

##### Insoluble TMT Proteomics to Human Network

A previously published dataset of sarkosyl insoluble TMT proteomics from AD and CTL brains were mapped to the human network modules to define “insoluble mapping” proteins (70). Enrichment was visualized as a barplot of the -Log10^(unadjusted *p*-values)^. Insoluble mapping proteins were manually annotated within module-specific i-Graph(s) in Illustrator.

## Results

### Establishment of a Split-TurboID TauRD PL System in Cell Culture

**Fig. 1:**
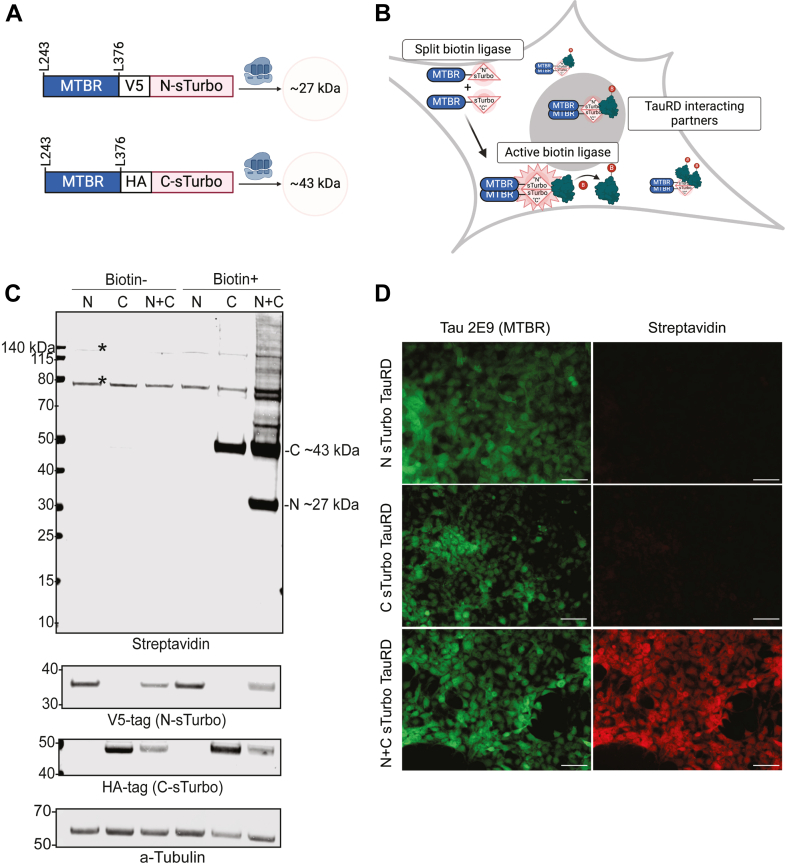
**sTurbo TauRD expression and biotin ligase activity in a human cell line.***A,* design of split-Turbo Tau repeat domain (sTurbo TauRD) and respective anticipated molecular weights. The N-sTurbo is 8 kDa, while the C-sTurbo is 27 kDa; upon interacting, these recapitulate the functional activity of the complete 35 kDa E.*coli* biotin ligase. A unique biochemical tag was added to this design to distinguish the two split enzymes: V5 for N- sTurbo and HA for C-sTurbo. Each construct contains a 133 amino acid sequence of the four-repeat containing microtubule-binding domain (MTBR) from Tau 243 to 376, 441 numbering system and houses a P301L pro-aggregation substitution. *B,* proposed model of sTurbo recombinant protein activity *in vitro*. Once the Tau MTBRs interact, the two sTurbo ligase fragments become functional and biotinylate proximal multimeric Tau protein interactors with or without the addition of 200 μM exogenous biotin. *C,* cotransfection with each split Turbo fragment displays robust biotinylation in the presence of exogenous biotin. sTurbo fragments were individually transfected (N or C) or cotransfected (N+C) into HEK293 cells. Groups were treated with serum-free media (Biotin−) or 200 μM biotin (Biotin+) for 1 h. Streptavidin-conjugated dye was used to probe for biotinylated proteins. Biochemical tags (V5 or HA) confirmed the recombinant protein expression, and a-tubulin was probed for verification of equal protein loading across lysates. The C-sTurbo fragment displays low-level biotinylation, as it houses the ligase active site. WB revealed robust biotinylation in cotransfected lysates. *Asterisks* indicate endogenously biotinylated protein bands observed at ∼73, 75, and ∼130 kDa. *D*, immunocytochemistry (ICC) of respective N- and C-fragments confirms WB results of biotin ligase activity upon recombinant protein co-expression. Tau 2E9 MTBR-specific antibody detected recombinant protein expression while streptavidin dye captured biotinylated interacting partners. Scale bars represent 50 μm. N-sTurbo, N-terminal split TurboID; C-sTurbo, C-terminal split TurboID; WB, Western blot.

The MTBR forms the core of NFTs in the brain, which is a major pathological feature across tauopathies, including AD (71, 72). Overexpression of the MTBR domain has been shown to induce aggregation in cell model systems (42, 43). Here, we sought to develop a TauRD split PL system to unbiasedly identify co-interacting partners associated with tau self-association and aggregation, similar to the well-characterized split-GFP TauRD HEK293 model systems (42, 73, 74). As shown in Figure 1*A*, we first generated two separate expression constructs, each encoding for 133 amino acids of the tau MTBR as the bait protein, fused to either the 8 kDa N-sTurbo or the 27 kDa C-sTurbo fragment. The N-sTurbo plasmid includes a V5 epitope tag, while the C-sTurbo is marked with an HA epitope tag. Upon co-expression, each construct is expected to produce a tau-MTBR that, when the two sTurbo biotin ligase fragments are brought into proximity, activates the biotin ligase enzyme, tagging proteins within a 10 nm range with intracellular biotin. This system enables the labeling of TauRD-interacting partners for downstream proteomic analysis, as depicted in the *in vitro* model (Fig. 1*B*).

To assess expression and biotin ligase activity, we first expressed the N- and C-terminal sTurbo TauRD constructs alone or together in a HEK293 cell culture system. Protein lysates from untreated (Biotin-) and biotin-treated (Biotin+) cells were analyzed by WB for streptavidin and for the recombinant tags for the TauRD harboring the N- and C-terminal sTurbo proteins (Fig. 1*C*). N-sTurbo was detected at ∼27 kDa by blotting for the V5 tag in transfected lysates, while the ∼43 kDa C-sTurbo TauRD fragment was detected by blotting for HA. The observed molecular weight of the sTurbo TauRD recombinant fragments may be influenced due to several factors contributing to deviations from their predicted molecular weights. Biotinylation adds 226 Da to each Lys, potentially impacting the electrophoretic mobility of the sTurbo TauRD fragments. In the absence of biotin, only endogenously biotinylated proteins were observed at ∼135 and ∼75 kDa. However, upon biotin treatment, we detected self-biotinylation of the C-sTurbo TauRD fragment at ∼43 kDa in cells expressing just the C-sTurbo TauRD recombinant protein. This partial biotin activity is consistent with prior studies (40, 41). In contrast, robust biotinylation of both the C-sTurbo and N-sTurbo TauRD fragments (∼43 and ∼27 kDa) was observed in cells only after co-expressing both constructs and treated with biotin. Furthermore, extensive protein biotinylation was detected across a wide molecular weight range with a streptavidin infrared label, indicating effective labeling of not only the sTurbo TauRD fragments but also potential interacting proteins. In a complementary approach, ICC was also performed for cells expressing either the N- or C-sTurbo TauRD fragments alone or in combination. The sTurbo TauRD fragments were detected with a specific MTBR-tau Ab (2E9), while biotinylated proteins were visualized with a streptavidin fluorescent dye. Imaging did not display biotinylation when the TauRD fragments were expressed alone; however, robust biotinylation was clearly seen upon cotransfection (Fig. 1*D*). The absence of detectable biotinylation by ICC for C-sTurbo tau alone, compared to detection by WB, may be due to differences in assay sensitivity. Collectively, results from both WB and ICC analyses confirm that the sTurbo TauRD PL system successfully labels tau interacting partners when both N- and C-sTurbo fragments are co-expressed, with minimal self-biotinylation occurring when fragments are transfected separately.

### Development and Validation of a Bicistronic sTurbo TauRD Construct for Uniform Expression, Functional PL, and Aggregation in HEK293 Cells

**Fig. 2:**
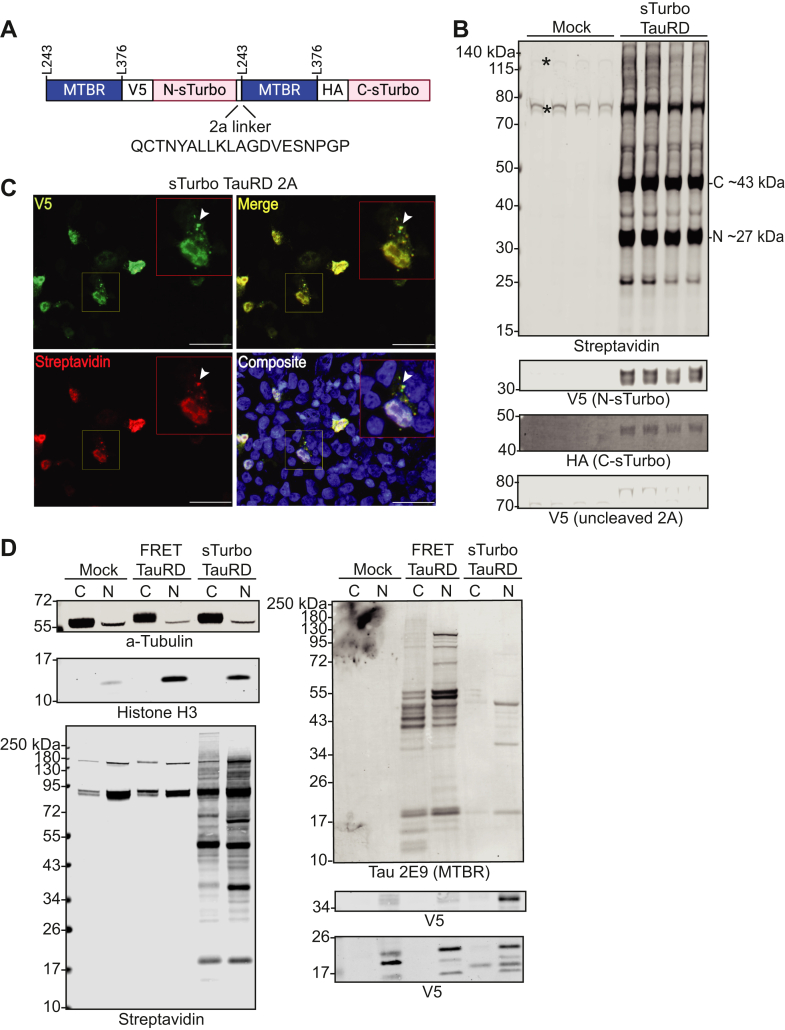
**Development and characterization of a bicistronic sTurbo TauRD construct and functional proximity labeling.***A,* design of split ligase construct utilizing a single 2A plasmid to mitigate cotransfection efficiency variations. An e2A self-cleaving linker enables a 1:1 stoichiometric expression of each ligase fragment in the same cell. *B,* confirmation of biotin ligase expression and activity in HEK293 cells transfected with a single plasmid sTurbo TauRD *via* WB analysis. The N- and C-sTurbo fragments exhibit reactivity to their respective biochemical tags and demonstrate self-biotinylation with only a weak proportion of uncleaved recombinant protein demonstrated at ∼75 kDa in the V5 channel. Transfection reagents without plasmid (mock) reveal endogenously biotinylated proteins at ∼130, ∼75, and ∼73 kDa, indicated by *asterisks*. *C,* ICC displays Tau-positive inclusions (*white arrows*), overlapping with both nuclear DAPI signal (*blue*), and extranuclear signal. Recombinant protein largely colocalize with biotinylated proteins (*yellow*). Rabbit anti-V5 tag (*green*) targets the sTurbo TauRD recombinant protein, while streptavidin dye (*red*) portrays biotinylated proteins. Images were captured at 40× magnification. Scale bars represent 50 μm. *D,* subcellular fractionation of HEK293 cells that were transfected without plasmid (mock), FRET TauRD bicistronic plasmid, or sTurbo TauRD. Crude fractionations were confirmed by probing for subcellular compartment markers, a-Tubulin and Histone H3 for cytoplasmic and nuclear compartments, respectively. sTurbo TauRD showed robust biotinylation across both cytoplasmic and nuclear fractions. Recombinant TauRD, detected by MTBR-specific antibody, Tau 2E9, for both FRET TauRD and sTurbo TauRD, with the addition of V5 tag for sTurbo TauRD protein, was abundant in the nuclear fraction. N-sTurbo, N-terminal split TurboID; C-sTurbo, C-terminal split TurboID; TauRD, tau microtubule repeat domain; WB, Western blot; ICC, immunocytochemistry; MTBR, microtubule-binding repeat domain.

To overcome nonuniform cotransfection and, consequently, differences in gene expression within and across experimental groups, we generated a bicistronic plasmid containing both N- and C-sTurbo TauRD fragments separated by a 2A self-cleaving linker (75). As illustrated, the 2A linker was inserted after the N-sTurbo and before the subsequent MTBR domain (Fig. 2*A*). Biotin ligase expression and activity in HEK293 cells transfected with single sTurbo TauRD bicistronic plasmid was confirmed by WB. The N- and C-sTurbo biotinylated TauRD fragments were observed at their expected molecular weights, ∼27 and ∼43 kDa, respectively, whereas transfected cells only displayed endogenously biotinylated protein bands at ∼135, 78, and 75 kDa (Fig. 2*B*). Additionally, a band at ∼75 kDa reacted with the V5 Ab but was not detected by HA (Fig. S1*A*). Prior reports have described a potential overabundance bias toward the first coding sequence of some 2A linkers (76). To address this, a semiquantitative WB was used to measure the biotinylated N- and C-sTurbo TauRD protein signal intensities across four replicates. The N- to C-terminal ratios were approximately 1:1, indicating minimal labeling bias between fragments due to uncleaved 2A (Fig. S1*B*).

To assess localization and biotinylation activity of the recombinant proteins, we performed ICC and subcellular fractionation. sTurbo TauRD localized throughout both DAPI-positive nuclei and extranuclear compartments, often forming speckle-like inclusions (77). These inclusions largely overlapped with the biotinylation signal (white arrows), likely due to tau self-biotinylation and biotin-labeled proximal interactors (Fig. 2*C*). Subcellular fractionation confirmed localization of biotinylated protein and recombinant protein signal. Subcellular fractions were confirmed by respective markers for C and N compartments by α-Tubulin and histone H3, respectively. sTurbo TauRD show differential biotinylated protein banding across C and N compartments, whereas recombinant protein, as detected by V5 (TurboID) and Tau-MTBR-specific Ab (2E9), displayed predominant nuclear signal (Fig. 2*D*). This was orthogonally confirmed with comparison to a FRET TauRD, where the Tau-MTBR and GFP Ab, detecting both fluorophores (cyan and yellow fluorescent proteins), recapitulated the sTurbo TauRD subcellular expression profile, and displaying a potential nuclear bias of the isolated TauRD in HEK cells (Fig. S1*C*).

Given the inclusions observed by ICC, the oligomerization propensity of sTurbo TauRD was addressed biochemically by Blue native PAGE (BN-PAGE) and Thioflavin T *in vitro* assays (Fig. S2). BN-PAGE analysis showed that sTurbo TauRD constructs with the P301L mutation and without (WT) formed high molecular weight species, albeit to a lesser degree, than the isolated aggregate prone recombinant K18 (4R TauRD) and was unaffected by the addition of biotin. In contrast, full-length tau did not exhibit high molecular species on BN-PAGE, consistent with prior reports (78) (Fig. S2*A*). In a complementary assay, all TauRD lysates, including FRET, sTurbo P301L, seeded the isolated recombinant K18 protein and led to increased Thioflavin T fluorescence in a sigmoidal kinetic curve, indicative of fibril formation, consistent with the recombinant K18 P301L fragment alone (Fig. S2, *B–D*). Taken together, these complementary results suggest that the addition of sTurbo enzyme appendages does not grossly alter the aggregation propensity of the TauRD in comparison to FRET TauRD, although both recombinant proteins show a lesser degree of fibrillization than the K18 P301L fragment alone.

### PL of Tau MTBR Interactors and MS Analysis in Cells Reveals Established and Novel Tau-Associated Pathways

**Fig. 3:**
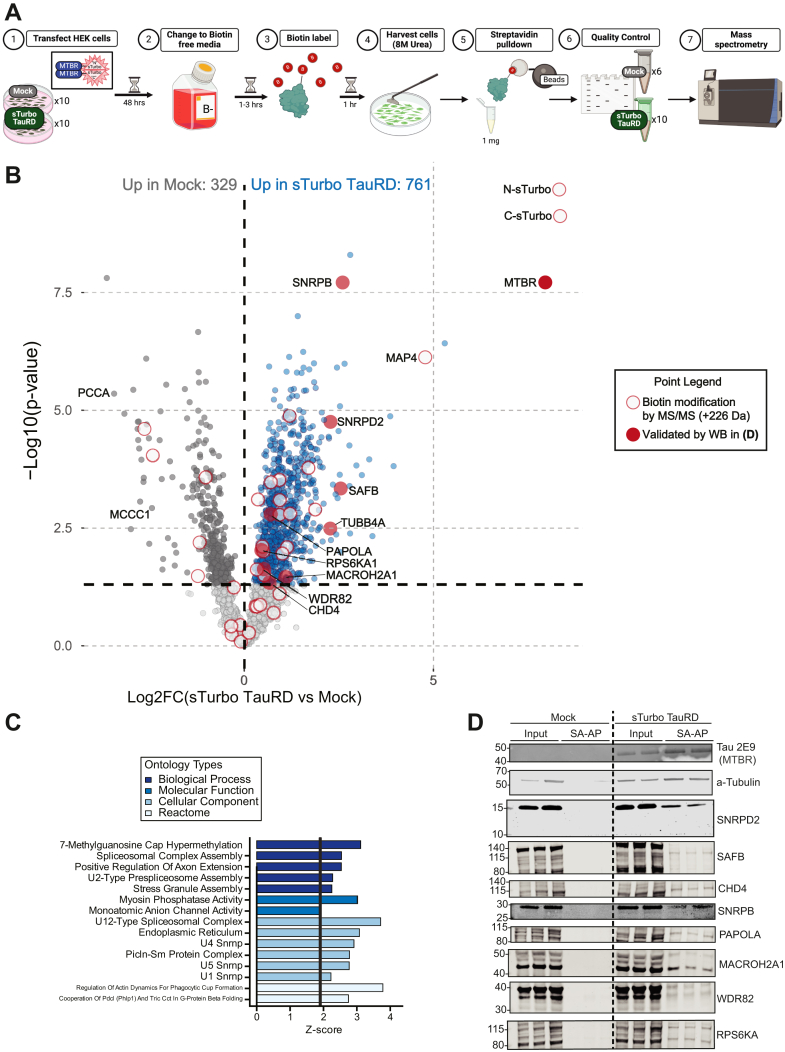
**Differential abundance analysis of TauRD proteomics demonstrates nuclear protein enrichment.***A,* schematic experimental workflow for identifying protein–protein interactors of the MTBR domain of Tau. Ten replicates of HEK293 cells across three batches were transfected, underwent a biotin washout period, labeled with 200 μM biotin for 1 h, and harvested in 8M Urea with protease inhibitors. 1 mg of total protein from whole cell lysate was incubated with streptavidin-conjugated magnetic beads for 1 h to enrich for biotinylated protein interactors. Samples underwent quality control by total protein silver stain. Biotinylated proteins were investigated *via* mass spectrometry*. B*, mass spectrometry identified over 700 significant proteins in sTurbo TauRD lysates compared to controls. Volcano plot displays the Log2-Fold change between groups plotted on the x-axis while –Log10 unadjusted *p*-value, as determined by one-way ANOVA, is plotted on the y-axis. A *p* < 0.05 (−Log10 = 1.3) was employed to identify potential candidate proteins for validation. *Blue points* indicate proteins significantly enriched in the sTurbo TauRD samples (n = 761), while *dark gray dots* indicate proteins enriched in the mock samples (n = 329). Proteins with biotin modification identified by mass spectrometry mass shift (+226 Da) are represented by open *red points* while enriched proteins in sTurbo TauRD validated by Western blot in *panel D* are labeled in closed *red points*. Mock-enriched proteins are related to endogenously biotinylated carboxylases, labeled in the plot, and contaminating keratin proteins. *C,* top Gene Ontology (GO) terms for these putative TauRD interacting proteins (n = 761) confirmed various pathways, including cytoskeletal component and RNA binding. *D,* canonical TauRD interacting proteins, such as the Tau-MTBR itself and a-Tubulin, were selected for validation and served as positive controls. New candidate proteins, including SAFB, CHD4, SNRPB, PAPOLA, MACROH2A1, WDR82, and RPS6KA, were additionally validated by WB. Total lysate input is equal across both mock and sTurbo TauRD. Only the sTurbo Tau-expressing samples maintained interactions with these proteins upon streptavidin affinity purification. TauRD, tau microtubule repeat domain; WB, Western blot; MTBR, microtubule-binding repeat domain; sTurbo, split TurboID.

Ab-based affinity purification coupled to mass spectrometry (AP-MS) studies have provided a breadth of typical and pathophysiological interactors of tau from both cells and tissues, including RBPs, 14-3-3 binding proteins, numerous cytoskeleton components, heat-shock proteins, and proteasome subunits (13, 14, 15, 16). Here, we sought to unbiasedly identify interacting proteins of the tau MTBR using our sTurbo expression system. To this end, HEK293 cells (n = 10 biological replicates) underwent a standard SA-AP and label-free quantitative MS workflow, as previously described (35, 38), and outlined in Figure 3*A*. As expected, sTurbo TauRD lysates showed consistent and robust biotinylation across replicates, including self-biotinylation of sTurbo TauRD fragments (Fig. S3*A*). To confirm pulldown upon SA-AP, gel electrophoresis and silver staining was performed from each eluent, which confirmed the enrichment of proteins in sTurbo TauRD samples as well as nonspecific protein binding to streptavidin beads in the mock samples, that were omitted from further investigation (Fig. S3, *B* and *D*). Following WB and silver stain quality control assays, Mock (n = 6) and sTurbo TauRD (n = 10) pulldowns were digested directly on beads for downstream proteomic analysis (35, 38). Following label-free quantitative MS, resulting intensities advanced through an in-house AP-MS pipeline, including quality control and imputation for missing values (34, 35, 38) as described in the methods (Tables S4, S6, S7, S8, S13–S18). Unique MTBR peptides along with those corresponding to the custom N- and C-sTurbo FASTA sequences were also identified which confirmed biotinylation and pulldown of the expected TauRD (Table S19). Overall, 2167 proteins were retained for subsequent differential enrichment analyses (DEAs).

DEA revealed 1090 total significantly different proteins; 761 were increased in sTurbo TauRD pulldowns, whereas 329 proteins were enriched in Mock pulldowns (Fig. 3*B*). As anticipated, the overexpressed recombinant sTurboID N- and C-terminal-specific proteins and the core TauRD domain (shared between the N- and C-term fragments) were most significantly enriched in pulldowns with Log2-fold change (Log2FC) of 8.3, in both fragments with a FDR corrected *p*-value of 8.12E-07 in C-sTurbo and 4.44E-07 in N-sTurbo fragments. Importantly, the similarity of FC enrichment across the fragments verifies the 1:1 expression ratio of the 2A products. Consistently, the TauRD which is translated contiguously with the N- or C-term sTurboID fragments had a Log2FC of 7.95 (FDR: 7.04E-06) (Tables S20 and S21). Biotin modifications identified by MS were mapped to 52 peptides, including regions within the sTurbo fragments and the MTBR, indicating direct interactors of the TauRD (Table S18**)**. GO analysis of mock-enriched proteins was associated with vitamin binding (biotin metabolism) and skin epidermis (keratins) consistent with enrichment of endogenously biotinylated proteins as would be expected in mock samples (35). In contrast, the proteins enriched by sTurbo TauRD included the MTBR fusion proteins, as well as other proteins with known tau biology, including interactions with microtubule subunits and an over-representation of RBPs associated with stress granules (G3BP1, CSDE1, DDX6, UBAP2L, and YTHDF3) and the spliceosome complex (15, 16, 21, 22, 24, 25, 26, 28, 29, 63, 79, 80, 81, 82) (Fig. 3*C*, Table S22).

To confirm the enrichment of biotinylated proteins and validate the MS results, WB was performed on several candidate proteins following sTurbo tau-MTBR pull-downs. This included canonical tau-interacting proteins, such as Tau-MTBR (detected using the Tau 2E9 Ab) and α-Tubulin (3, 4, 83, 84), to compare enrichment levels across input lysates and pulldown (SA-AP) eluents. The RBP SNRPD2 was enriched in the sTurbo TauRD lysates and has previously been shown to associate with tau in AD (16). Additional RBPs representing GO-enriched processes (SAFB, CHD4, SNRPB, PAPOLA, WDR82, and RPS6KA1) were also confirmed to be enriched in sTurbo TauRD SA-AP but not negative mock-transfected CTLs (Fig. 3*D*). Of note, several of these proteins are newly validated tau-MTBR interactors presented in this study (PAPOLA, MACROH2A1, RPS6KA1, WDR82, and CHD4). We further compared the TauRD interactome to those of two other intracellular aggregating proteins implicated in neurodegeneration: fused in sarcoma (85) and TAR DNA-binding protein-43 (86). Only four proteins were shared across all three interactomes. sTurbo TauRD shared 97 proteins with the TAR DNA-binding protein-43 interactome and 20 with the fused in sarcoma interactome, while more than 630 proteins were uniquely enriched in the TauRD interactome (Table S23). These findings underscore the specificity of RBP interactomes with the TauRD.

**Fig. 4:**
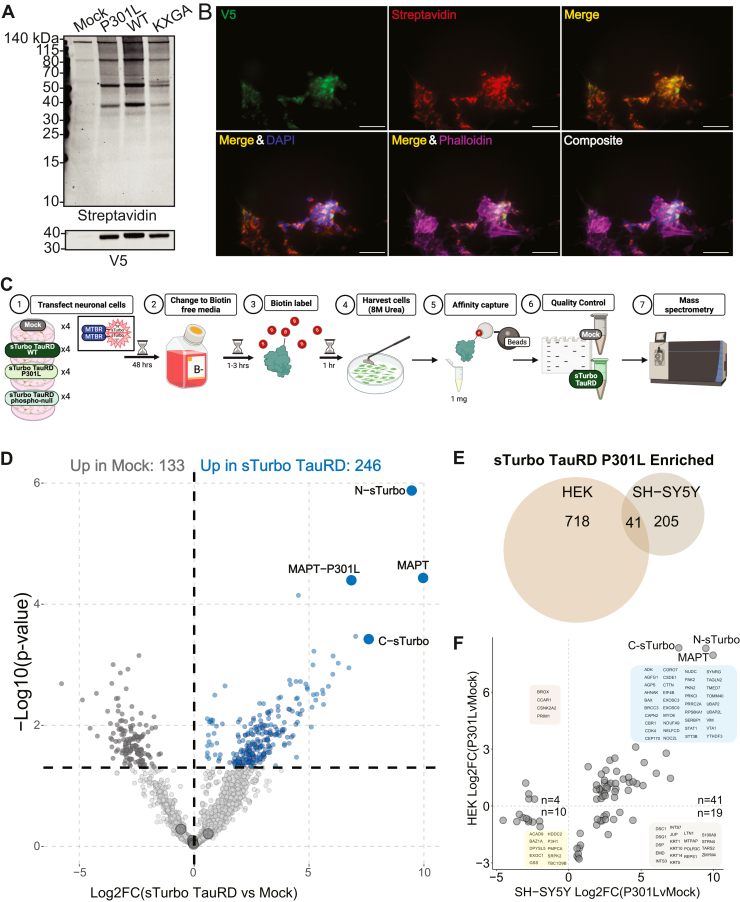
**sTurbo TauRD implementation in neuronal cell line displayed consistency to non-neuronal cell interactors and biological processes.***A,* Western blot confirmation of biotin ligase activity (Streptavidin) and recombinant sTurbo TauRD protein expression (V5 tag) in SH-SY5Y cells across three independent sTurbo TauRD constructs (P301L, WT, KXGA phosphorylation null). *B,* immunocytochemistry of SH-SY5Y cells detecting recombinant sTurbo TauRD (V5) and biotinylated proteins *via* streptavidin dye. sTurbo TauRD and potential co-aggregating proteins show overlap (Merge). In SH-SY5Y cells, biotinylated proteins did not overlap with nuclear signal (DAPI) while colocalizing with the cytoskeleton (phalloidin dye; actin localization). Scale bar represents 50 μM. *C,* experimental workflow for identifying TauRD interactors in SH-SY5Y cells with the addition of experimental conditions: wildtype, no P301L substitution (WT) and phospho-null mutation in KXGS motifs (KXGA). Four biological replicates of SH-SY5Y cells were transfected per experimental condition and underwent the same workflow as seen in prior HEK293 cell preparation and proteins identified by high resolution data-independent acquisition mass spectrometry*. D,* mass spectrometry identified 246 significant proteins in sTurbo TauRD P301L lysates compared to negative controls (mock). Volcano plot displays Log2-fold change between sTurbo TauRD and mock negative control on the x-axis while –Log10 unadjusted *p*-value, as determined by one-way ANOVA, is plotted on the y-axis. *Blue points* indicate proteins significantly enriched in the SH-SY5Y sTurbo TauRD interactome, while *dark gray* points indicate proteins enriched in the mock samples (n = 133). Large *gray points* at ∼ Log2FC = 0 are endogenously biotinylated carboxylases. *E,* overlap of all sTurbo TauRD P301L enriched proteins across HEK and SH-SY5Y cells. Forty-one proteins were consistent between the two interactomes. *F,* integration of significant and shared mock and sTurbo TauRD P301L proteins across HEK and SH-SY5Y display a largely consistent Log2FC with 51 concordant and only 23 proteins discordant across cell type enrichments (HEK n = 4; SH-SY5Y n = 19). TauRD, tau microtubule repeat domain; sTurbo, split TurboID; *MAPT,* microtubule-associated protein tau.

We next investigated the tau interactome in a neuronal context using SH-SY5Y cells. WB analysis revealed a biotinylation profile similar to that observed in HEK cells (Fig. 4*A*). In SH-SY5Y cells, biotinylated proteins and recombinant sTurbo TauRD colocalized more with cytoskeletal actin (phalloidin dye) than nuclear DAPI (Fig. 4*B*). To define the neuronal TauRD interactome, we applied a similar workflow to that used in HEK293 cells. Four conditions were prepared in biological replicate (n = 4) for MS (Fig. 4*C*, Table S24). This yielded 5397 identified proteins, with 4083 retained after filtering for 50% groupwise missingness (Tables S3, S5, S9, S10, S25, & S26). DEA of P301L and mock conditions identified 379 significantly different proteins: 246 enriched in SH-SY5Y sTurbo TauRD P301L samples and 133 enriched in mock SA-AP CTLs (Fig. 4*D*, Table S27). Among the TauRD-enriched proteins, 41 overlapped with those found in HEK293 cells. Importantly, these include proteins predominantly involved in RNA-binding, stress response, and the cytoskeletal organization, consistent with SH-SY5Y P301L-enriched GO terms (Fig. 4, *E* and *F*, Table S28).

As HEK293 cells revealed phosphorylation within the MTBR KXGS motifs at residues S262 and S356 (Fig. S4, *A*–*C*), we additionally incorporated a phosphorylation-null mutant (KXGA) sTurbo TauRD to assess the functional impact of phosphorylation on the interactome (Fig. S4, *A*–*C*). Of note, the KXGS motifs are the most frequently phosphorylated residues within the MTBR and are critically important regulators for microtubule binding, aggregation, and phosphorylation-dependent functions (87, 88, 89, 90, 91). DEA of the phospho-null mutant condition, P301L-KXGA, and P301L-KXGS sTurbo TauRD identified 429 significantly different proteins: 111 enriched in P301L-KXGS samples and 318 enriched in P301L-KXGA (Fig. S4*C*, Table S29). Six kinases were enriched in the P301L-KXGS sTurbo TauRD pulldown, including those previously implicated in pathology and disease processes: MARK3 (92), MINK1 (93), TLK2 (94), and PRKCi (95). Kinases enriched in KXGA mutant included those associated with homeostatic cell division and the cellular stress response DTYMK (96), MAP4K5 (97), CKMT1B (98), and AKT1 (90, 99). GO analysis of these enriched proteins complement these findings, summarizing ontologies related to apoptosis in the P301L-KXGA phospho-null condition, whereas P301L-KXGS described modification of proteins and N compartments (Fig. S4, *F* and *G*, Table S28).

**Fig. 5:**
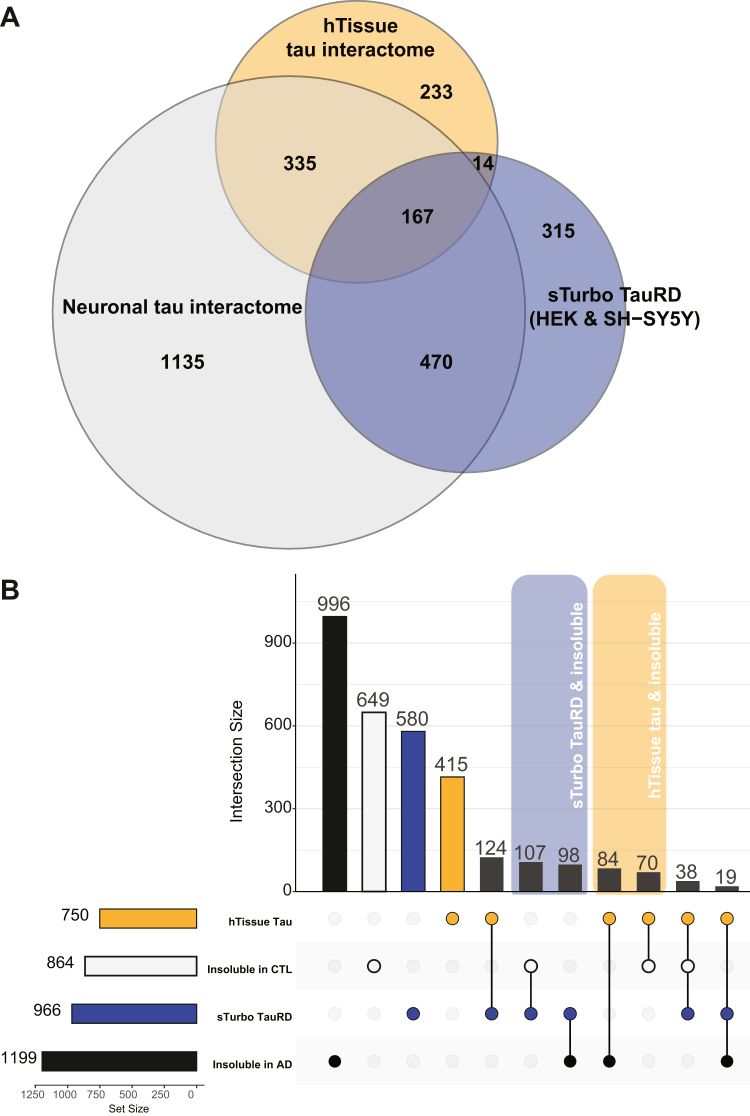
**Intersection of tau interactome studies reveals sTurbo TauRD proteins contain both shared and unique interactors.***A,* Venn diagram of enriched proteins across sTurbo TauRD (*blue*), combined antibody-based affinity purification of tau from human tissue (n = 6, hTissue, *orange*), and neuronal tau interactome studies (n = 8, *gray*). One hundred sixty-seven proteins were shared across all studies. *B*, UpSet plot representing unique and overlapping proteins identified in sTurbo TauRD proteomics (*blue*) and hTissue (*orange*) compared to proteins enriched in the sarkosyl-insoluble fraction of non-demented control (*white*) and AD brains (*black*). Intersections with sTurbo TauRD and insoluble proteomics are labeled in *blue* while hTissue intersections are annotated in *orange*. TauRD, tau microtubule repeat domain; sTurbo, split TurboID; AD, Alzheimer's disease.

To assess the relevance of our *in vitro* tau interactors to previously identified human-relevant tau interactors, we compared our sTurbo TauRD interactors to six human brain datasets derived from AP-MS from four distinct studies using tau Abs (13, 15, 16, 100), as well as nine full-length neuronal tau interactomes from three unique studies (Table S30–S32) (14, 79, 101). Overall, 167 proteins were consistently enriched across sTurbo TauRD and the complementary AP-MS human and neuronal tau interactomes, with sTurbo TauRD identifying 315 distinct interacting proteins (Fig. 5*A*, Table S33). Interacting proteins identified through PL and biotin enrichment may have been insoluble or transiently associated with pathological proteins (102) and therefore missed in human interactome datasets generated by standard Ab-based IP. This sTurbo TauRD PL approach identified over 200 unique tau interacting insoluble-enriched proteins (Fig. 5*B*, Table S33). Specifically in many tau interactome datasets to date (13, 14, 15, 16, 21, 36, 79, 100, 101, 103), PL studies have mapped significantly more insoluble enriched proteins than Ab AP approaches, 365 PL insoluble proteins in comparison to 16 Ab insoluble (Fig. S5, Table S34). In summary, this proteomic assay reveals both known and distinct interactors of tau-MTBR, reflecting shared tau biological functions across previously identified human tau interacting partners and model systems and has capabilities to capture insoluble, co-aggregating interacting proteins.

### Network Analysis of Human AD and PSP Brain Tissue Identifies Protein Modules Associated With Tau Pathology

**Fig. 6:**
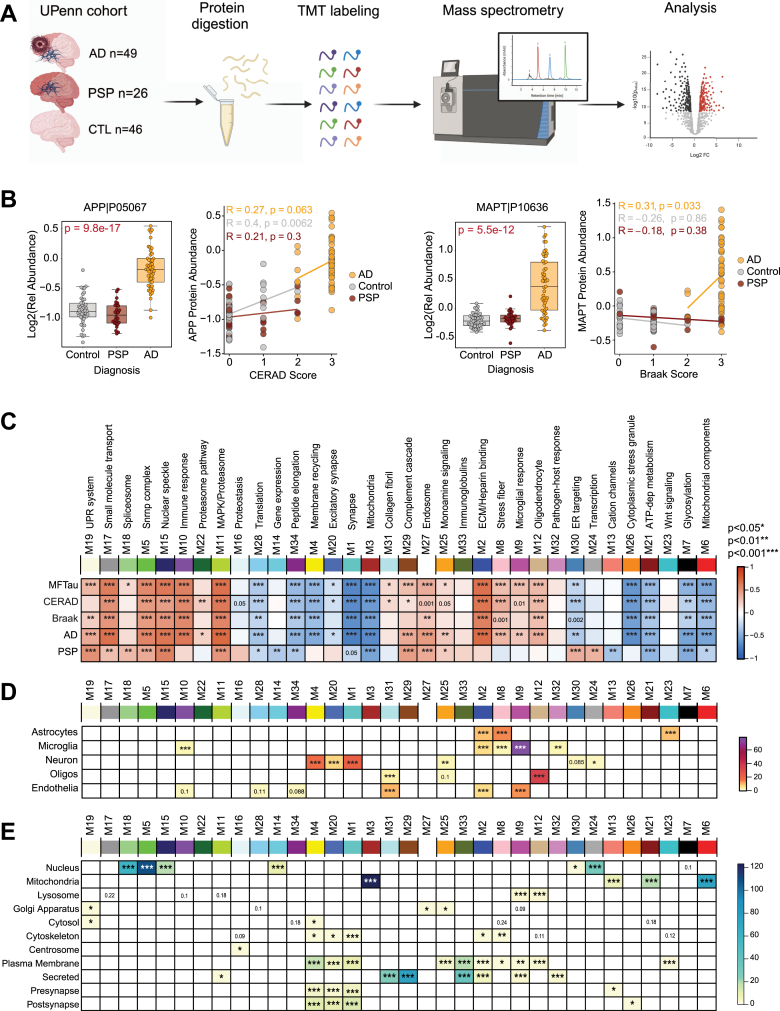
**Network analysis of human AD and progressive supranuclear palsy (PSP) brain tissue.***A,* schematic of University of Pennsylvania (UPenn) brain tissue selection and experimental overview. AD (n = 49), PSP (n = 26), and CTL (n = 46) frontal lobe tissues underwent proteolytic digestion, TMT labeling, and mass spectrometry. A relative protein abundance matrix was used for downstream analysis. *B,* quality control measures (CERAD and Braak) across PSP and AD groupings. APP levels across groups are related to increased amyloid burden (CERAD) in AD cases but not PSP. Similarly, MAPT levels are highest in AD and correlate to higher Braak staging in disease. PSP cases do not display increased amyloid deposition or frontal lobe Tau burden. *C,* AD and PSP proteins (n = 9661) were analyzed *via* weighted gene network analysis (WGCNA) and separated into 34 highly related protein co-abundance modules. Each module name is summarized from significant Gene Ontology (GO) terms. Those highly related to AD include M2 (ECM/heparin-binding) and M5/M15 (Snrnp complex, nuclear speckle). Signed bicor r-value of module abundance to Tau pathology (MFTau, Braak), amyloid burden (CERAD) and diagnosis. *Red* represents a positive correlation, while *blue* describes a negative correlation (∗, *p* < 0.05; ∗∗, *p* < 0.01; ∗∗∗, *p* < 0.001). *D,* neural cell type marker enrichment by Fisher's Exact Test (FET) to modules. FDR corrected, Benjamini-Hochberg (BH) –Log10(*p*-values visualized. *E*, subcellular localization marker enrichment by FET to modules. −Log10(FDR, BH *p*-values) visualized. APP, amyloid precursor protein; AD, Alzheimer's disease; *MAPT,* microtubule-associated protein tau; TMT, tandem mass tag; FDR, false discovery rate.

To further contextualize the association of the TauRD interacting partners to disease, we integrated the tau interactome data with previously generated proteomics data from human postmortem bulk frontal cortex samples (104), including CTL (n = 46), AD (n = 49), and PSP (n = 26) (Fig. 6*A*, Table S35). After data quality control, this TMT MS-based human dataset consisting of 9641 proteins was analyzed using established pipelines for systems-level network analysis (18, 52, 60, 105) (Table S36).

As expected, amyloid precursor protein (APP), which in bulk AD brain proteomic datasets has been previously shown to be a proxy for β-amyloid (Aβ) deposition (64), was significantly higher in AD cases than CTLs and PSP. Elevated APP levels also positively correlated to neuropathological Aβ burden (CERAD score). However, as expected, APP levels between CTL and PSP cases did not show any differences in relative abundance (52, 64). Braak staging, a metric characterizing both the severity and localization of pathological tau spread throughout the brain, was significantly elevated in AD. MAPT protein abundances also correlated to pathological tau burden (Braak stage) (106). Meanwhile, this change was not observed in PSP or CTL cases (Fig. 6*B*). While PSP also exhibits tau tangle pathology, the highest burden remains in the temporal lobe rather than the frontal lobe region analyzed by TMT-MS based proteomics.

To gain more insight into system-level changes correlating to disease and pathology, a WGCNA pipeline for network analysis was applied to ascertain modules of highly co-expressed proteins across all tissues (62). This approach resulted in 34 modules, with M1 being the largest (n = 881 proteins) and the smallest being M34 (n = 35) (Table S12). Significant biological processes for each module were identified through GO analysis and named to summarize a representative ontology or cell type involvement (Table S37). Correlation analysis of key pathological traits, including immunological tau values (Mid-frontal, MFTau), CERAD score, Braak staging, and diagnosis of AD or PSP, reveal significant modules relating to disease processes (Fig. 6*C*).

Modules uniquely increased in AD were enriched in inflammatory brain immune protein markers, including those specifically expressed in astrocytes, microglia, and endothelia (M10, M2, M8, and M9) (Fig. 6*D*, Table S38) consistent with previous reports (18). Many of the AD-associated modules also corresponded to proteins localized to the “Golgi Apparatus”, “Cytoplasm” and “Plasma Membrane” (Fig. 6*E*, Table S39). Modules positively correlating to PSP were enriched in nuclear proteins (M18, M5, M15, M30, and M24). Consistent with prior reports (17, 21, 25, 31, 107, 108, 109, 110) of shared biology across different tauopathies, M18, M5, and M15 enriched in nuclear proteins were increased in both AD and PSP. Overall, we identified several modules that positively correlate with both AD and PSP and are associated with neuropathological tau burden. However, due to the limitations of bulk cortex proteomics, it remains unclear whether the proteins within these modules directly interact with tau.

### Integrating the TauRD Interactome With AD and PSP Network Modules to Identify Disease-Associated Modules Linked to Tau PPIs

**Fig. 7:**
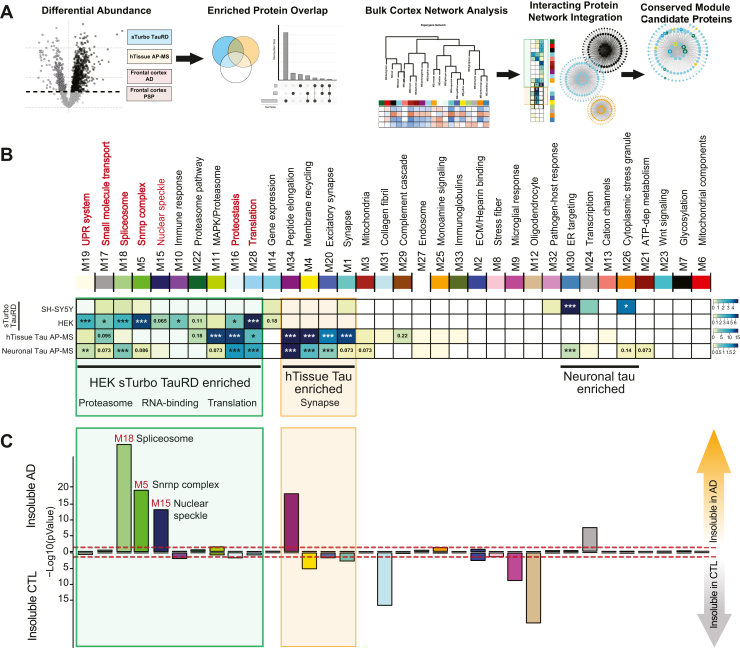
**Integration of the TauRD interactome with a human tauopathy proteomic network identifies RNA-binding protein modules associated with AD and PSP that are enriched in insoluble proteins.***A,* overview of data integration and visualization steps. A differential abundance of proteins in TauRD (*blue*), hTissue Tau (*orange*), and human AD & PSP bulk frontal cortex proteomics from tauopathies datasets was conducted. Total protein overlap was visualized by Venn Diagram and UpSet plots. Then, enriched tau AP-MS studies (*blue* & *orange*) were integrated into the human AD & PSP bulk frontal cortex proteomics network to determine protein overlap across modules. Tau AP-M5 overlapping proteins were also visualized by their degree of module membership in modules highly correlating to disease. *B,* FET of enriched protein overlaps with tauopathy brain network modules across sTurbo TauRD cellular interactomes, shown within a *green square*. Seven modules were significantly enriched in HEK293 sTurbo TauRD enriched proteins. Disease-associated proteosome modules M22 & M11 were also indicated in hTissue Tau interactomes with trends toward significance in sTurbo TauRD. Two modules, M16 & M28, were enriched within both sTurbo TauRD and hTissue Tau interactors. Many significant hTissue Tau and neuronal cell interactome modules discordant to sTurbo TauRD (M34, M4, M20, & M1; highlighted with an *orange square*) were enriched in synaptic proteins. FDR corrected, Benjamini-Hochberg (BH) –Log10(*p*-values) visualized per dataset (∗, *p* < 0.05; ∗∗, *p* < 0.01; ∗∗∗, *p* < 0.001). *C*, FET of brain network modules to the insoluble proteome from TMT-labeled AD brain and CTL brain lysates reveals modules related to sTurbo TauRD are generally insoluble protein enriched and related to proteosome, RNA-binding, and translation ontologies. –Log10(unadjusted *p*-values) are visualized. AD, Alzheimer’s disease; TMT, tandem mass tag; FDR, false discovery rate; TauRD, tau microtubule repeat domain; sTurbo, split TurboID; FET, Fisher's exact test; CTL, control; AP-MS, antibody-based affinity purification coupled to mass spectrometry.

To assess whether tau interactors are enriched in disease-associated protein modules in human AD and PSP, we integrated the recombinant sTurbo TauRD and human brain tissue (hTissue) tau interactomes with the cortical AD and PSP networks. To this end, a hypergeometric FET was used to identify modules in the human AD and PSP co-expression network that were significantly enriched with tau interactors from the sTurbo TauRD and human tau interactome datasets. (Fig. 7*A*, Table S40). Notably, no co-expression modules were consistently enriched across all tau interactome datasets, likely reflecting differences in cell type, tau enrichment strategies, and source materials. Interactors from sTurbo TauRD in HEK293 cells showed significant enrichment in seven modules (M19, M17, M18, M5, M10, M16, and M28). Interactors from SH-SY5Y cells mapped to several of the same modules observed in HEK293 and neuronal tau studies (M17, M18, and M5), though without reaching statistical significance. Uniquely, SH-SY5Y interactors were significantly enriched in modules M30 and M26, highlighting potential cell type–specific aspects of the tau interactome. hTissue tau interactors showed significant overlap with two of the sTurbo TauRD enriched modules (M16, Proteostasis; M28, Translation) as well as enrichment in M11 (Proteasome/MAPK signaling) but did not display significant overrepresentation in five of the sTurbo TauRD modules (M19, M17, M18, M5, and M10) (Fig. 7*B*). As expected, several tau interactors from hTissue and differentiated cell lines were uniquely enriched in neuronal and synaptic modules containing proteins that are not strongly expressed in HEK293 cells nor undifferentiated SH-SY5Y cells (Fig. 7*B*).

Because conventional Ab-based IP cannot readily affinity-capture insoluble protein interactions (37), we hypothesized this may be due to the insoluble nature of module proteins. We further examined whether the sTurbo TauRD interactors we identified were enriched in the insoluble human AD proteome using previously published data (Tables S34 and S41) (70). This analysis identified several disease-associated modules, specifically M18, M5, and M15, that overlapped with the insoluble AD proteome (Fig. 7*C*). These modules include proteins such as hnRNPs (HNRNPA2B1 and TARDBP) (30, 110) and spliceosome members (SNRPD1, SNRPD2, and SNRPE) (16). Notably, M18, M5, and M15 were significantly enriched in RBPs localizing across the nucleus, nuclear speckles, and cytoplasm. These modules relate to splicing regulation function and RBPs containing canonical RNA recognition motif domains (Fig. S6). M18 and M5 have the most similarity across RBP types: viral RNA regulation, spliceosome, RNA stability & decay, and nuclear export and translation regulation functions are all represented. These modules also slightly differ in types of enriched RBPs. M5 houses RBPs uniquely mapping to nucleolus, M15 is the only disease-related insoluble nuclear module with Zinc Finger domain-containing proteins represented, and M18 RBPs are also involved in 3′ end processing of pre-mRNA (111). Overall, the sTurbo TauRD interactome overlapped with three tauopathy-associated modules that were not enriched with human tau interactors, likely in part due to the enrichment of insoluble nuclear proteins relating to histone and DNA-binding, nuclear transport, polyadenylation factors, and splicing.

**Fig. 8:**
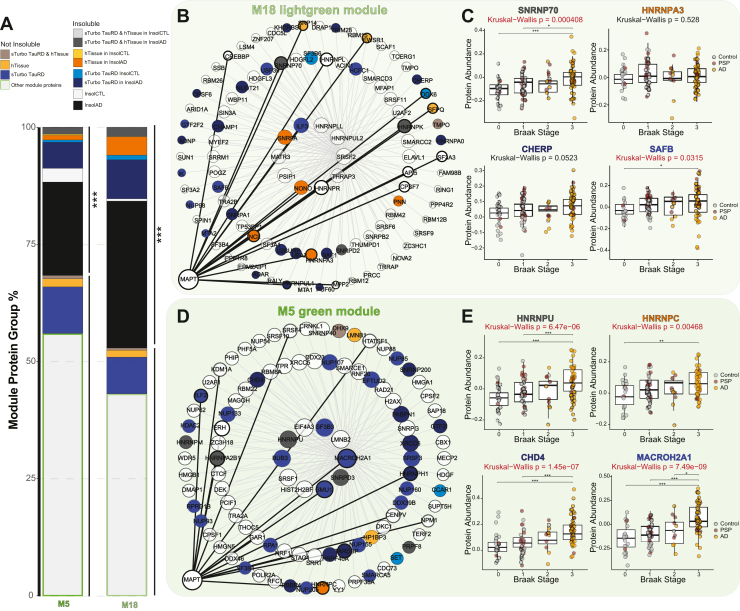
**TauRD-associated interactions within insoluble RBP modules (M18 & M5) reveal hub proteins connected to mRNA surveillance across Braak stages.***A*, stacked barplot displaying percentage of module members intersecting with Tau AP-MS and sarkosyl-insoluble enriched proteomics in M18 & M5 modules, colored by intersection type in above legend. Broadly, *blue* represents sTurbo Tau interacting partners, *orange* represents hTissue, and *gray*/*brown* are shared interactors across datasets. ∼50% of all M18 proteins mapped to insoluble proteins with sTurbo TauRD accounting for 11% of insoluble interactors while hTissue represented less than 5% (3.92%). M5 module members consisted of 28.7% insoluble-mapping proteins, of which 7.5% were sTurbo TauRD interactors, and 2.68% were identified in hTissue tau. *B–E,* i-Graph representation and visualization of candidate hub protein abundances were plotted across Braak stages. A Kruskal–Wallis rank sum test is displayed across all Braak stages while a Wilcoxon rank-sum test was implemented at individual pairwise comparison levels and significance denoted (∗, *p* < 0.05; ∗∗, *p* < 0.01; ∗∗∗, *p* < 0.001). Thick edge lines of i-Graphs provide information on biological interactions of hub proteins to MAPT in disease-related modules, M18 (B–C) and M5 (D–E). Protein nodes are colored according to the legend if tau interacting proteins are identified within the module (sTurbo TauRD, *blue*; hTissue interactomes, *orange*). sTurbo TauRD uniquely captures hub proteins across modules, including previously validated SAFB, MACROH2A1, CHD4, as well as proteins CHERP, ILF3, SF3B3, SMU1, and BUB3. Shared RBPs across Tau interactome datasets include hnRNPs (HNRNPK, HNRNPAU, HNRNPA2B1, HNRNPM) and snRNPs (SNRNP70, SNRPD2, SNRPD3). RBP, RNA-binding protein; TauRD, tau microtubule repeat domain; sTurbo, split TurboID; *MAPT,* microtubule-associated protein tau; AP-MS, antibody-based affinity purification coupled to mass spectrometry.

To better understand the intersection and potential disease relevance between the detergent-insoluble proteome in tauopathies and tau-interacting proteins, we visualized a stacked bar chart of module members within the highly enriched insoluble RBP modules M18 and M5 from the TauRD interactome (Fig. 8*A*). Approximately, 50% of all M18 proteins mapped to the insoluble proteome (InsolAD, black; InsolCTL, white; sTurbo TauRD, blue; and hTissue, orange), TauRD interactors accounted for 11% of these insoluble-mapping module members (blue, gray) while human tau interactors were 3.92% (orange, grays). Similarly, 29.7% of M5 module members are insoluble with 7.5% represented in the TauRD interactome and 2.7% identified from hTissue interactome. sTurbo TauRD interactors were represented across both soluble and insoluble hub proteins in these selected modules (Fig. 8, *B–E*). Surprisingly, human AD tau-interacting proteins predominantly overlapped with insoluble proteins represented in M18 (Fig. 8*B*). Several module hub proteins intersected across TauRD and hTissue tau interactomes in M18 (SNRNP70, SNRPD2, HNRNPK, and TMPO) (Fig. 8*B*). SNRNP70, a previously identified tau interactor enriched across interactomes, and the previously validated TauRD interactor, SAFB (14) followed Braak staging (Fig. 8*C*). Meanwhile, HNRNPA3 (16), a hTissue tau interactor, protein levels did not correspond to tau deposition. CHERP, a spliceosome A-complex member uniquely identified in TauRD proteomics, was shown to be insoluble in AD but only displayed a modest, insignificant increase in protein levels and Braak stage (Fig. 8*C*). M5 module hub proteins included insoluble mapping SNRPD3, HNRNPU, HNRNPA2B1, HNRNPM, and PRPF8 (Fig. 8*D*). Protein abundances of the shared interactor, HNRNPU, and hTissue unique interactor, HNRNPC, both significantly increased along with tau aggregation in brain. Additionally, newly identified tau interacting proteins, MACROH2A1 and CHD4, presented a stepwise increase in protein levels alongside Braak stages, displaying strong association with tau deposition in disease. In cases such as HNRNPA2 and CHERP, modest and insignificant association with Braak stages and protein levels may indicate interplay between tau deposition and additional disease-associated processes, as well as brain-region specific detection of tau in each tauopathy, *i.e.,* cortical in AD and temporal in PSP and ability to capture insoluble interacting proteins from human tissue IPs. Overall, RBPs enriched in disease-associated modules included the previously validated CHD4, MACROH2A1, SAFB, WDR82, and PAPOLA proteins, highlighting their potential relevance to tauopathies. Although these proteins have been associated with models of AD (112, 113, 114, 115, 116, 117), they have not previously been explicitly identified as tau interactors.

Given the significance of newly identified protein interactors to tau, we investigated interacting and insoluble proteins with two biological pathways enriched in M18 and M5 proteins: mRNA surveillance and spliceosome complex (Fig. S7). The mRNA surveillance system manages the integrity of mRNA by detecting and degrading aberrantly processed mRNAs, commonly through nonmediated decay (118, 119). The exon–junction complex and related components act as a dynamic bridge between spliceosome and mRNA surveillance pathways (120). Of note, tau interacting proteins from human (PNN, RNPS1) and those uniquely detected in the TauRD interactome (MAGOH, UAP56) are proteins both comprising the exon–junction complex (Fig. S7*A*, black box). Several “transiently interacting factors” mediating RNA nuclear export, processing, and nonsense-mediated decay were identified in the TauRD interactome, in contrast to those found in hTissue studies. The spliceosome, which is comprised of several large protein complexes with accessory components, become catalytically active to excise intronic sequences within the mRNA in a highly regulated process (121). Tau has been shown to co-aggregate with several core spliceosome components in neurodegenerative diseases, including U1-70K (SNRNP70) and U1A (SNRPA) (16, 22, 70, 122). Interestingly, hnRNPs and SR-proteins, classes of RBP with versatile functionality across splicing and mRNA-processing pathways (123), were most consistent across all tau interactomes investigated (Fig. S7*B*).

Here, we demonstrate the utility of integrating sTurbo TauRD interacting proteins with a tauopathy proteomics network encompassing both AD and PSP cases to identify disease-relevant modules enriched with tau interactors. This analysis revealed several RBP modules that were enriched with TauRD-interacting proteins and positively associated with tau pathology that were likely missed in human tau interactome datasets due to their transient or insoluble nature in the human brain.

## Discussion

In this study, we demonstrate that the *in vitro* TauRD interactome identifies conserved cellular machinery across 2 cell types (HEK293 and SH-SY5Y) associated with cytoskeletal dynamics, stress response, and mRNA processing. By integrating additional human brain tau interactome studies and bulk brain proteomics, we show that the TauRD interactome provides shared insight into PPI changes, further reinforcing the role of RBPs in AD and related tauopathies (17, 20, 21, 24, 25, 29, 31, 32, 33, 63, 107, 124, 125). We uncovered new tau interacting proteins by comparing TauRD proteins from bulk frontal cortex insoluble proteomes. These protein abundances also increase with Braak staging, complementing previous work demonstrating RBPs aggregate alongside neuropathological tau burden (63). The TauRD interacting proteins within disease-related protein modules were enriched in nuclear pathways consistent with prior studies highlighting the relevance of splicing factors and RBPs in AD pathogenesis (16, 126) and loss of function with downstream splicing defects (16, 17, 22, 28, 31, 63, 108). Notably, the aggregation of these RBPs in AD also follows tau burden by Braak staging (63), suggesting a potential connection between pathological tau spread and the RBP and splicing dysregulation observed in tauopathies.

Previous work by our group and others has described core spliceosome members, including U1-70K (snRNP70) (22, 33, 122, 126, 127), and disordered RBPs such as G3BP1, TIA1, and SRRM2 in tau aggregation (25, 26, 128), co-aggregating with tau in the cytoplasm across cellular (25), murine models (21, 73) and in human AD brain tissue (22, 23, 122). Most RBPs lack a strong secondary and tertiary structure and are characterized as intrinsically disordered, enabling electrostatic interactions with other proteins and diverse mRNA species to carry out their homeostatic function (129). Notably, tau itself is highly disordered, facilitating its liquid–liquid phase separation into membraneless organelles such as stress granules or processing bodies to accelerate the fibrillization (81, 130, 131). These TauRD interactome results further support the role of tau and associated stress granule proteins.

Current research on tau aggregation is limited to understanding additional protein interactions impacting the oligomerization and, furthermore, aggregation of tau. To address this, we sought to develop a PL system using the domain of tau detected in paired helical filaments (9, 10, 71, 132, 133, 134). Dissecting tau interactors through immune affinity-capture is limited as it must be performed under lysis conditions preserving physiological interactions, whereas bulk cortical proteome was generated from tissues lysed in 8M urea, which can solubilize aggregated proteins. Tau interactome studies have primarily employed IP with tau Abs (13, 15, 16, 79, 100, 135, 136, 137), as well as capturing tau interactome by ascorbate peroxidase PL (14, 36). TurboID has the sensitivity to capture both transient and weakly interacting proteins, with labeling of Lys residues in a 10 nm range within 5 minutes (40, 41). TurboID is also more likely to favor labeling intrinsically disordered proteins, such as RBPs and tau itself, as the hydrophilic nature of Lys residues may allow peptides to be readily labeled (138). This study identified several unique RBPs with an average of ∼10% K residues. Many of these TauRD interactors were found to be disease-relevant in the bulk cortex of AD and PSP across both soluble and insoluble co-aggregating proteins. Interestingly, tau IP interactomes overlapped with some insoluble-mapping RBPs across M18 and M5. We hypothesize these were captured from a soluble pool of protein (22).

sTurbo TauRD surprisingly labeled many proteins that did not overlap with the tau interactome datasets nor were reported by other tau interactomes (13, 14, 79, 100, 135, 136, 139). Specifically, we highlight the AD insoluble-mapped proteins CHERP, CHD4, WDR82, and SNRNP200, as well as enriched proteins not mapping to AD insoluble fractions: SAFB and MACROH2A1. These findings may be influenced by several factors, including the limitations of conventional IP methods in capturing these interactions, and differences in the PL technique implemented. Overall, the sTurbo TauRD *in vitro* interactome complements prior tau interactome studies outlining the physiological involvement of tau with proteasomal subunits (15, 79), DNA-binding proteins (15), 14-3-3 binding proteins (13, 15, 79, 100, 135), and vesicle sorting/trafficking (14, 139) while identifying new protein interactors relevant to module of disease-associated proteins, displaying consistency to previously established disease-relevant tau PPIs networks. These distinct interactors provide additional avenues for future studies to evaluate mechanistic involvement of tau fibrillization in the spliceosome, nascent mRNA processing, and nonsense mediated decay.

As a monoculture method, this study did not identify brain cell type–specific differences in tau aggregation biology nor cell-to-cell dynamic crosstalk. Future studies implementing adenovirus and transgenic models may be incorporated to deliver sTurbo TauRD in a cell-type–specific manner to observe the dynamic interplay of neural cells (34, 140). Overexpression of the TauRD may bias cells toward a stress state, especially as tau is not endogenously expressed in HEK-293 cells and would not have native interactors seen in differentiated neuronal lines. Although, incorporation of SH-SY5Y in this study orthogonally detected unique and common RBPs and mRNA processing proteins (SNRNP70, YTHD3, CSDE1, and HNRNPA1L2). Additionally, MTBR inclusions seen in this model are consistent with prior studies showing stress response or autophagosome overlap (30, 136, 141). Further discordance of sTurbo TauRD results in comparison to other tau interactome datasets may be due to various reasons, including mutation differences (P301L, V337M, and WT) influencing interactions and distinction from the tau domain (full-length, 3R MTBR isoform, N-terminal region, Proline-rich domain, and C-terminal region) used in this study. Finally, we sought to build upon the previously characterized HEK-FRET TauRD biosensor aggregation cell line and extract the same amino acids of the MTBR (L243 to K375) for this model. However, further studies have demonstrated that while most of the MTBR is represented in tangles, it is not the whole fibril core (142). Paired helical filament fibrils are composed of amino acids V336 to F378 (142). The difference in these 10 amino acids may alter this domain's structural organization and biochemical interactions *in vitro* and may account for nuanced differences in interactors. Additional to this, studies have described that the structure of the TauRD with larger appendages, such as the split GFP, impact the fibril structure and aggregation propensity of the TauRD (143). While this steric clash has not been directly studied in relation to split biotin ligase, our results describe a similar aggregation profile and expression localization to FRET TauRD. Fibril structure, and subsequent PPIs of the TauRD, are sensitive to local cellular environment and post-translational modifications, which differs across cellular models from the brain milieu. Therefore, this present study utilizing cell models is inherently limited in directly identifying interactors of brain-specific TauRD. Despite these clear limitations, this study contributes to our understanding of disease-relevant protein–protein interactors in tauopathies and provides new candidate RBPs associated with tau pathology in disease (144).

Ultimately, the sTurbo TauRD system acts as a platform for the protein aggregation field to characterize both soluble and insoluble PPI networks related to human disease. These data also indicate that this tau PL approach provides insight into unique nuclear pathways, highlighting the importance of RBPs and spliceosome members for future mechanistic studies and potential therapeutic targets in disease.

## Data Availability

The mass spectrometry proteomics RAW files and search results have been deposited to the ProteomeXchange Consortium *via* the PRIDE partner repository with the dataset identifier PXD059716 and PXD066874. RAW files and FragPipe output directories from HEK293 sTurbo TauRD DDA proteomics are also available on https://www.synapse.org syn64396764. The 2024 human Uniprot database containing 20,597 reviewed proteins (downloaded February 7th, 2024, from https://www.uniprot.org/) was used for searches of mass spectrometry data. Source data are provided with this paper. Annotated spectra of DDA single identification peptides are provided as Figure S8.

## Supplemental Data

This article contains supplemental data (23, 48, 82, 116, 139, 140, 141, 142, 143, 144).

## Conflict of Interest

N. T. S. and D. M. D. are co-founders of Emtherapro and Arc Proteomics. N. T. S. is co-founder of Stitch Rx.
