# Supplementary figures and images for: Proximity Labeling of the Tau Repeat Domain Enriches RNA-Binding Proteins That Are Altered in Alzheimer's Disease and Related Tauopathies
Source: Mol Cell Proteomics. 2025 Nov 7;25(1):101458. doi: 10.1016/j.mcpro.2025.101458 (PMC12796112; doi:10.1016/j.mcpro.2025.101458)

## Supplemental Figure 1

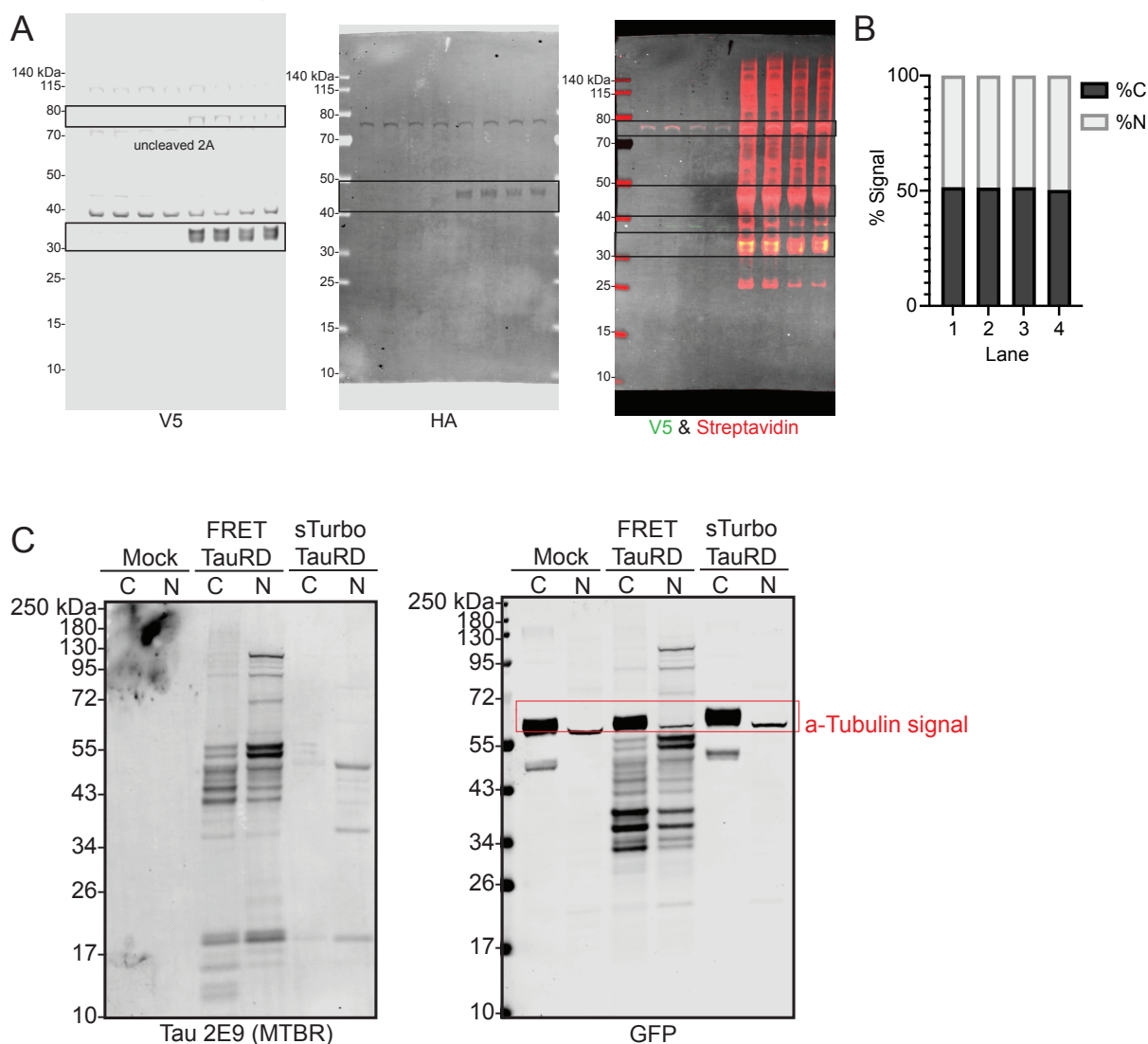

Supplement: Figure S1 [file mmc1.pdf]
