# Supplementary material for: Proximity Labeling of the Tau Repeat Domain Enriches RNA-Binding Proteins That Are Altered in Alzheimer's Disease and Related Tauopathies
Source: Mol Cell Proteomics. 2025 Nov 7;25(1):101458. doi: 10.1016/j.mcpro.2025.101458 (PMC12796112; doi:10.1016/j.mcpro.2025.101458)
Supplement: Figure S2 [file mmc2.pdf]

Supplemental Figure 2

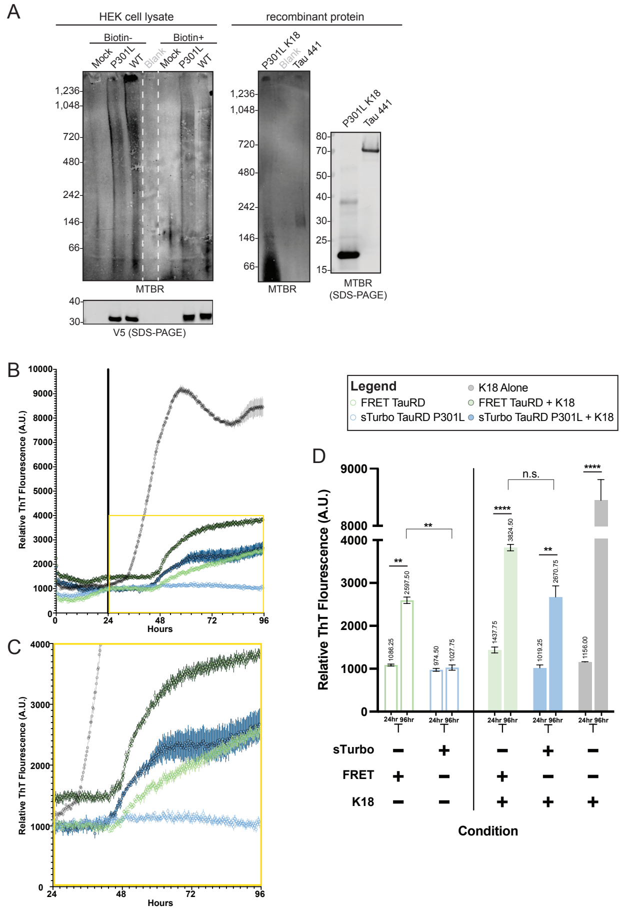

Supplemental Figure S2. Biochemical characterization of sTurbo TauRD aggregation state in HEK293 cells. (A) Western blot of Blue-Native PAGE HEK293 cells transfected sTurbo TauRD with or without the pro-aggregation P301L mutation, detected. The split Turbo appendages do not impede aggregation ability of the MTBR in either condition. Additionally, high molecular weight species formation is not impeded by biotin supplementation. V5 banding confirms recombinant protein expression in sTurbo TauRD conditions. Recombinant isolated tau four repeat domain (K18) with the P301L mutation displays similar high molecular weight streaking under native conditions and multimerization in denaturing electrophoresis (bands observed at ~20, ~40, and ~60 kDa) whereas the full-length protein (Tau 441) does not display a similar aggregation profile in either BN-PAGE or SDS-PAGE. **(B-D)** Thioflavin T (ThT) aggregation microplate assay displays increased ThT signal over 96 hours. **(B)** ThT fluorescence output as a function of time of HEK293 cell lysates transfected with either FRET TauRD (green) or sTurbo TauRD (blue) both without a tau seed and with a tau seed (+K18 protein fragment). Equal concentrations of K18 (5 μg/μL) and lysates (5 μg/μL) were loaded per assay in quadruplicate. The K18 monomer (25 μM) was included as a positive control to delineate the transition of the nucleation phase to elongation phase of the isolated TauRD. Average ThT (20 μM, n=8) signal was subtracted from individual experimental replicates before visualization. **(C)** Inset of ThT fluorescence from 24 hr time point to final read, before K18 monomer elongation phase as quantified in (D). A sigmoidal curve shown in isolated K18, FRET + K18, and sTurbo + K18, is consistent with fibrillation kinetics. Average across technical replicates (n=4) and error bars representing SEM are visualized. **(D)** Quantification of relative ThT intensities of 24 hr vs 96 hr time points across HEK293 lysates containing either sTurbo TauRD or FRET TauRD. K18 fragment was incorporated as a seed (+K18) and as an isolated positive aggregation control. FRET TauRD cell lysate display increased ThT signal without a tau seed between the first 24 hours and final plate read (96 hour). All TauRD cell lysates displayed an increase in beta-sheet formation via increased ThT fluorescence emission over time with the addition of K18 tau seed. Average across technical replicates (n=4) and error bars as SEM are plotted. Statistical analysis was determined by two-way repeated measures ANOVA with multiple comparisons of 24 hr and final read where a p-value < 0.05 and 95% confidence interval was considered significant. The main effect of condition was significant, $F(4, 15) = 38.47$, $p<0.0001$. Both Condition, $F(4, 15) = 50.74$, $p<0.0001$, and Time, $F(1,15) = 167.2$, $p<0.0001$ had effects on experimental outcome while replicate effects, $F(15,15)$, were not significant ($P=0.6372$). Two-way ANOVA Tukey multiple comparisons for pairwise testing are visualized (*, $p<0.05$; **, $p<0.01$; ***, $p<0.001$, ****, $p<0.0001$).
