# Supplementary material for: Proximity Labeling of the Tau Repeat Domain Enriches RNA-Binding Proteins That Are Altered in Alzheimer's Disease and Related Tauopathies
Source: Mol Cell Proteomics. 2025 Nov 7;25(1):101458. doi: 10.1016/j.mcpro.2025.101458 (PMC12796112; doi:10.1016/j.mcpro.2025.101458)
Supplement: Figure S3 [file mmc3.pdf]

Supplemental Figure 3

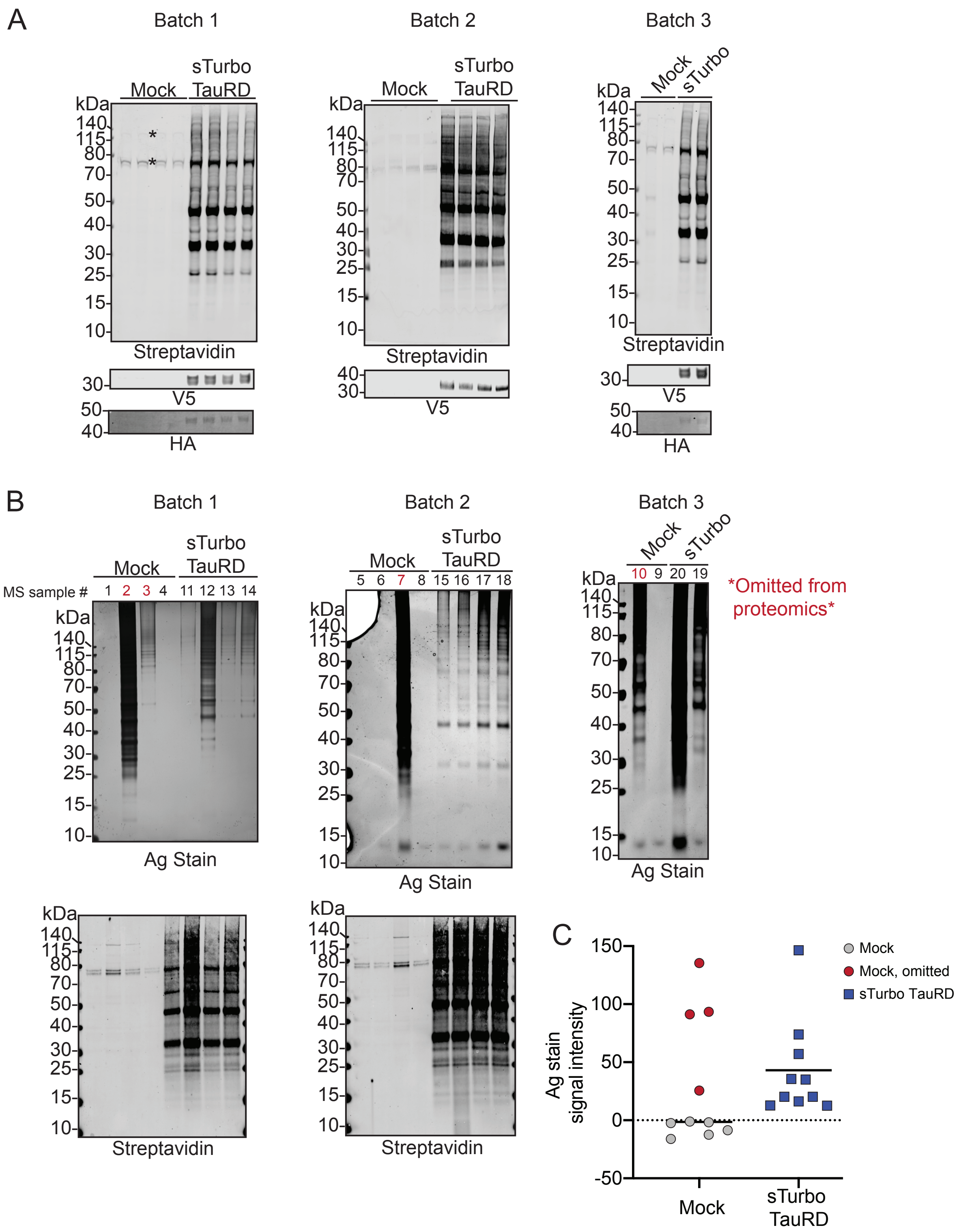

Supplemental Figure S3. **Quality control western blots and total protein silver stains. (A)** Total lysate input (20 µg) of sTurbo TauRD from HEK293 cells shows robust and consistent labeling across replicates and batches, shown by streptavidin blots. V5 and HA recombinant protein tags were also probed to confirm expression of sTurbo TauRD across lysates. **(B)** Silver stain (Ag stain) displaying total protein after streptavidin affinity purification shows varying total signal in sTurbo TauRD samples and has enriched biotinylated proteins, shown through streptavidin blot. Mock samples with non-specific binding to streptavidin beads, and resulting positive signal found in silver stain, were omitted from this proteomics study (red). Signal intensities from the Ag stain gel lanes are depicted in panel **(C).**
