# Supplementary material for: Proximity Labeling of the Tau Repeat Domain Enriches RNA-Binding Proteins That Are Altered in Alzheimer's Disease and Related Tauopathies
Source: Mol Cell Proteomics. 2025 Nov 7;25(1):101458. doi: 10.1016/j.mcpro.2025.101458 (PMC12796112; doi:10.1016/j.mcpro.2025.101458)
Supplement: Figure S4 [file mmc4.pdf]

Supplemental Figure 4

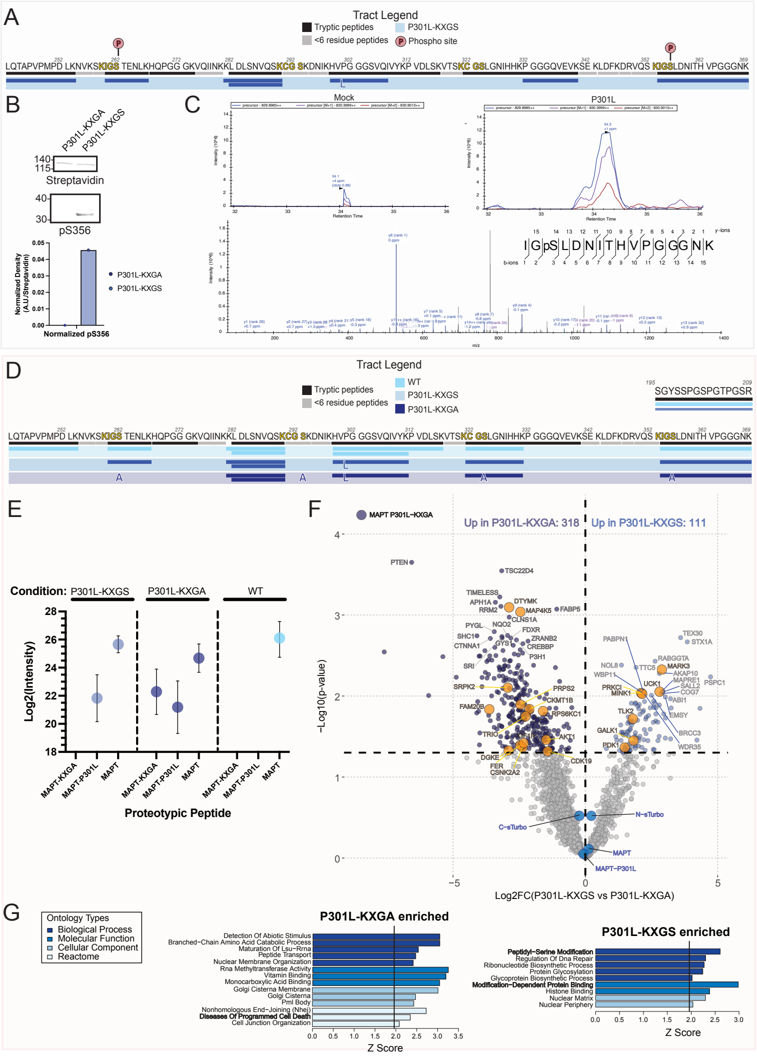

Supplemental Figure S4. Phosphorylation mapping of the TauRD in HEK293 cells and functional interactome of phospho-null mutant. (A) Linear sequence of tau peptides in sTurbo TauRD P301L HEK293 lysates in black alongside phosphorylation sites (red), namely in the KXGS motifs (yellow), identified by spectral search incorporating +79.97 Da mass shift on S/T/Y residues. **(B)** Western blot characterization of P301L-KXGS confirming a reduction in phosphorylation due to S → A mutation at residue S356. Endogenously biotinylated protein band at ~135 kDa in streptavidin blots was used as loading control across lanes. Phosphorylated S356 signal was normalized to streptavidin band intensity and visualized. **(C)** Peak selection outlining MS1 precursors to the modified "IGpSLDNITHVPGGGNK" peptide in Mock (left) and P301L-KXGS (right) conditions. MS2 annotated spectra denote well-characterized products (b and y ions) only in the P301L-KXGS experimental condition. **(D)** Linear sequence of tau peptides and colored tracts indicating mapped peptides across wild-type (WT), P301L-KXGS, and P301L-KXGA conditions. Introduced mutations (P301L, S → A at S262, S293, S324, and S356 are also noted. **(E)** Log2Intensity values of unique sequences mapping to MAPT-KXGA, MAPT-P301L, or MAPT (total) across P301L-KXGS, P301L-KXGA, and WT conditions. Mapped peptides confirm inclusion of P301L mutation in P301L and KXGA conditions, but not WT sTurbo TauRD. Meanwhile, the KXGA condition also includes KXGA peptide match. **(F)** Volcano plot displays Log2 Fold Change between TurboID-normalized P301L-KXGS and P301L-KXGA groups plotted on the x-axis while -Log10 unadjusted p-value, as determined by one-way ANOVA, is plotted on the y-axis. A p < 0.05 (-Log10 = 1.3) was employed to identify significantly enriched proteins. Dark blue points indicate proteins significantly enriched in the P301L-KXGA condition (n=318), while blue points indicate proteins enriched in the P301L condition (n=111). Kinases are labeled in orange. sTurbo fragments (N and C) and MAPT proteins groups (KXGA, P301L, total) are also labeled. As expected, KXGA-MAPT is most differentially abundant in the P301L-KXGA condition whereas P301L-MAPT levels were normalized across groups. **(G)** Top Gene Ontology analysis of differentially enriched proteins in the P301L-KXGA condition describes apoptotic and cellular stress response pathways. GO analysis of P301L-KXGS pulldown confirms post-translational modification and nuclear pathways.
