# Supplementary material for: Proximity Labeling of the Tau Repeat Domain Enriches RNA-Binding Proteins That Are Altered in Alzheimer's Disease and Related Tauopathies
Source: Mol Cell Proteomics. 2025 Nov 7;25(1):101458. doi: 10.1016/j.mcpro.2025.101458 (PMC12796112; doi:10.1016/j.mcpro.2025.101458)
Supplement: Figure S5 [file mmc5.pdf]

Supplemental Figure 5

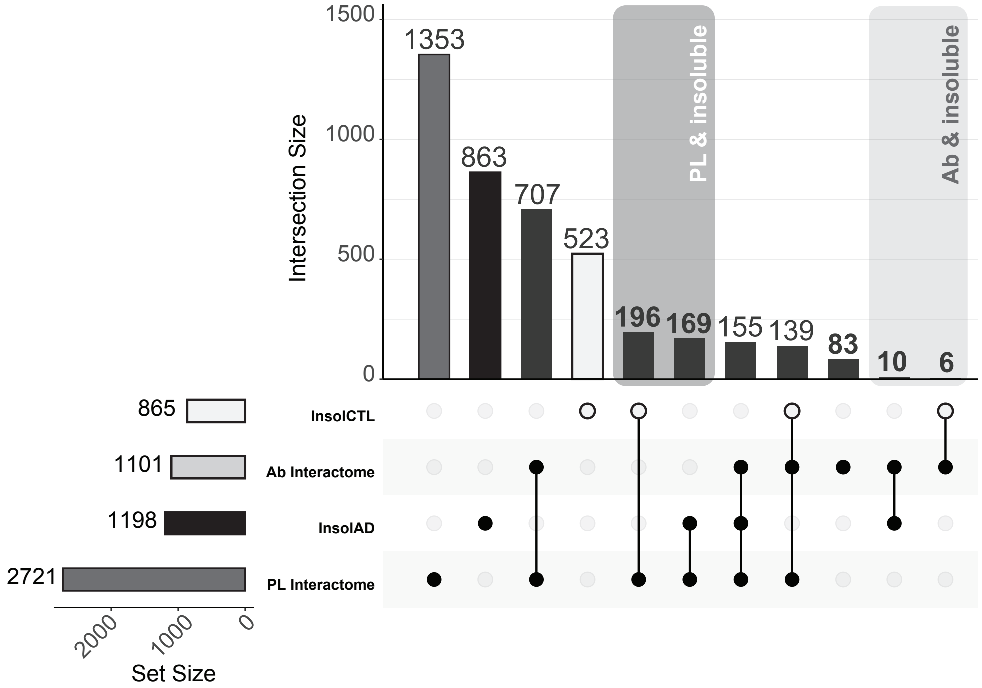

Supplemental Figure S5. **Tau proximity labeling captures more insoluble proteins than tau antibody-based affinity purifications.** Proteins found in tau interactomes acquired via antibody-based affinity purification studies (Ab Interactome, n=7) approaches versus enzymatic proximity labeling (n=3) shows an increase in interacting proteins overlapping with the sarkosyl insoluble fraction of either non-demented control (InsolCTL) and AD brain (InsolAD) with 365 unique insoluble proteins identified in proximity labeling experiments and only 16 identified from antibody studies.
