# Supplementary material for: Proximity Labeling of the Tau Repeat Domain Enriches RNA-Binding Proteins That Are Altered in Alzheimer's Disease and Related Tauopathies
Source: Mol Cell Proteomics. 2025 Nov 7;25(1):101458. doi: 10.1016/j.mcpro.2025.101458 (PMC12796112; doi:10.1016/j.mcpro.2025.101458)
Supplement: Figure S6 [file mmc6.pdf]

Supplemental Figure 6

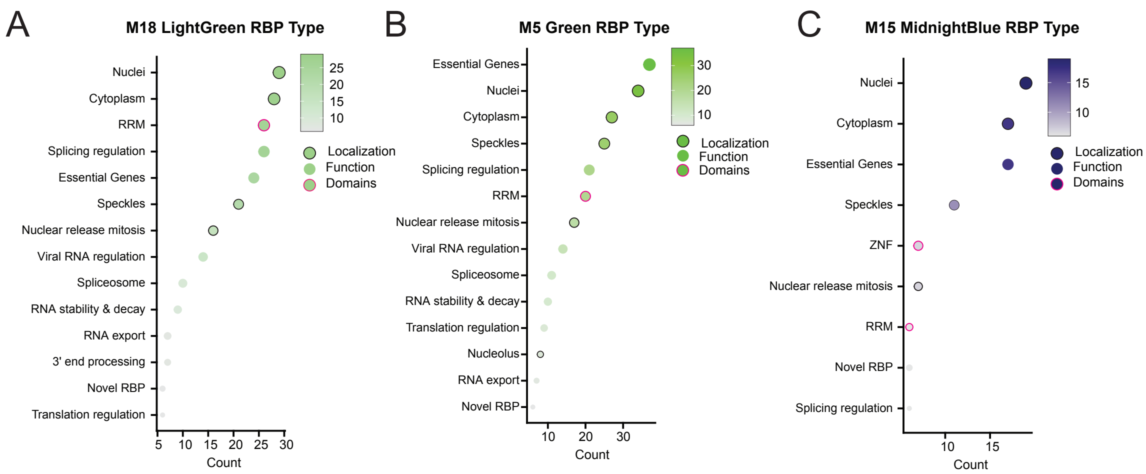

Supplemental Figure S6. Insoluble disease mapping module classification across RBP localization, key functions, and domains. Module members across RBP-enriched modules. M18 **(A),** M5 **(B)**, and M15 **(C)** were integrated with a list of 356 well-characterized RBPs to delineate common and divergent features across each module. Count of module RBPs mapping to each characteristic is visualized.
