# Supplementary material for: Proximity Labeling of the Tau Repeat Domain Enriches RNA-Binding Proteins That Are Altered in Alzheimer's Disease and Related Tauopathies
Source: Mol Cell Proteomics. 2025 Nov 7;25(1):101458. doi: 10.1016/j.mcpro.2025.101458 (PMC12796112; doi:10.1016/j.mcpro.2025.101458)
Supplement: Figure S7 [file mmc7.pdf]

Supplemental Figure 7

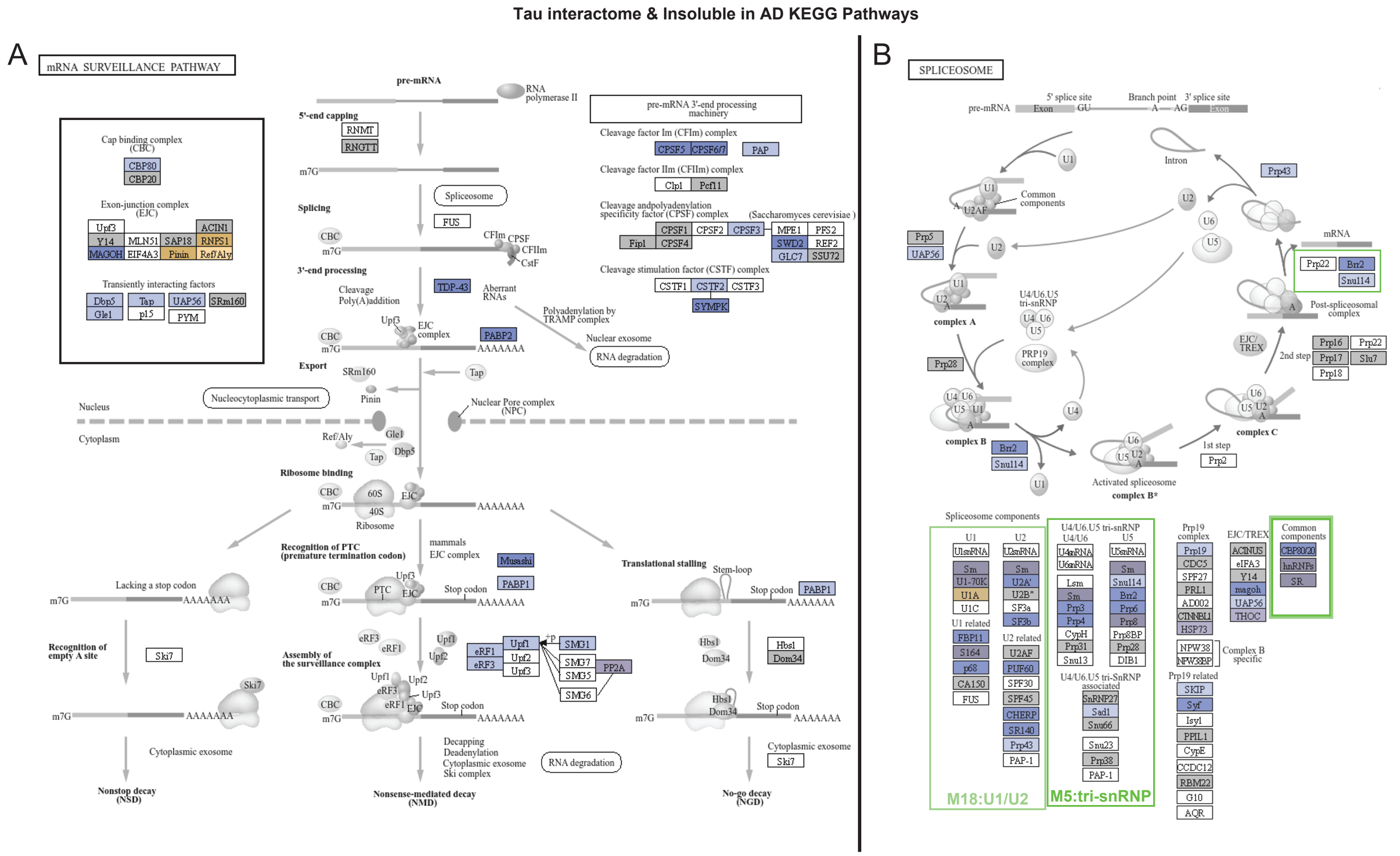

Supplemental Figure S7. Tau interactome members are enriched in KEGG biological pathways relating to mRNA surveillance (A) and spliceosome complex members (B). Pathways rendered with PathView. sTurbo TauRD interactors are largely related to mRNA quality control, providing context to the multifunctionality of newly identified tau interactors from this TauRD study as well as complementary processes identified from hTissue Tau interactomes. This pathway analysis also describes nuances in types of insoluble-mapping RBPs enriched in M18, such as U1/U2 snRNPs, versus M5 (U4/5/6 tri-snRNP). Colors of overlapping proteins align with legend found in Fig. 8 (sTurbo TauRD, blue; hTissue, orange; insoluble in AD, dark gray, overlaps of sTurbo TauRD and hTissue, purple).
