# Supplementary material for: Proximity Labeling of the Tau Repeat Domain Enriches RNA-Binding Proteins That Are Altered in Alzheimer's Disease and Related Tauopathies
Source: Mol Cell Proteomics. 2025 Nov 7;25(1):101458. doi: 10.1016/j.mcpro.2025.101458 (PMC12796112; doi:10.1016/j.mcpro.2025.101458)
Supplement: Figure S8 [file mmc8.pdf]

Supplemental Figure S8. Annotated spectra of one-hit peptide identifications

Peptides identified by 1 spectral hit in DDA HEK293 proteomics, and retained as individual protein groups for downstream analyses, were annotated with ProtViz adapted for FragPipe outputs. Best spectra scan ID was selected for annotation. Fragment ions (b & y) and intensities are visualized.

AIAANEADAVTLDAGLVYDAYLAPNNLK

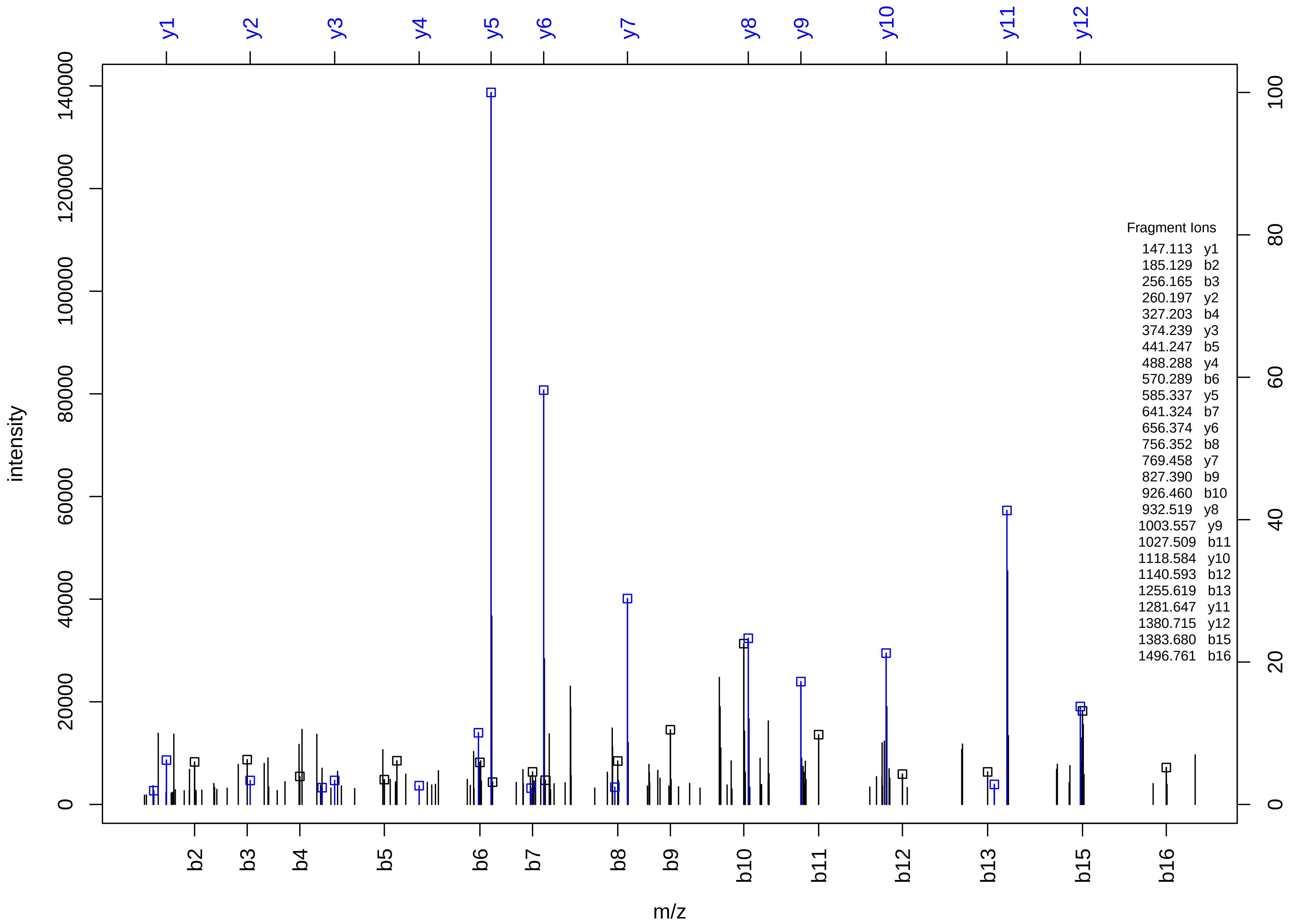

AIAANEADAVTLDAGLVYDAYLAPNNLK / File: sarah_20ip_13−P301L−1−3.raw; SpectrumID: ; scans: 54486

AINAALAQR

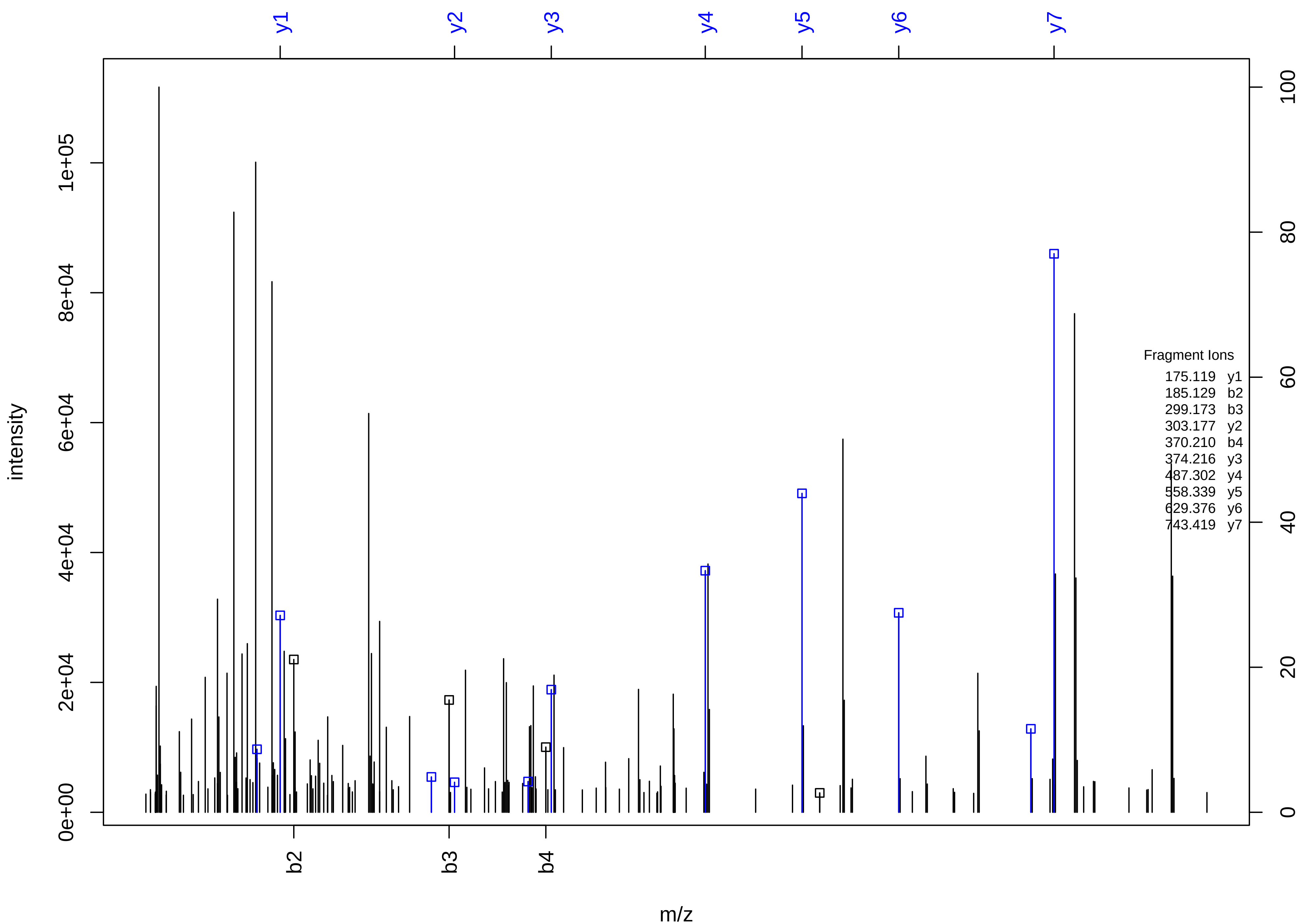

AINAALAQR / File: sarah_20ip_13−P301L−1−3.raw; SpectrumID: ; scans: 6491

ALC[57.0215]DVGTAISC[57.0215]SR

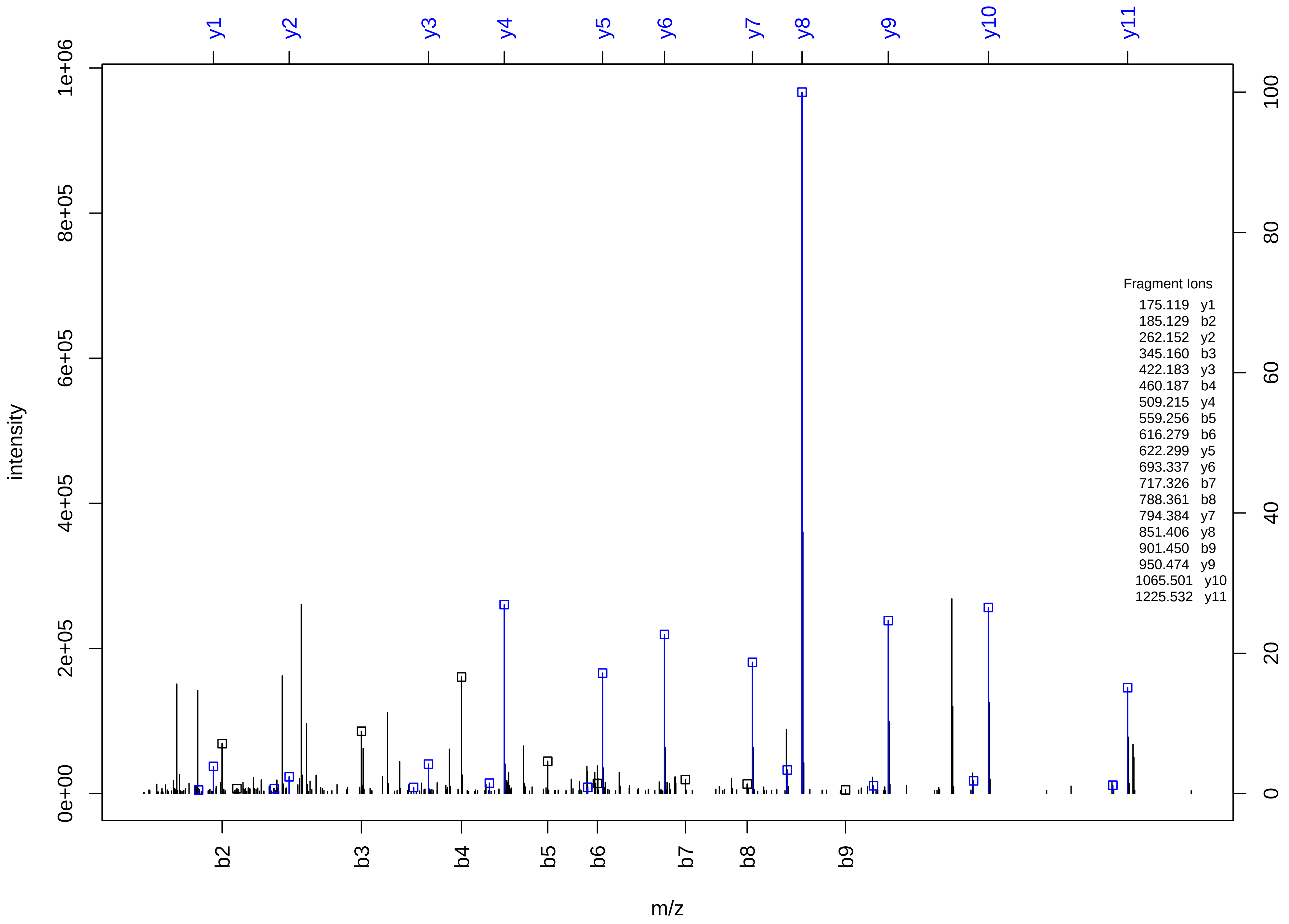

ALCDVGTAISCSR / File: sarah_20ip_12−P301L−1−2.raw; SpectrumID: ; scans: 18099

(Ac)ATFVSELEAAK

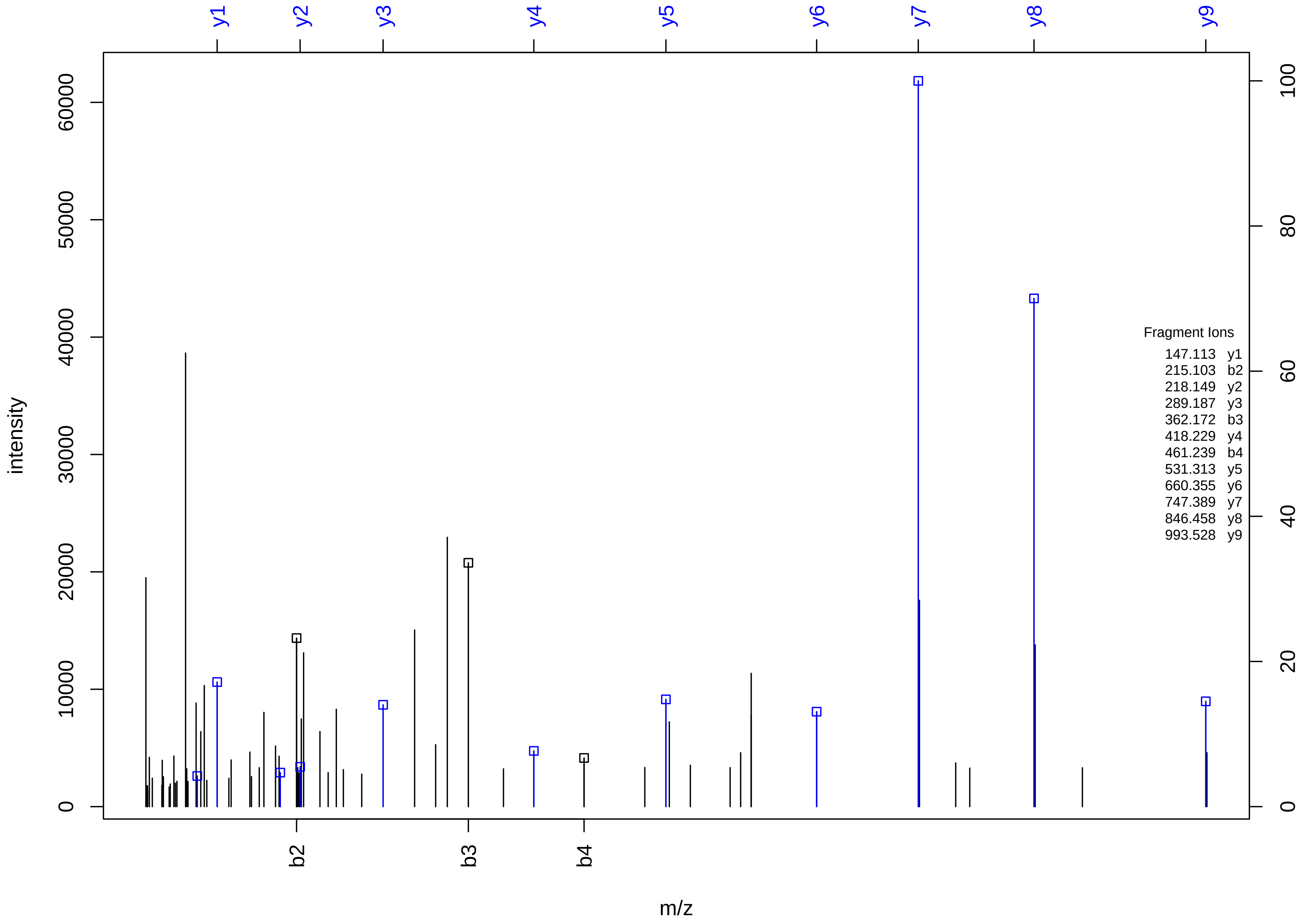

ATFVSELEAAK / File: sarah_20ip_13−P301L−1−3.raw; SpectrumID: ; scans: 49941

(Ac)ATPGPVIPEVPFEPSKPPVIEGLSPTVYR

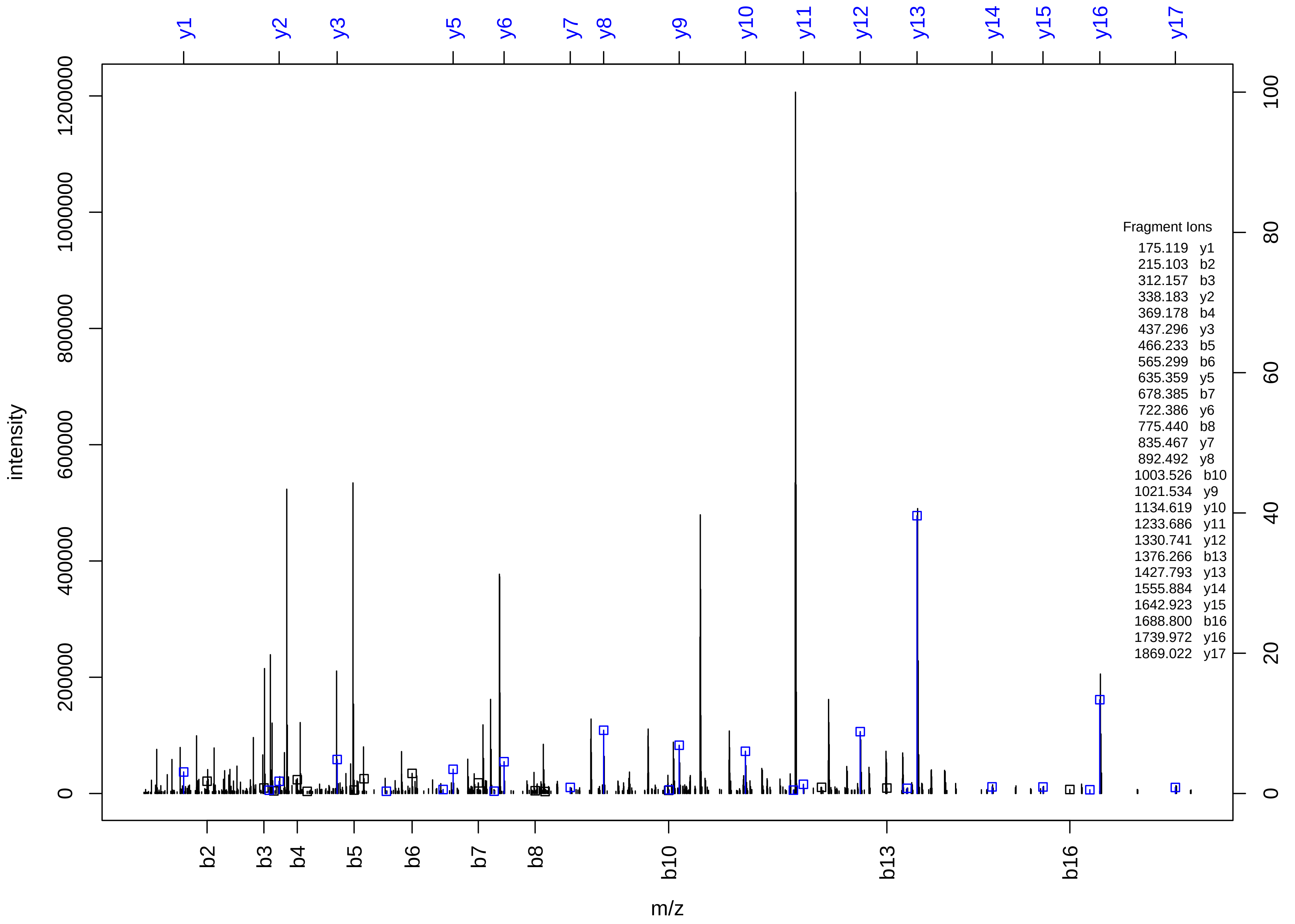

ATPGPVIPEVPFEPSKPPVIEGLSPTVYR / File: sarah_20ip_20−P301L−3−1.raw; SpectrumID: ; scans: 53258

EGWEYVK

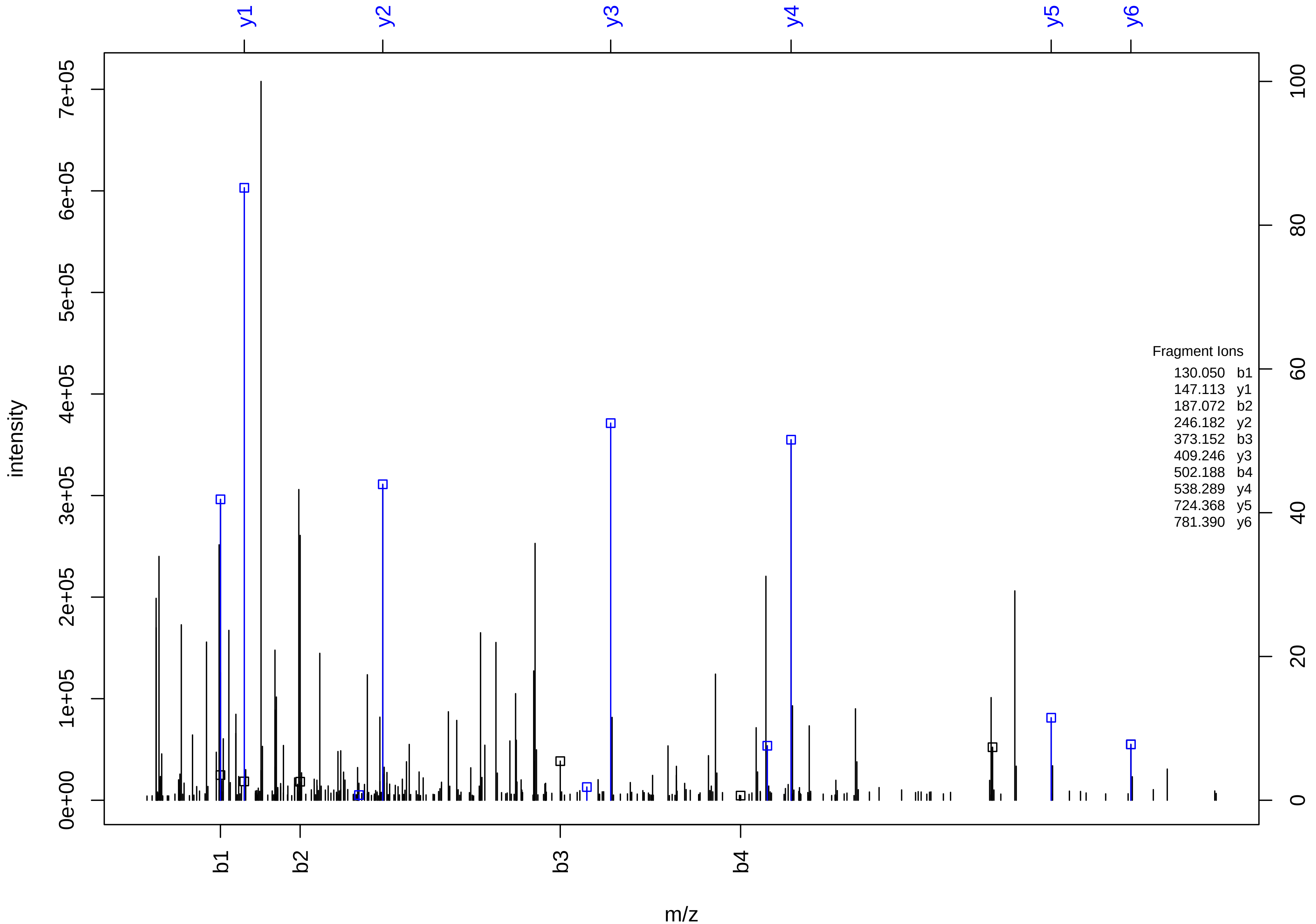

EGWEYVK / File: sarah_20ip_20−P301L−3−1.raw; SpectrumID: ; scans: 15043

ESTYQGHHTPPVQK

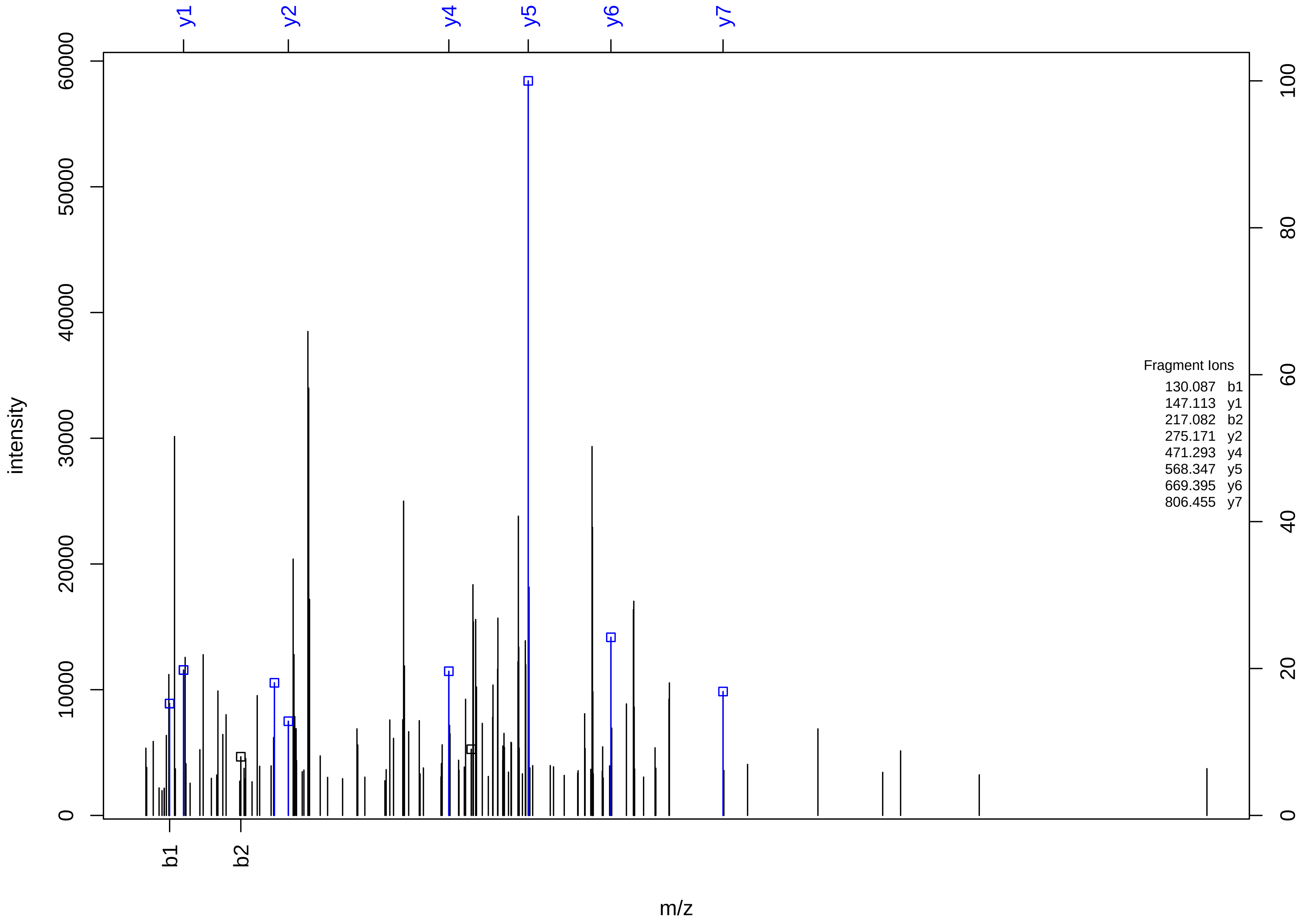

ESTYQGHHTPPVQK / File: sarah_20ip_13−P301L−1−3.raw; SpectrumID: ; scans: 1241

FVNVVPTFGK

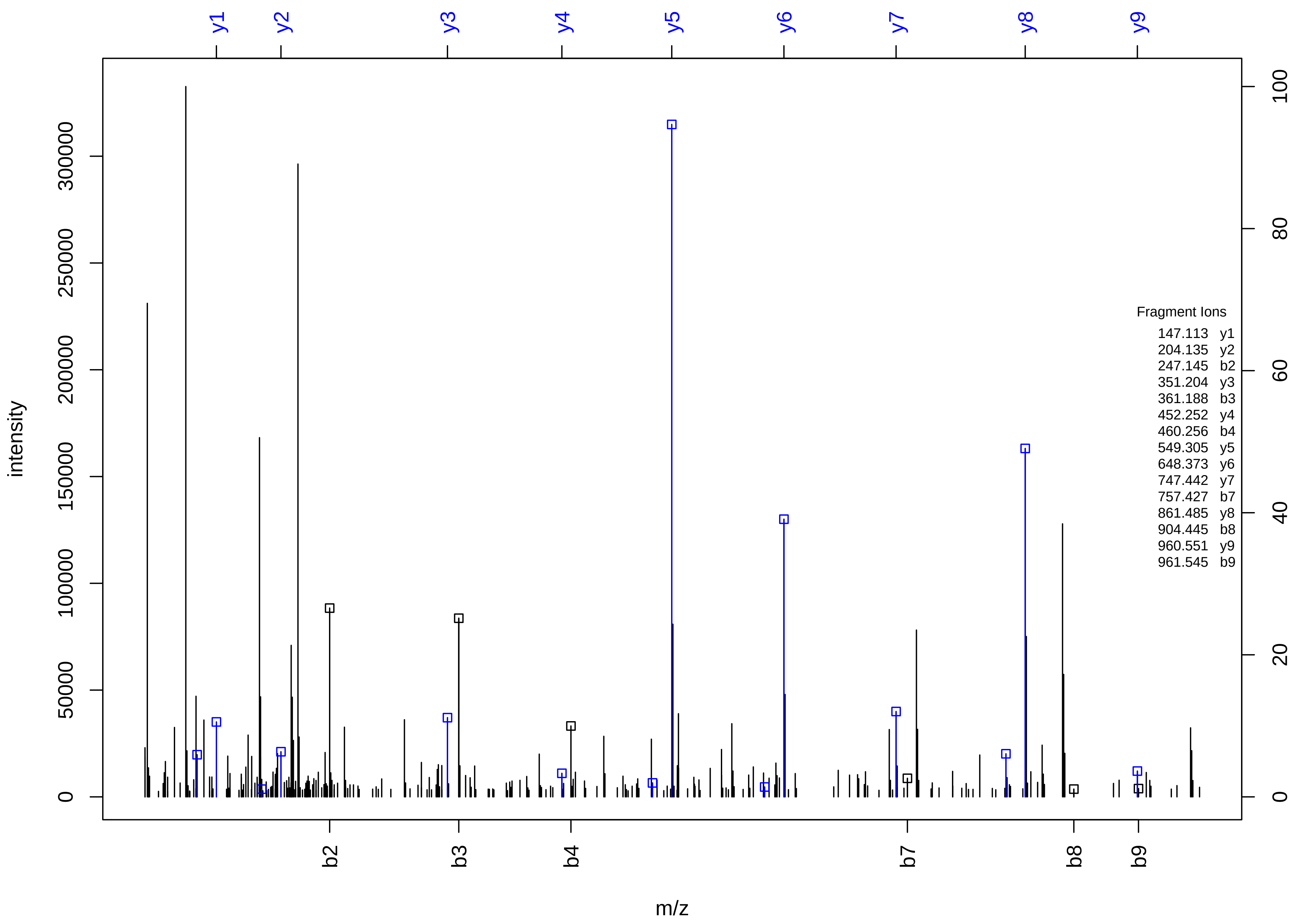

FVNVVPTFGK / File: sarah_20ip_12−P301L−1−2.raw; SpectrumID: ; scans: 31843

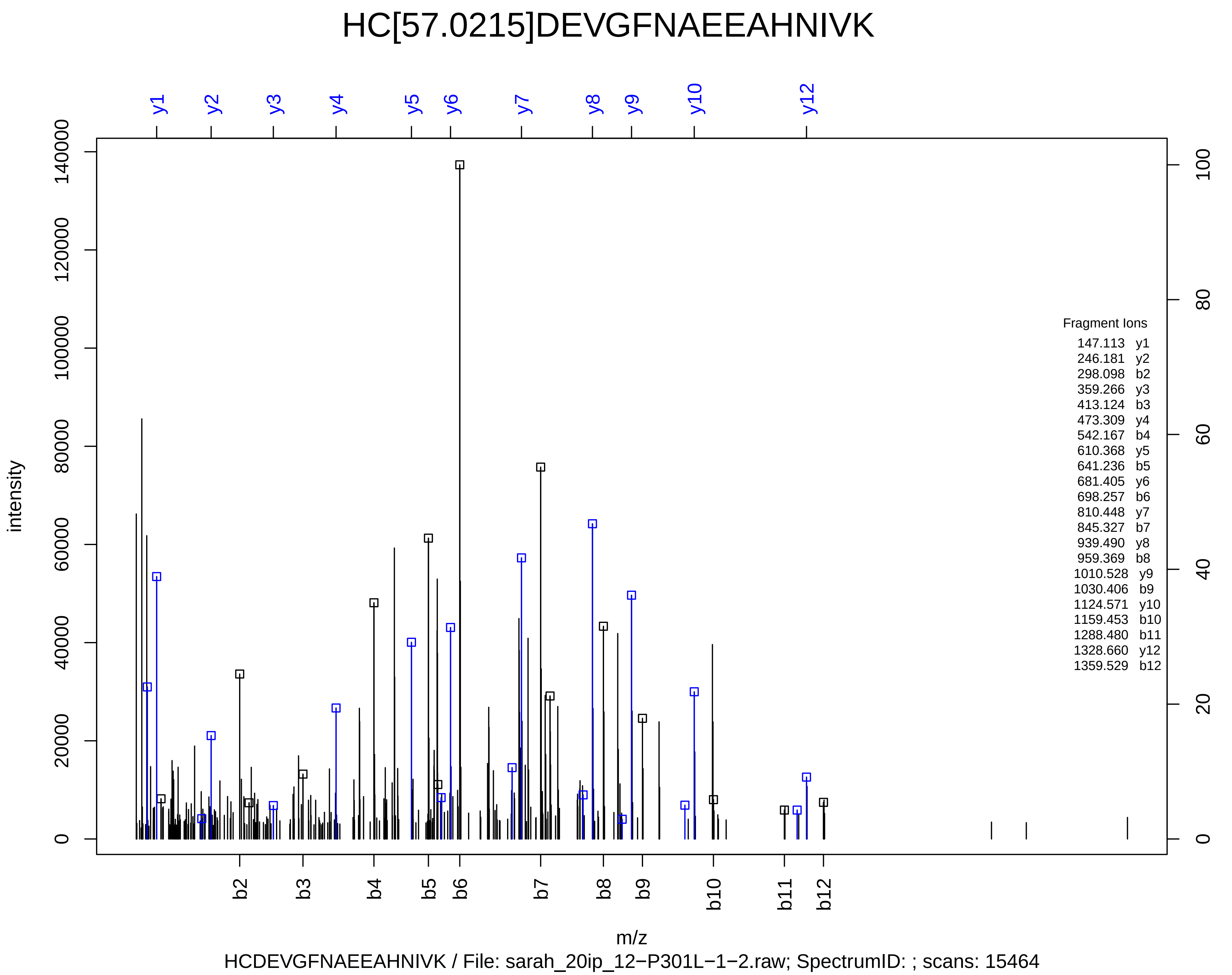
HC[57.0215]DEVGFNAEEAHNIVK
y1
y2
y3
y4
y5
y6
y7
y8
y9
y10
y12
intensity
0
20000
40000
60000
80000
100000
120000
140000
0
20
40
60
80
100
b2
b3
b4
b5
b6
b7
b8
b9
b10
b11
b12
m/z
Fragment Ions
147.113 y1
246.181 y2
298.098 b2
359.266 y3
413.124 b3
473.309 y4
542.167 b4
610.368 y5
641.236 b5
681.405 y6
698.257 b6
810.448 y7
845.327 b7
939.490 y8
959.369 b8
1010.528 y9
1030.406 b9
1124.571 y10
1159.453 b10
1288.480 b11
1328.660 y12
1359.529 b12
HCDEVGFNAEEAHNIVK / File: sarah_20ip_12−P301L−1−2.raw; SpectrumID: ; scans: 15464

ILAPLLILDK

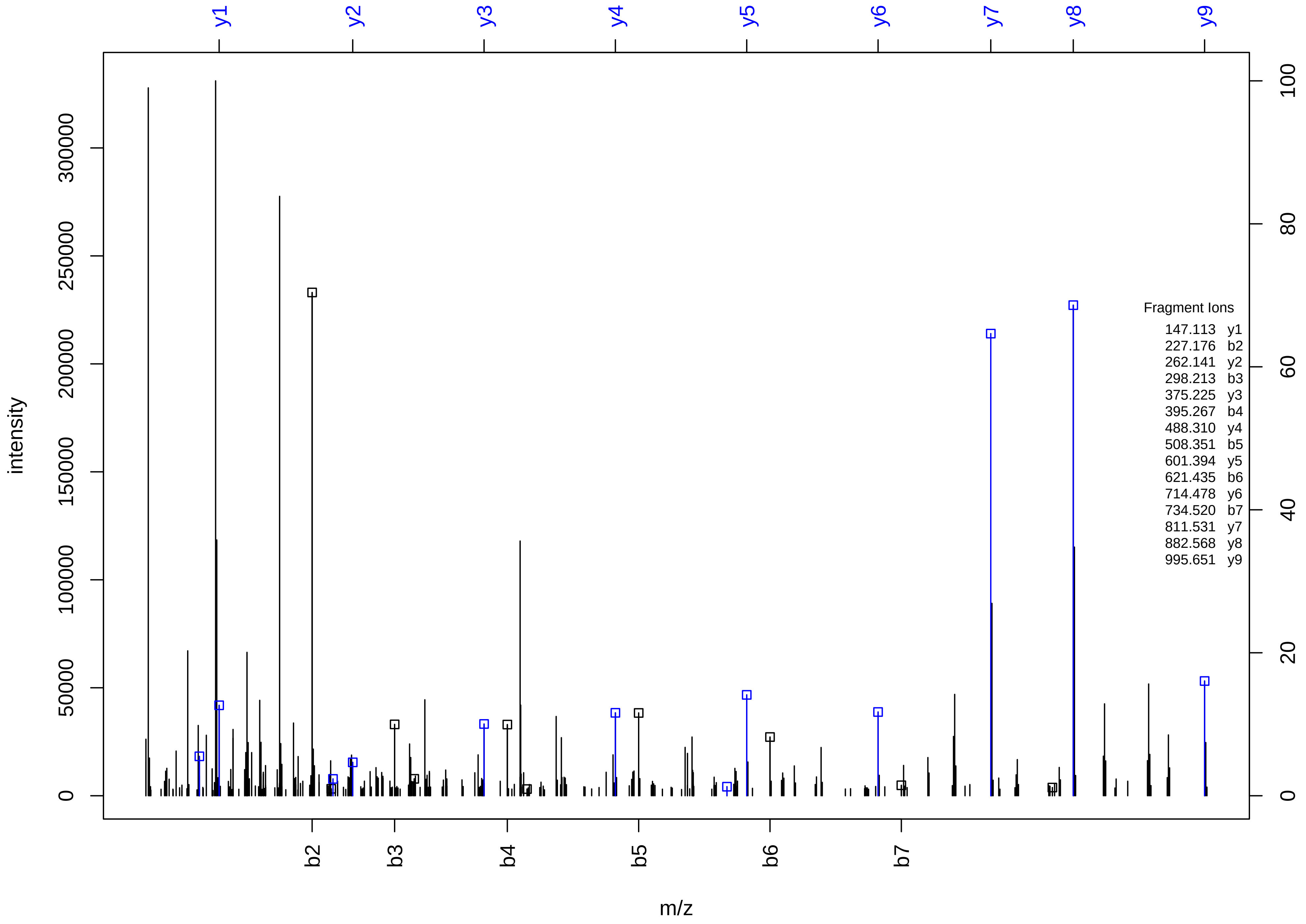

ILAPLLILDK / File: sarah_20ip_20−P301L−3−1.raw; SpectrumID: ; scans: 49848

ILLNPVQVFDVTK

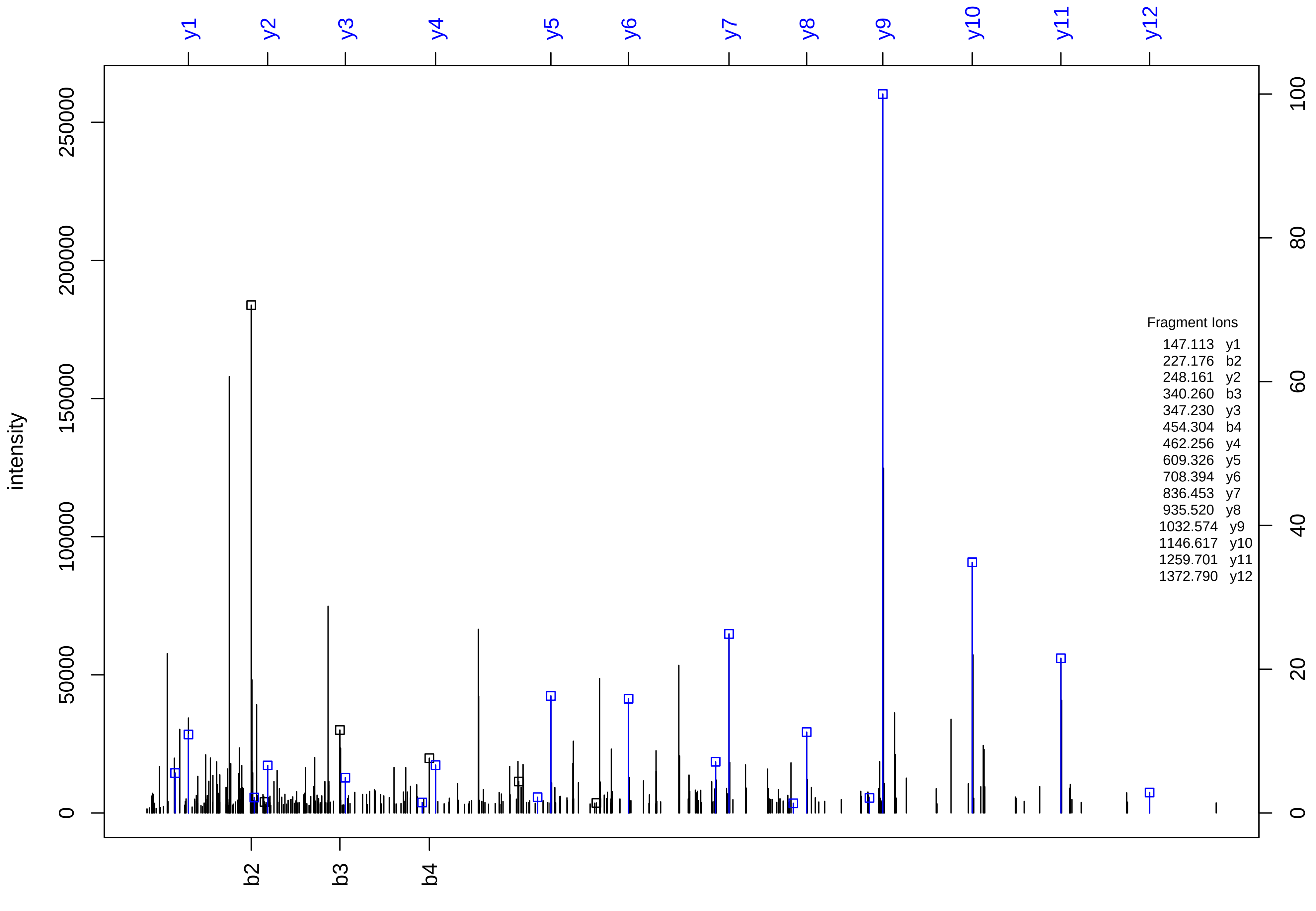

ILLNPVQVFDVTK / File: sarah_20ip_12−P301L−1−2.raw; SpectrumID: ; scans: 49054

LILDWVPYINGK

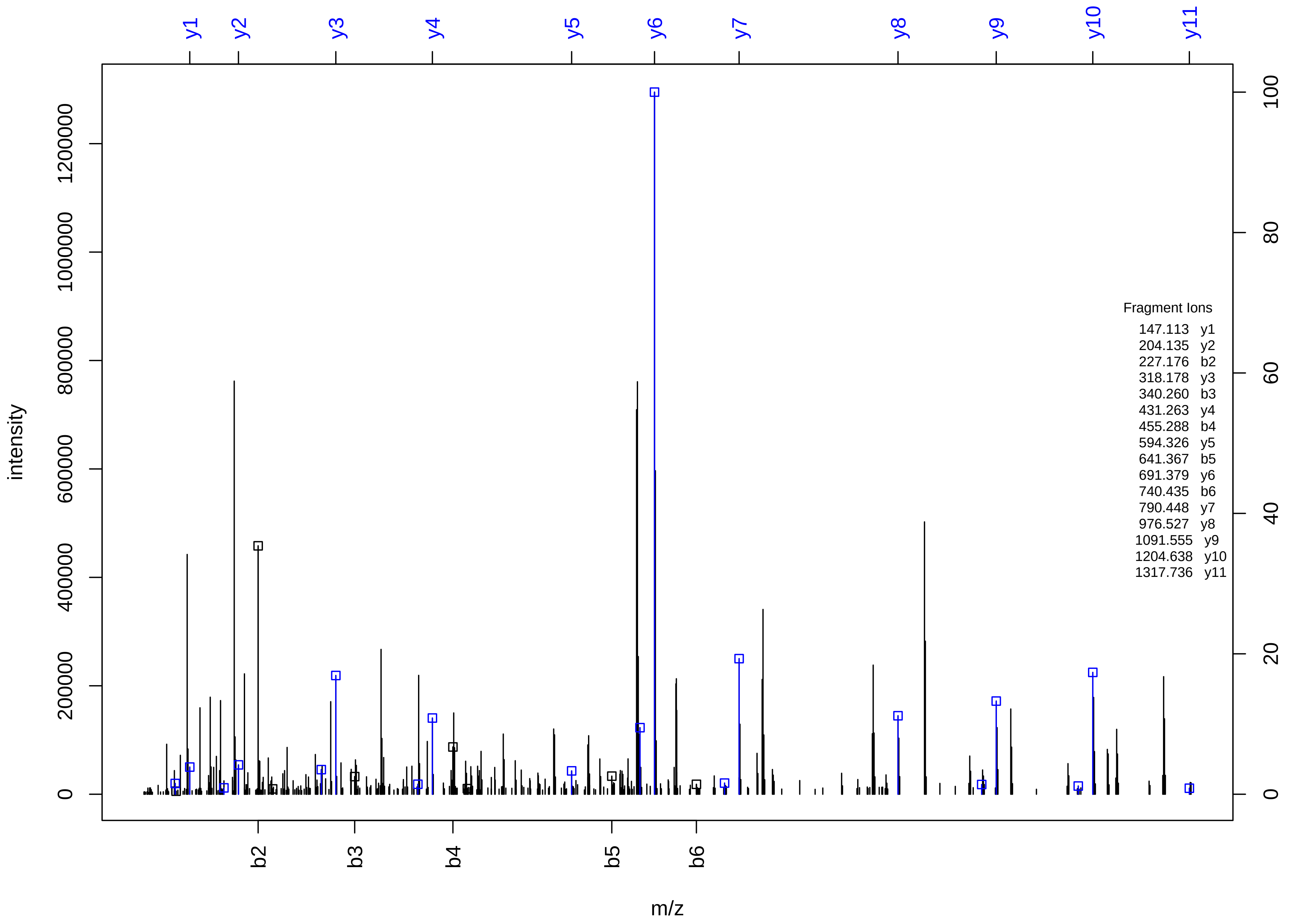

LILDWVPYINGK / File: sarah_20ip_20−P301L−3−1.raw; SpectrumID: ; scans: 52898

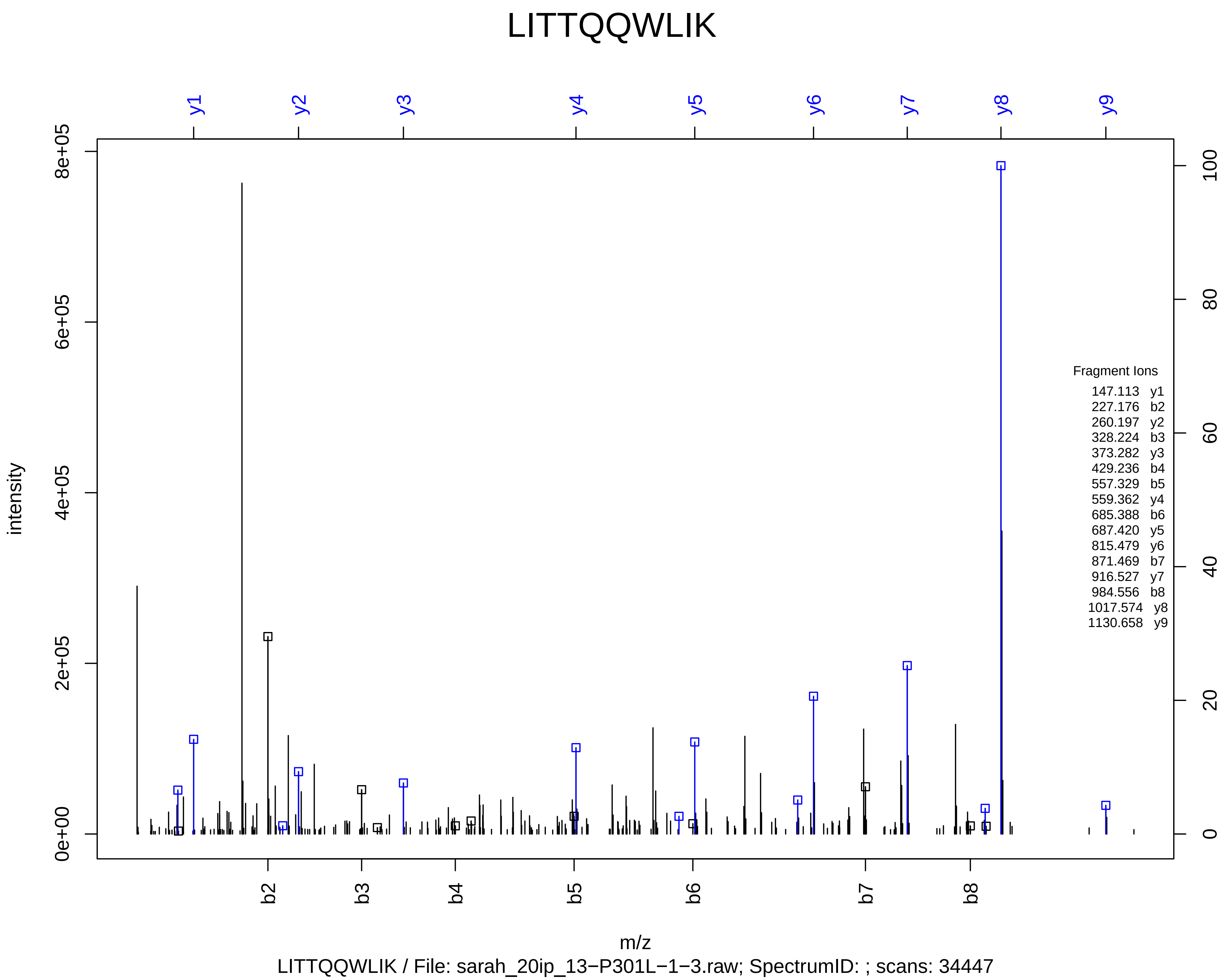
LITTQQWLIK
intensity
m/z
y1
y2
y3
y4
y5
y6
y7
y8
y9
b2
b3
b4
b5
b6
b7
b8
0e+00
2e+05
4e+05
6e+05
8e+05
0
20
40
60
80
100
Fragment Ions
147.113 y1
227.176 b2
260.197 y2
328.224 b3
373.282 y3
429.236 b4
557.329 b5
559.362 y4
685.388 b6
687.420 y5
815.479 y6
871.469 b7
916.527 y7
984.556 b8
1017.574 y8
1130.658 y9
LITTQQWLIK / File: sarah_20ip_13−P301L−1−3.raw; SpectrumID: ; scans: 34447

LLDSWFTSTQEK

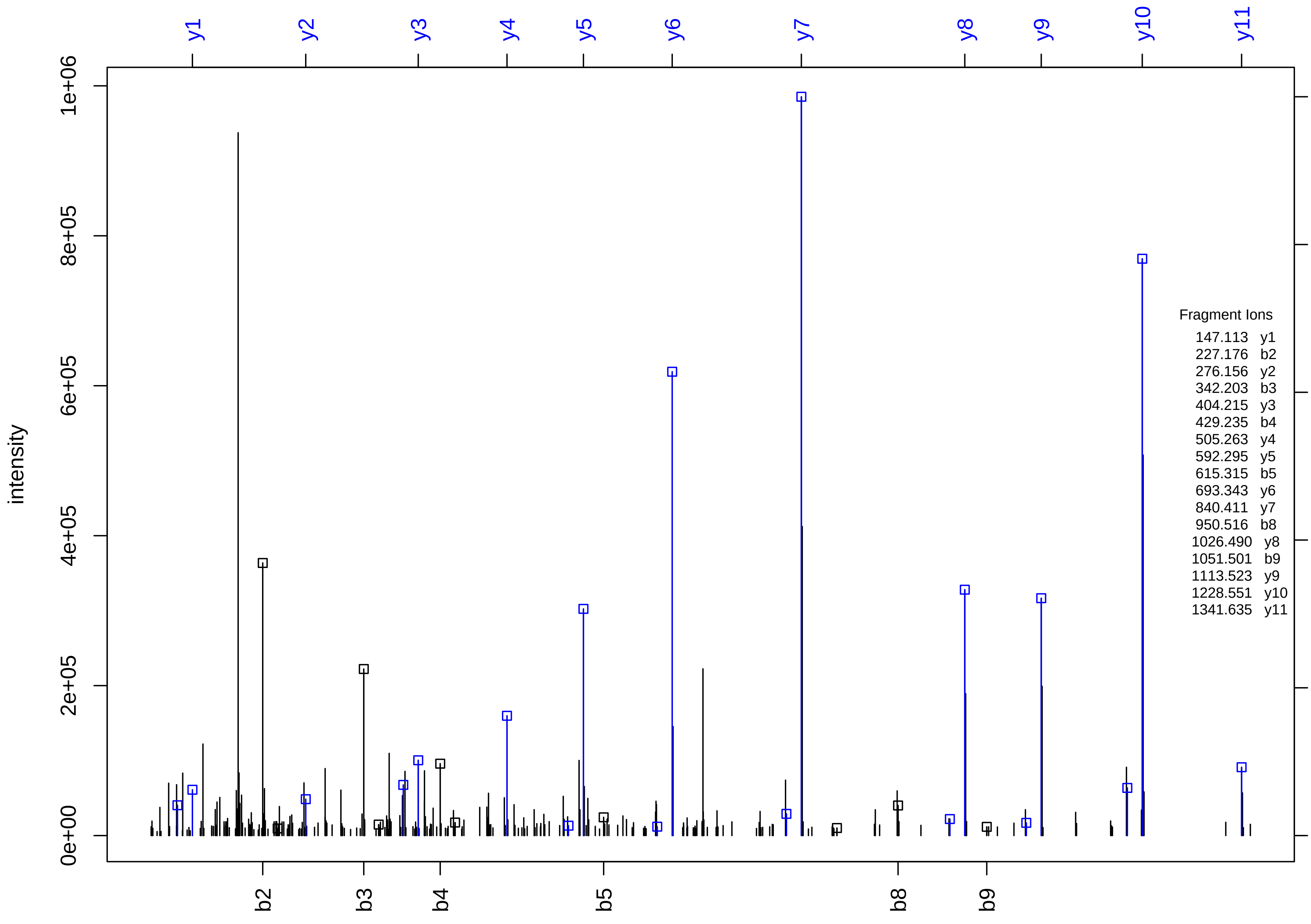

LLDSWFTSTQEK / File: sarah_20ip_12−P301L−1−2.raw; SpectrumID: ; scans: 38820

LLQIGNELR

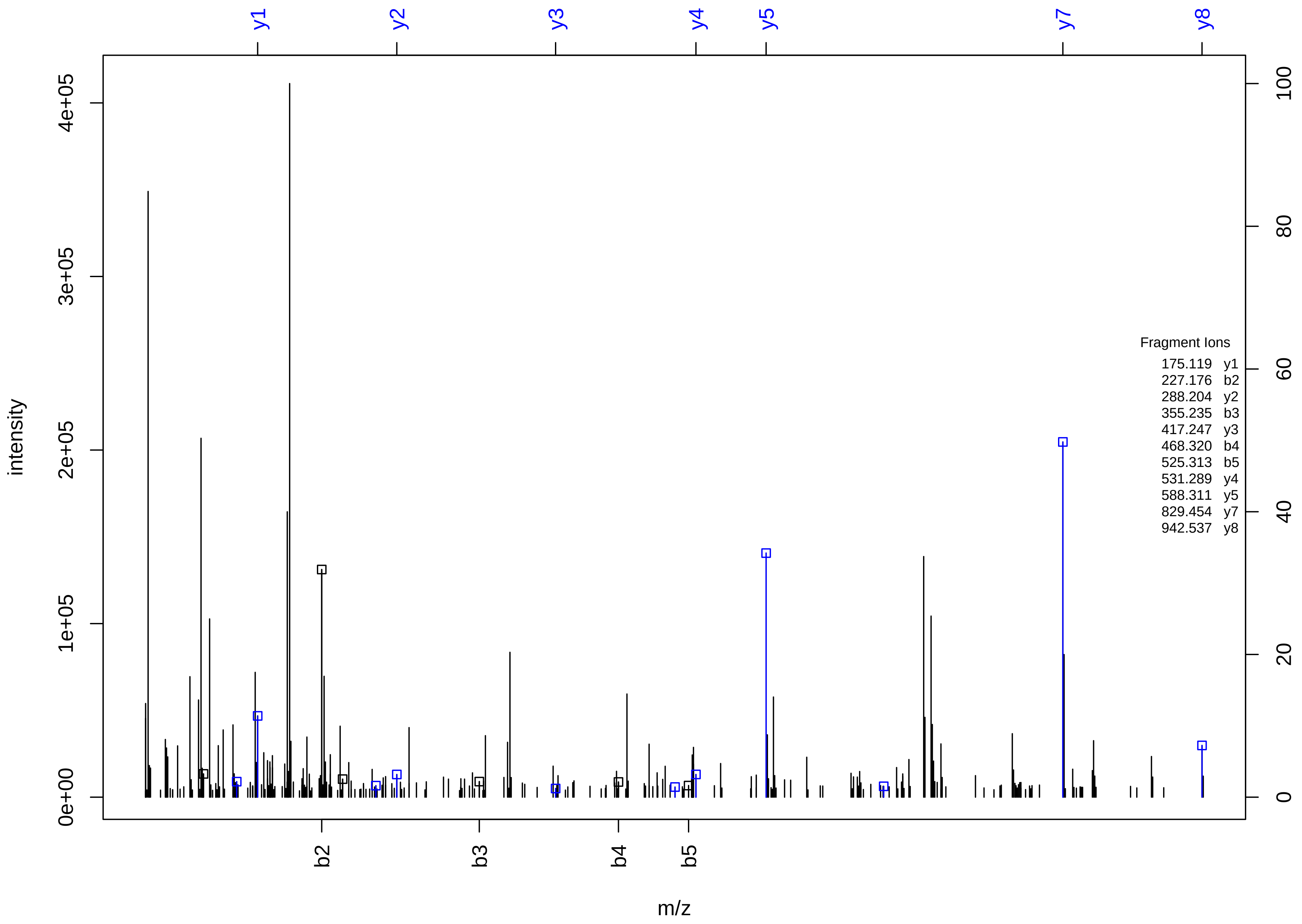

LLQIGNELR / File: sarah_20ip_19−P301L−3−2.raw; SpectrumID: ; scans: 21975

LLSVVPVPEGYSVK

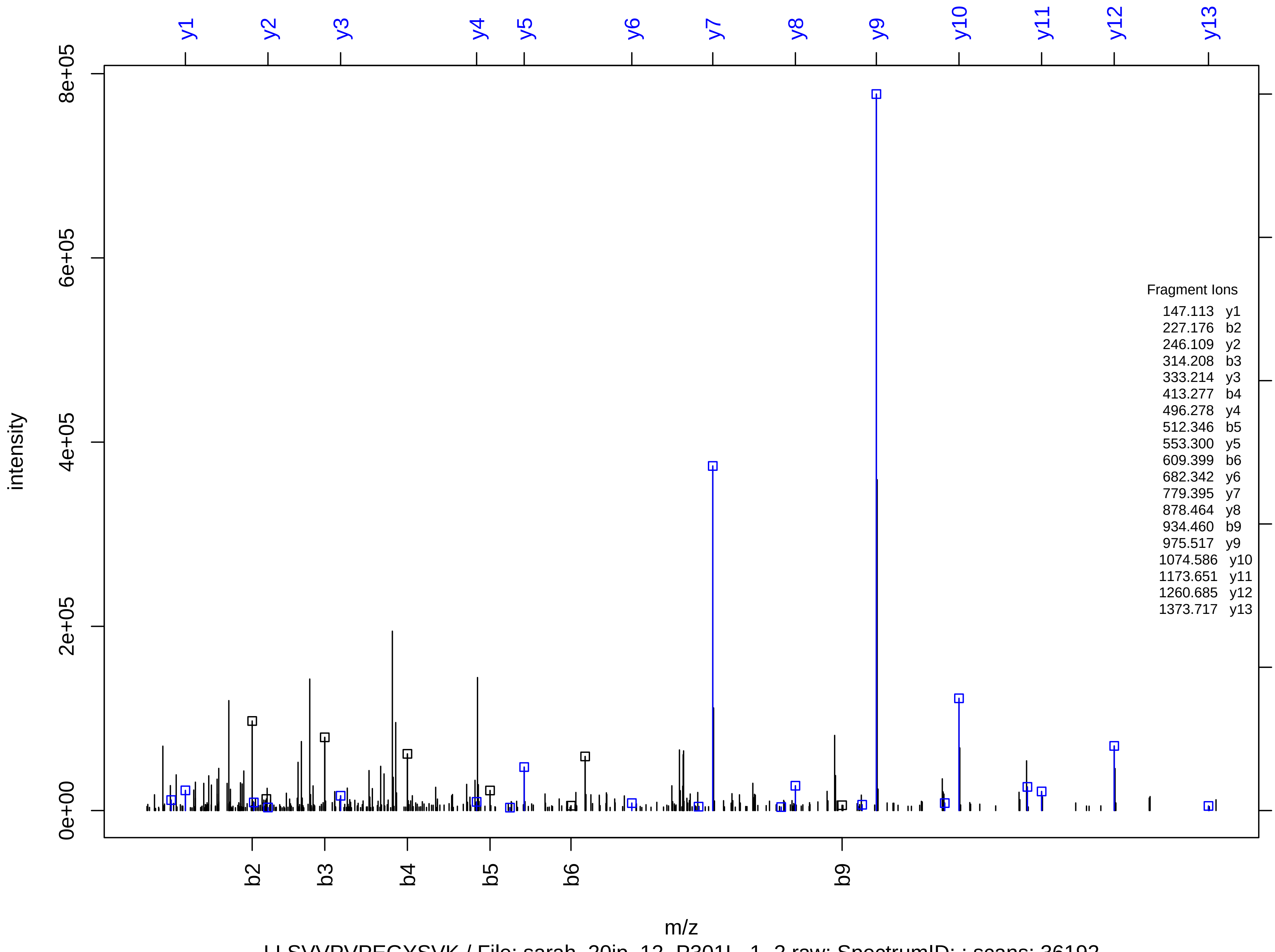

LLSVVPVPEGYSVK / File: sarah_20ip_12−P301L−1−2.raw; SpectrumID: ; scans: 36192

LNLGGDFIK

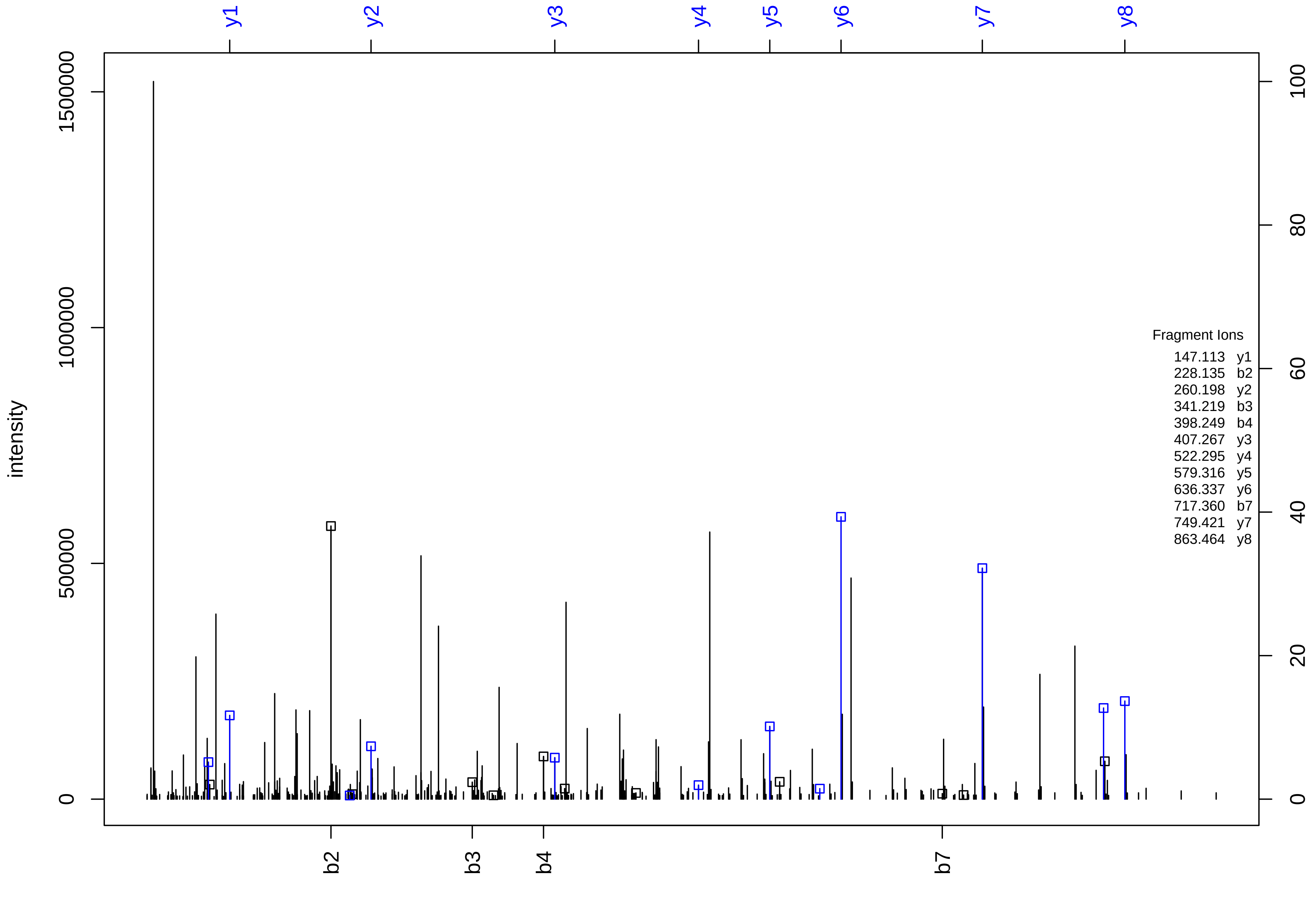

LNLGGDFIK / File: sarah_20ip_20−P301L−3−1.raw; SpectrumID: ; scans: 30706

NLPFSVENK

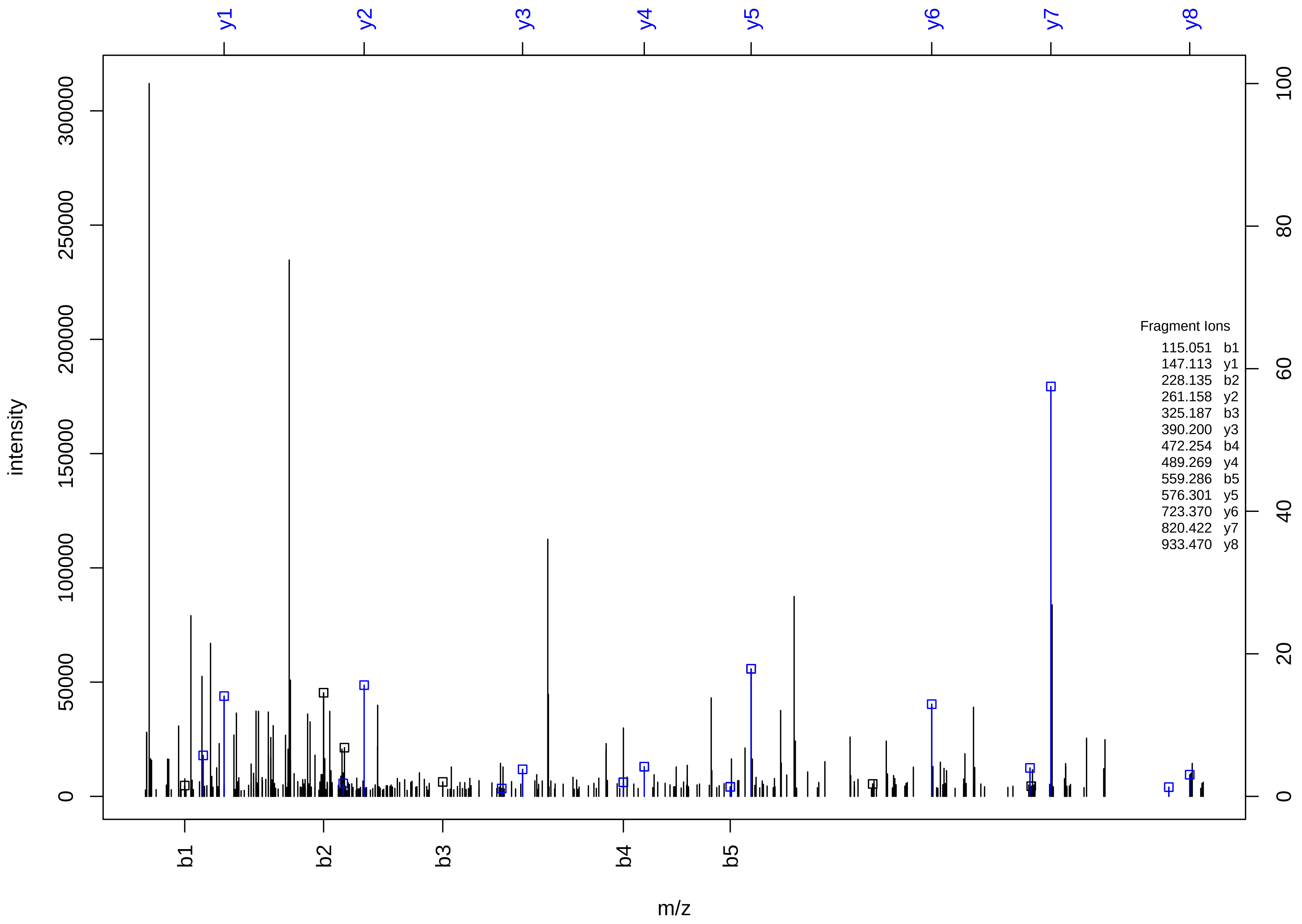

NLPFSVENK / File: sarah_20ip_19−P301L−3−2.raw; SpectrumID: ; scans: 20439

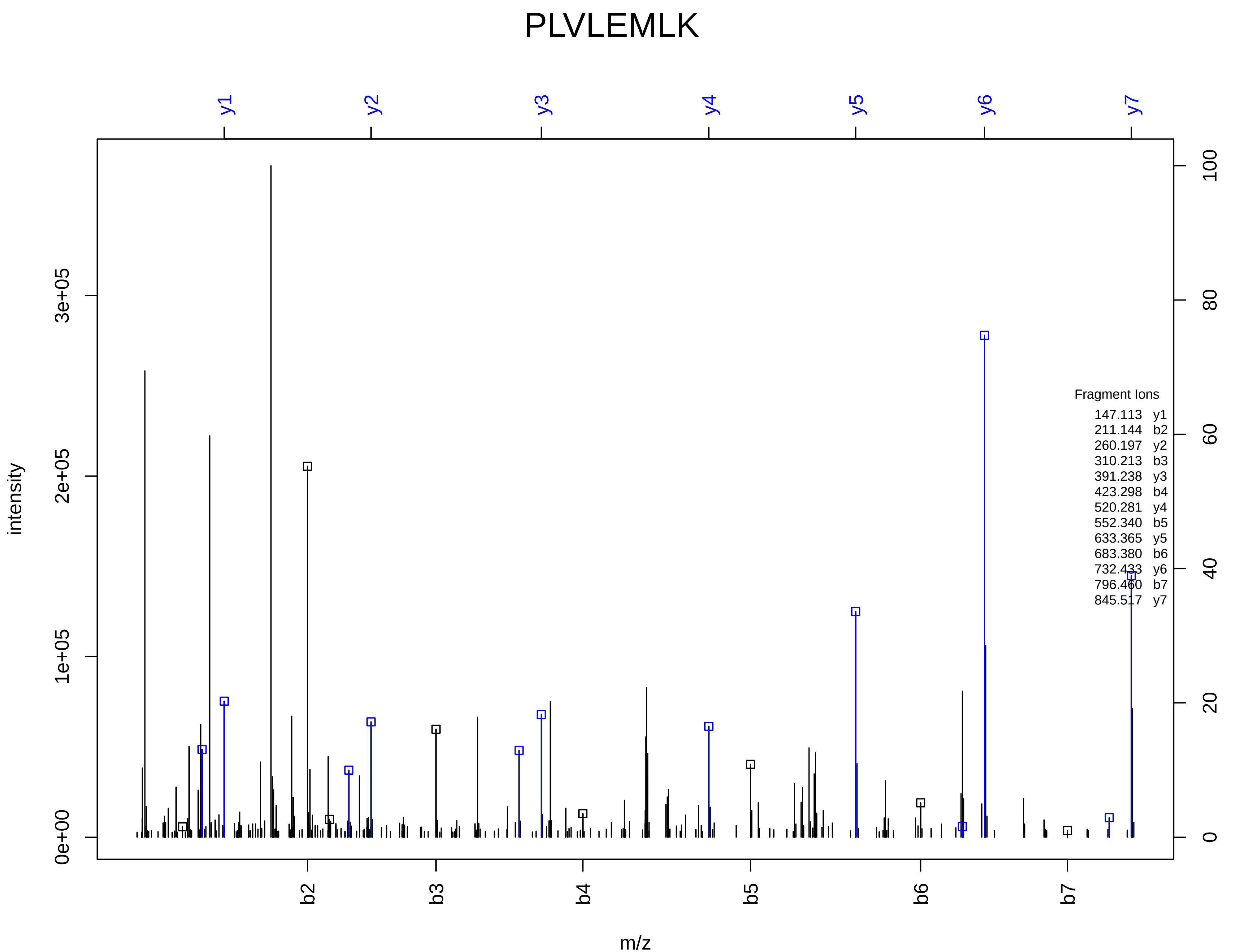

PLVLEMLK / File: sarah_20ip_12−P301L−1−2.raw; SpectrumID: ; scans: 39510

PLVLE*LK

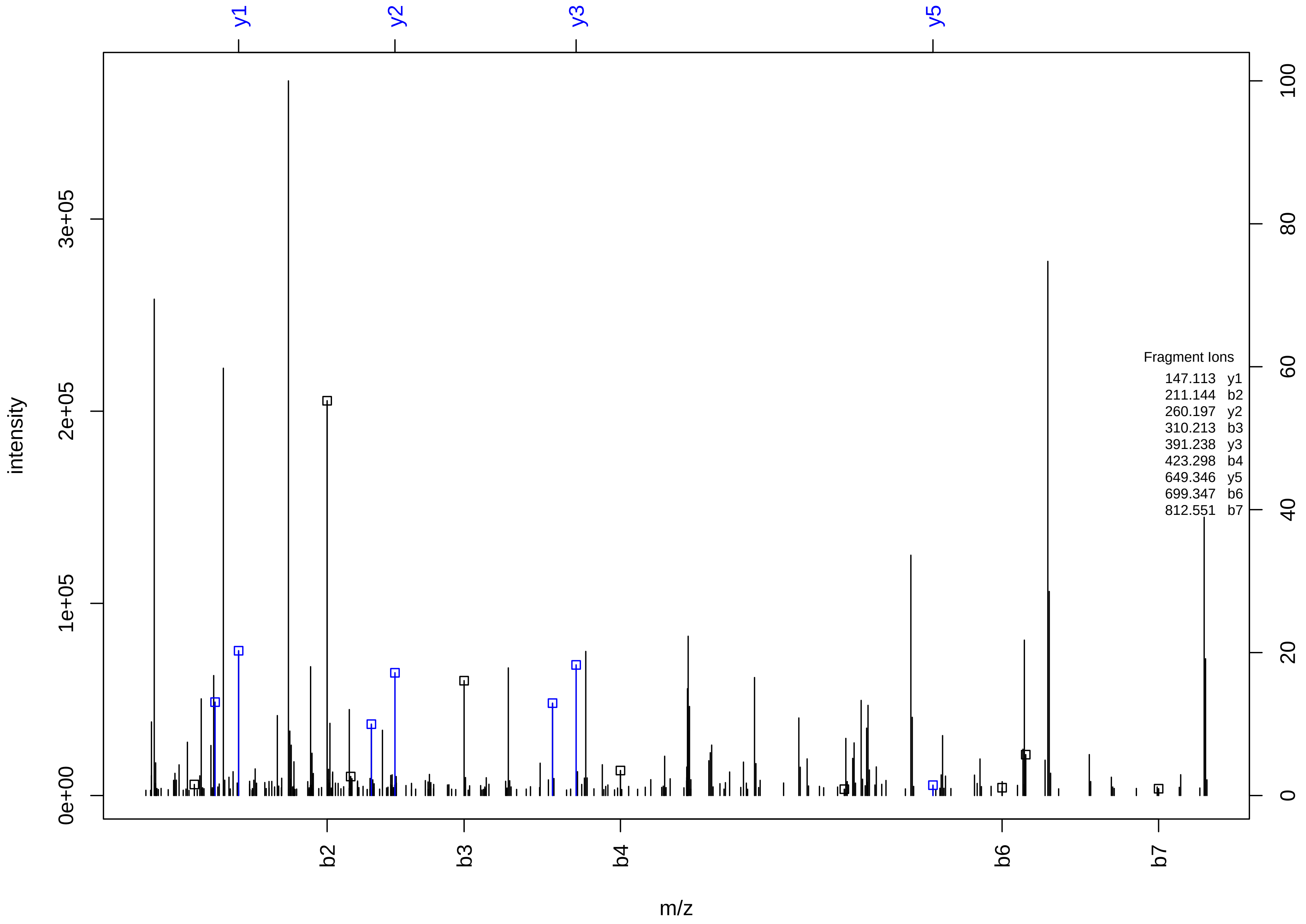

PLVLEMLK / File: sarah_20ip_12−P301L−1−2.raw; SpectrumID: ; scans: 39510

QNRPIPQWIR

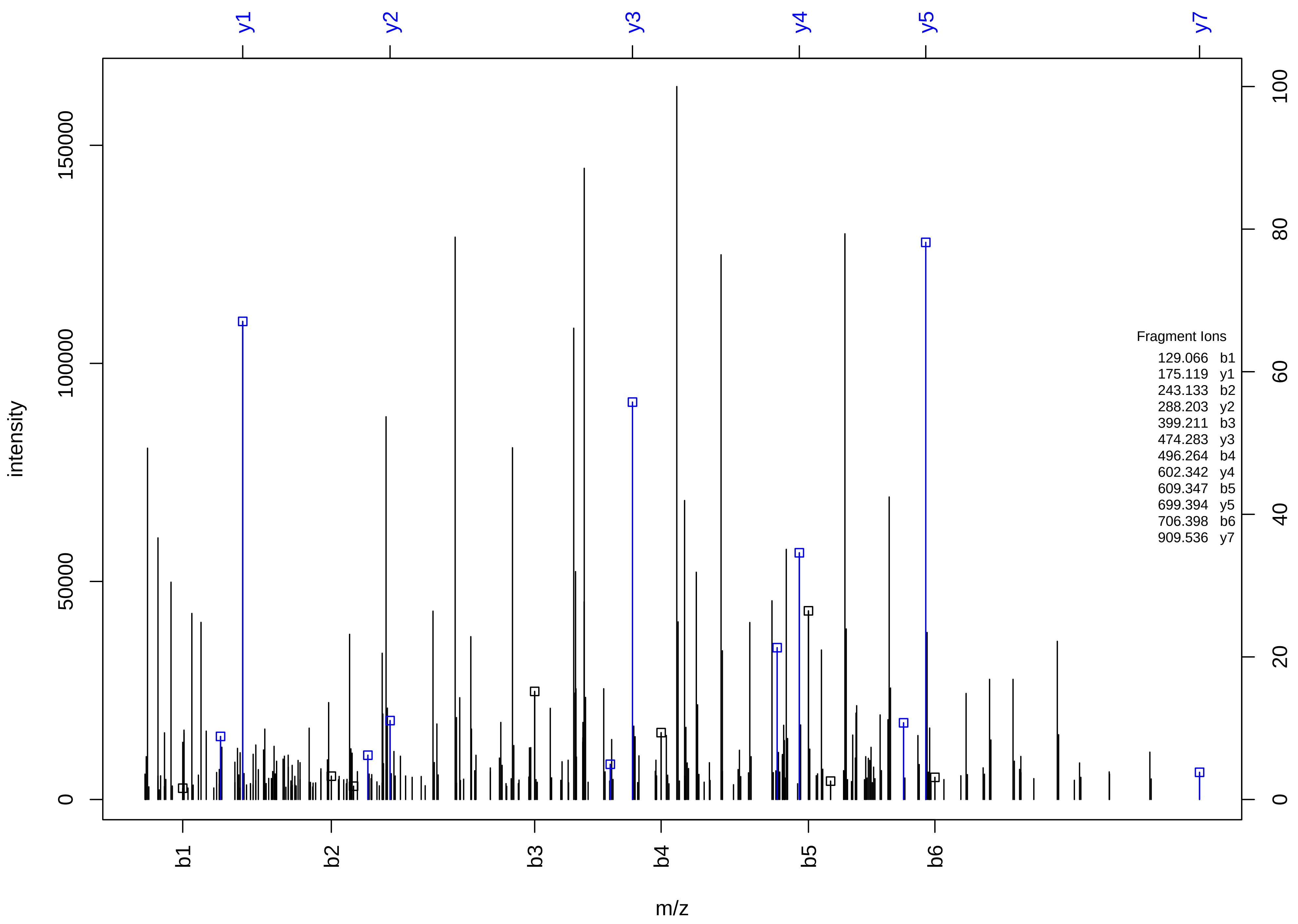

QNRPIPQWIR / File: sarah_20ip_15−P301L−2−1.raw; SpectrumID: ; scans: 18068

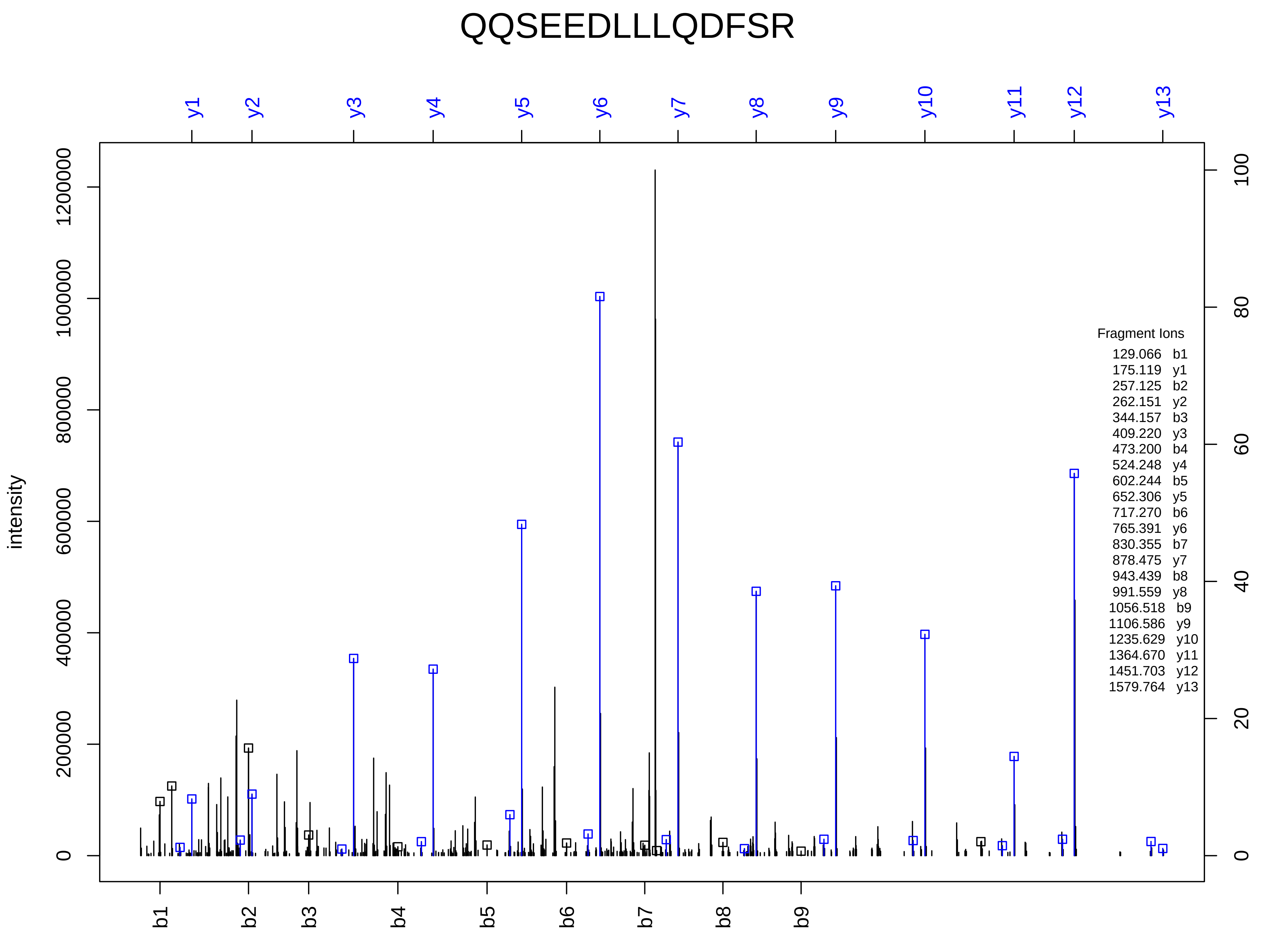

QQSEEDLLLQDFSR / File: sarah_20ip_12−P301L−1−2.raw; SpectrumID: ; scans: 42903

SDSYVELSQYR

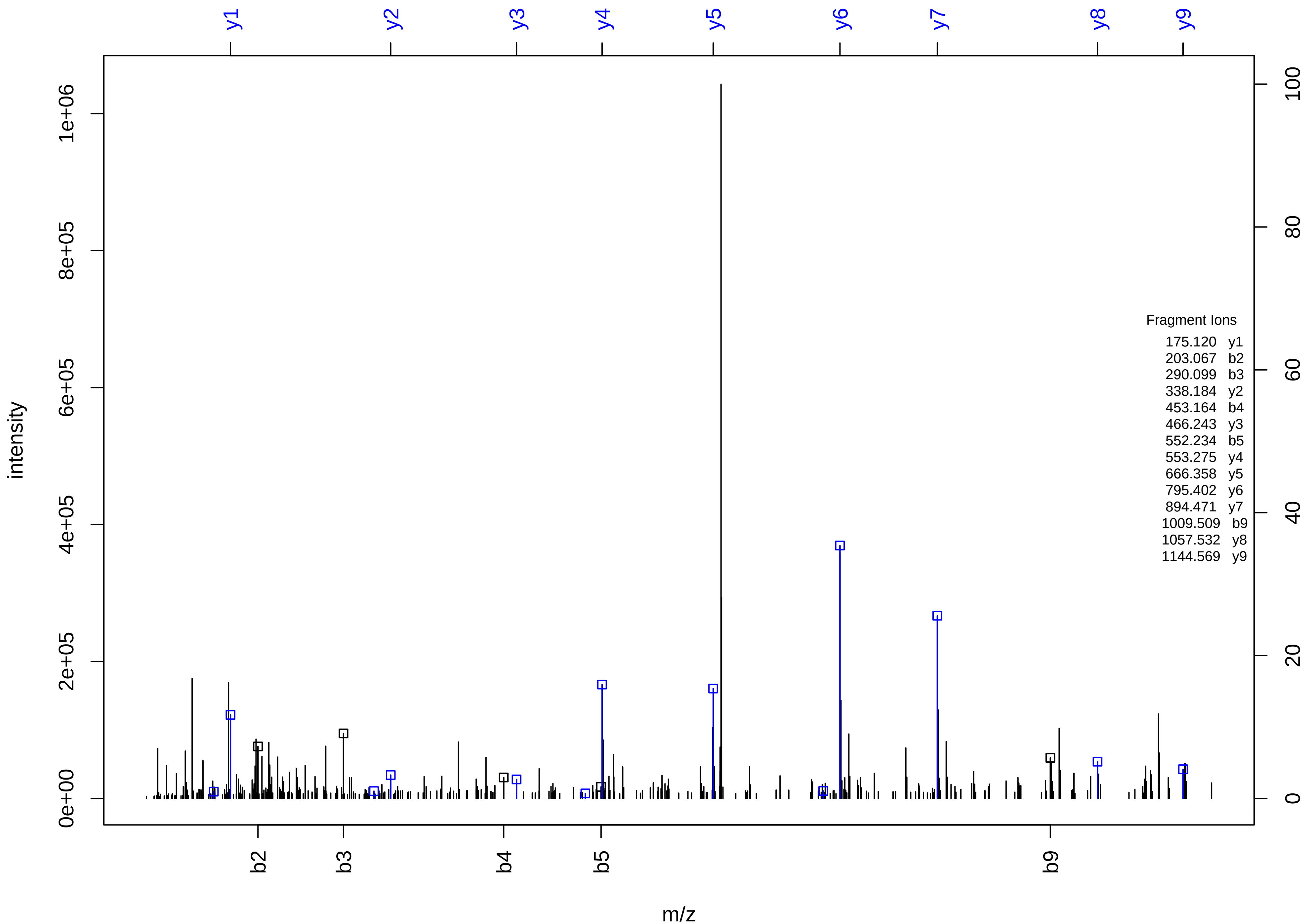

SDSYVELSQYR / File: sarah_20ip_20−P301L−3−1.raw; SpectrumID: ; scans: 21529

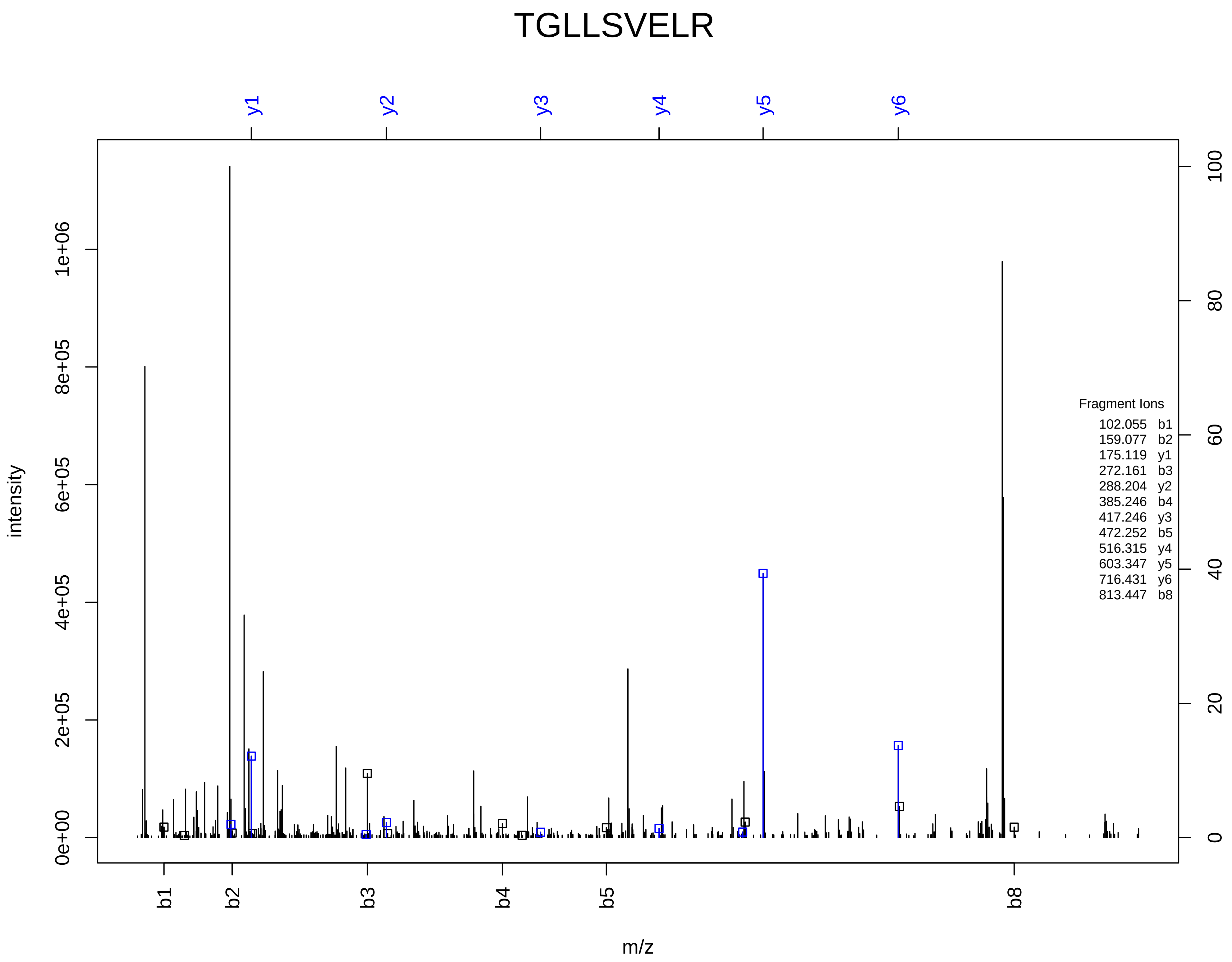
TGLLSVELR
y1
y2
y3
y4
y5
y6
intensity
0e+00
2e+05
4e+05
6e+05
8e+05
1e+06
0
20
40
60
80
100
Fragment Ions
102.055 b1
159.077 b2
175.119 y1
272.161 b3
288.204 y2
385.246 b4
417.246 y3
472.252 b5
516.315 y4
603.347 y5
716.431 y6
813.447 b8
b1
b2
b3
b4
b5
b8
m/z
TGLLSVELR / File: sarah_20ip_12−P301L−1−2.raw; SpectrumID: ; scans: 25182

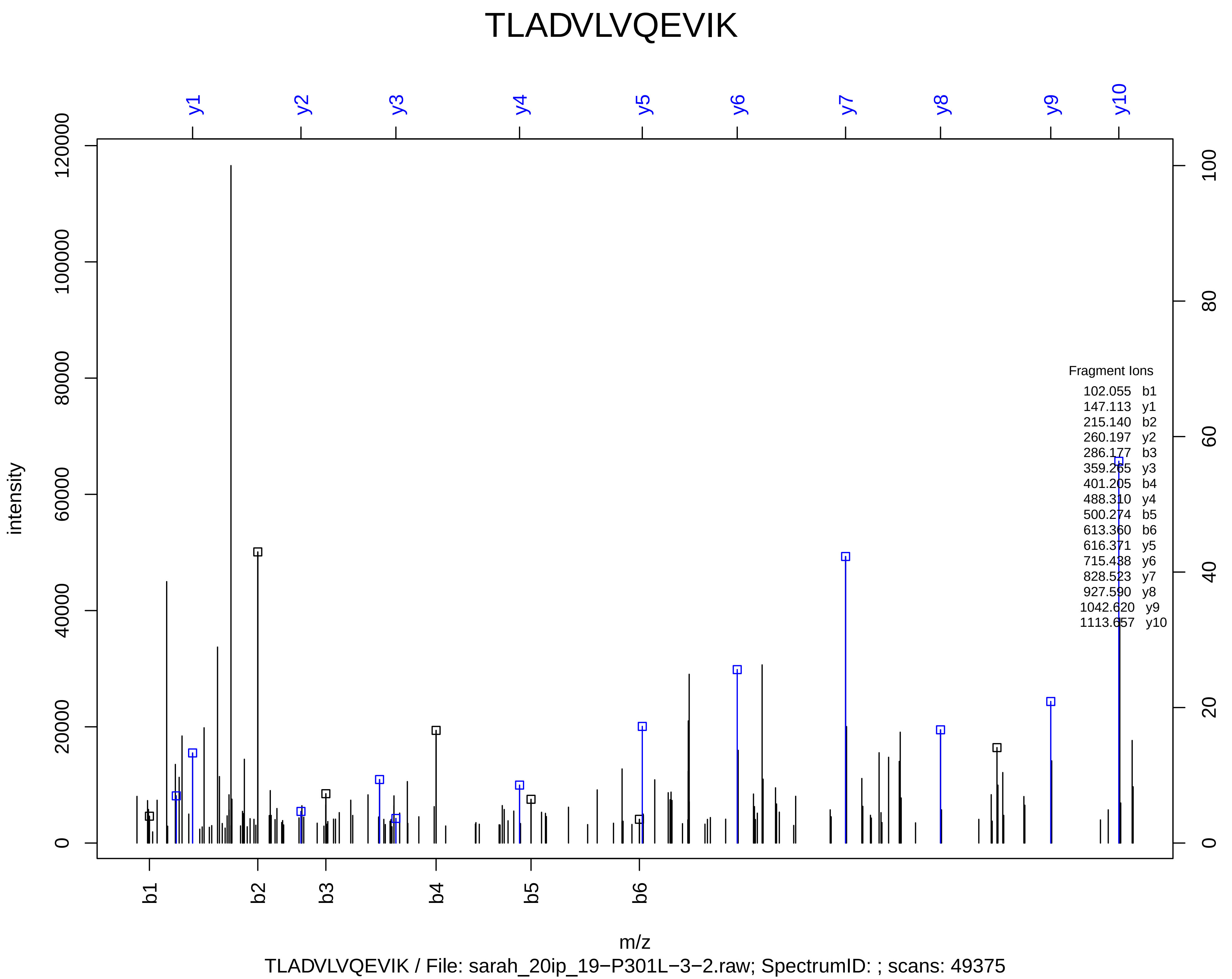
TLADVLVQEVIK
intensity
m/z
y1 y2 y3 y4 y5 y6 y7 y8 y9 y10
b1 b2 b3 b4 b5 b6
Fragment Ions
102.055 b1
147.113 y1
215.140 b2
260.197 y2
286.177 b3
359.265 y3
401.205 b4
488.310 y4
500.274 b5
613.360 b6
616.371 y5
715.438 y6
828.523 y7
927.590 y8
1042.620 y9
1113.657 y10
TLADVLVQEVIK / File: sarah_20ip_19−P301L−3−2.raw; SpectrumID: ; scans: 49375

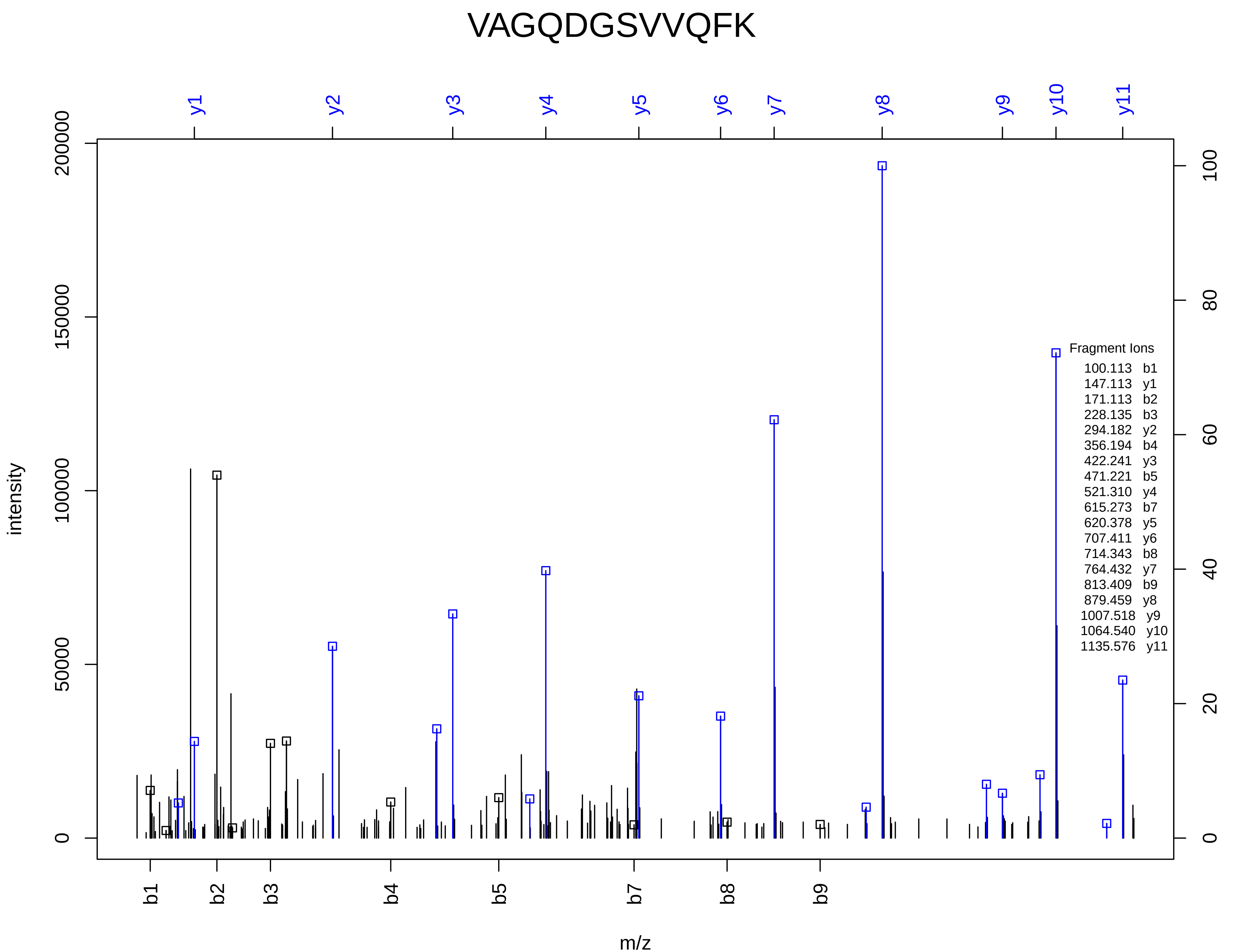
VAGQDGSVVQFK
intensity
m/z
y1
y2
y3
y4
y5
y6
y7
y8
y9
y10
y11
b1
b2
b3
b4
b5
b7
b8
b9
Fragment Ions
100.113 b1
147.113 y1
171.113 b2
228.135 b3
294.182 y2
356.194 b4
422.241 y3
471.221 b5
521.310 y4
615.273 b7
620.378 y5
707.411 y6
714.343 b8
764.432 y7
813.409 b9
879.459 y8
1007.518 y9
1064.540 y10
1135.576 y11
VAGQDGSVVQFK / File: sarah_20ip_11−P301L−1−1.raw; SpectrumID: ; scans: 13358

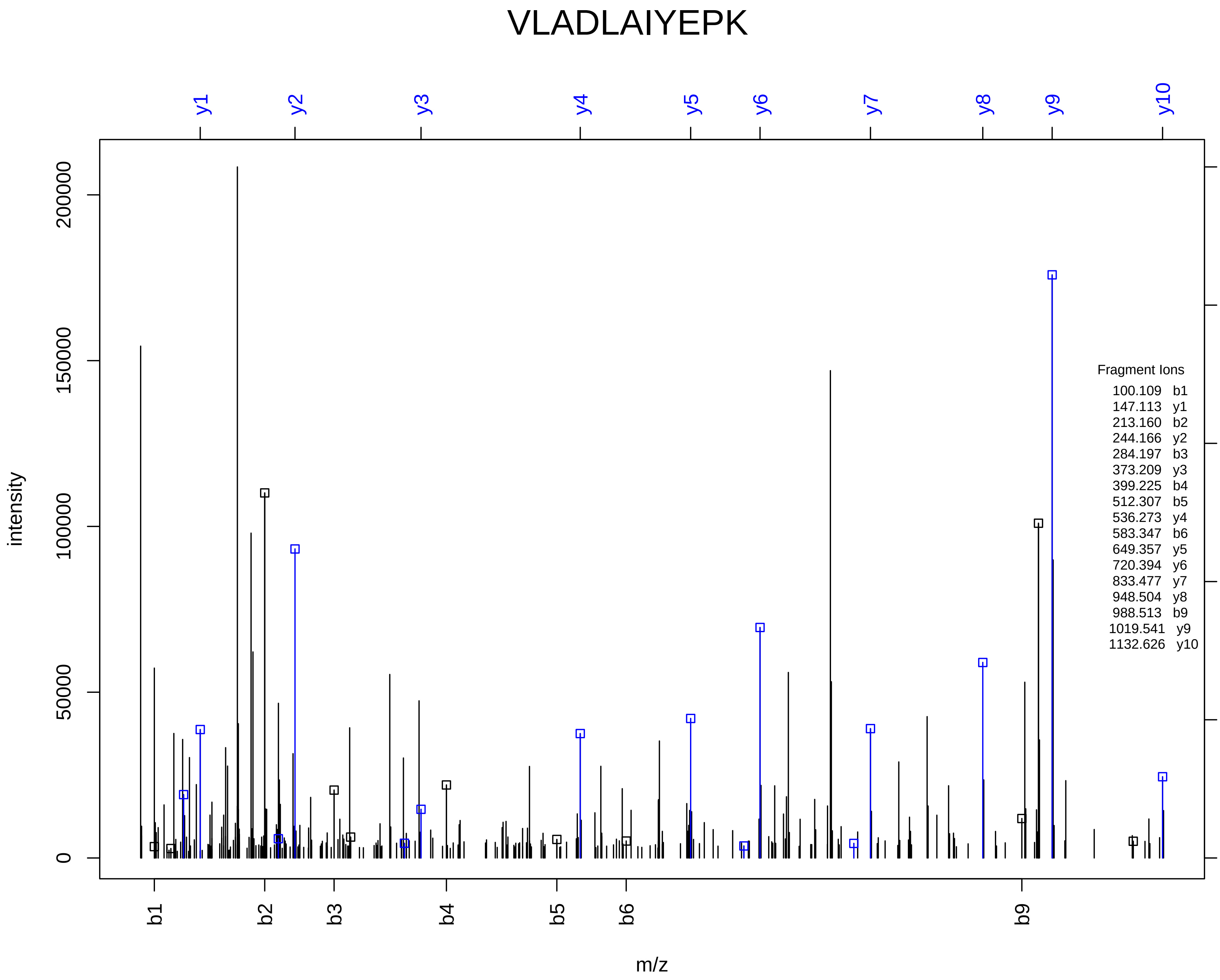

VLADLAIYEPK / File: sarah_20ip_12−P301L−1−2.raw; SpectrumID: ; scans: 34246

VPFLVLEC[57.0215]PNLK

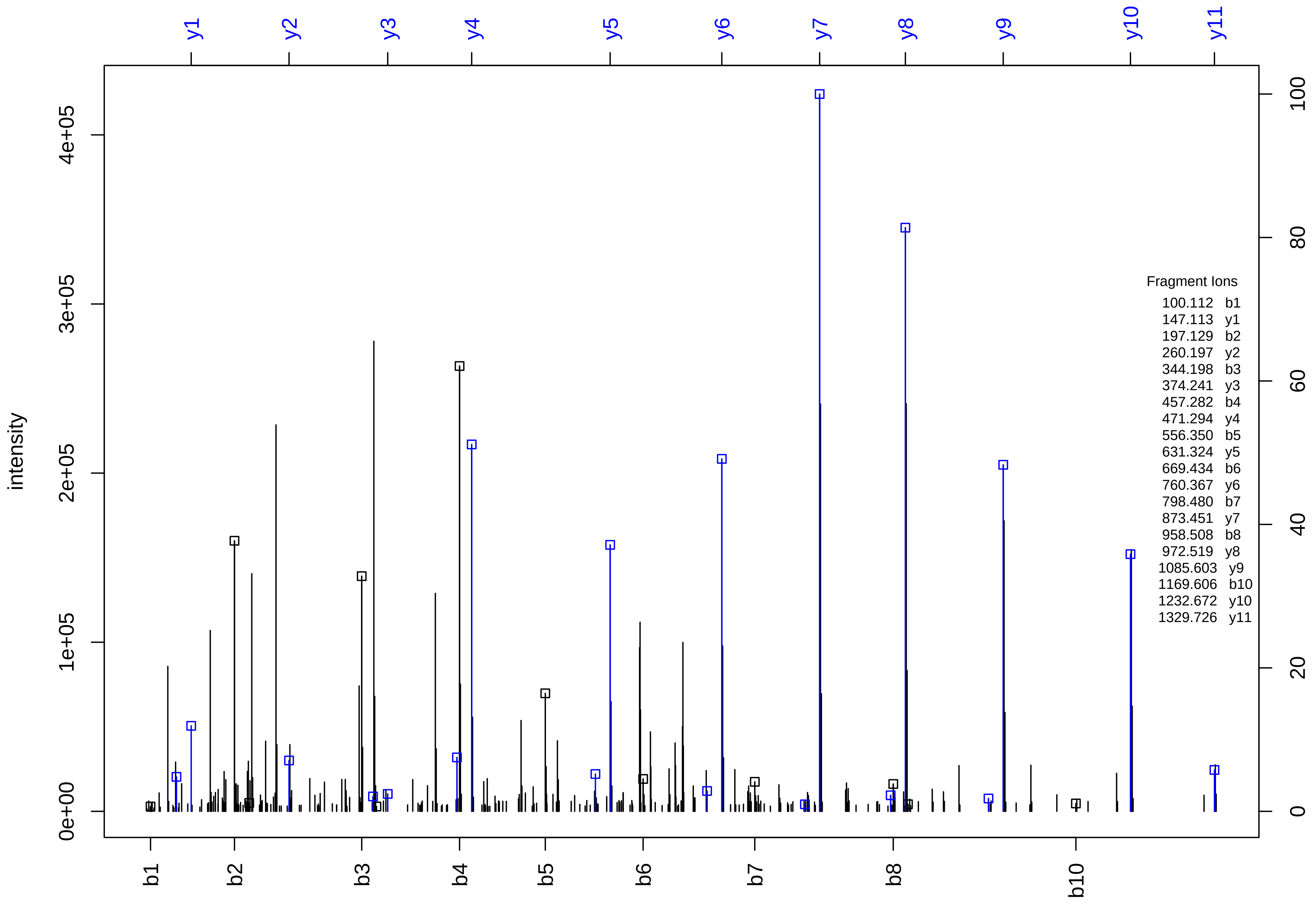

VPFLVLECPNLK / File: sarah_20ip_13−P301L−1−3.raw; SpectrumID: ; scans: 43101

VTPPAVTGSPEFER

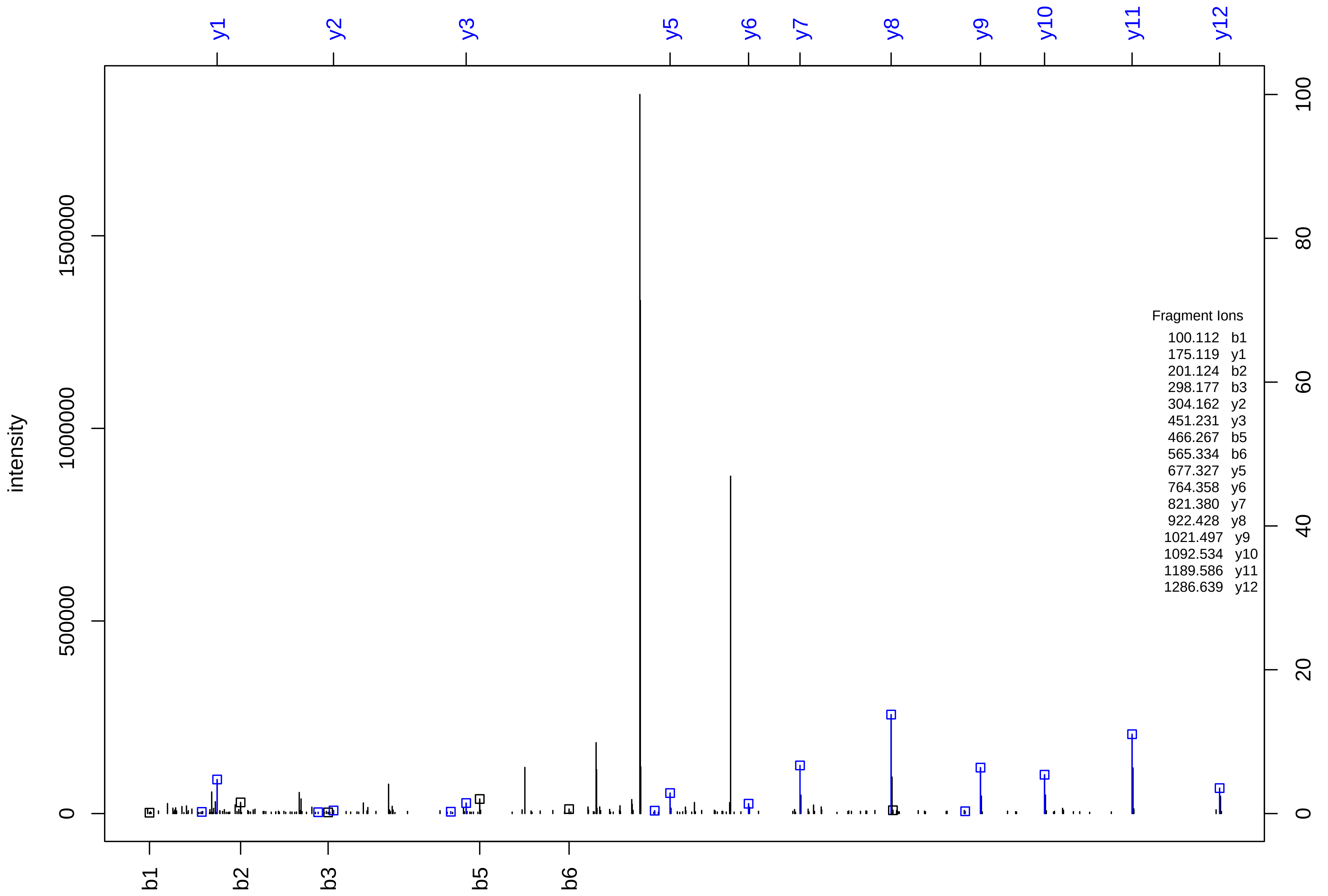

VTPPAVTGSPEFER / File: sarah_20ip_12−P301L−1−2.raw; SpectrumID: ; scans: 19433
