# Supplementary material for: Proximity Labeling of the Tau Repeat Domain Enriches RNA-Binding Proteins That Are Altered in Alzheimer's Disease and Related Tauopathies
Source: Mol Cell Proteomics. 2025 Nov 7;25(1):101458. doi: 10.1016/j.mcpro.2025.101458 (PMC12796112; doi:10.1016/j.mcpro.2025.101458)
Supplement: Methods S1 [file mmc14.pdf]

Supplemental materials & methods

Blue Native Poly-acrylamide Gel Electrophoresis

HEK cells were grown in 10-well plates, transfected, and biotin labeled according to standard procedures. Cell were scraped into lo-bind Eppendorf tubes, centrifuged for 5 min at 500 x *g* at 4°C, the washed in ice-cold 1X PBS containing 1X HALT protease inhibitor. Centrifugation and PBS wash was repeated, then PBS was aspirated before cells were flash frozen in -80°C for future use. Cell pellets were thawed on wet ice, then lysed to maintain native conditions (20 mM Tris-HCl pH 7.5, 50 mM NaCl, 1 mM PMSF, 0.1% Triton-X, and 1X HALT protease and phosphatase inhibitor). Lysates were incubated on ice for 20 minutes then debris was cleared by centrifugation at 18,213 x *g* for 30 min at 4°C. The resulting protein supernatant was collected, and total concentration was determined by BCA assay (Pierce). Sample preparation was conducted following established protocols[23, 115]. Briefly, 20 µg each lysate was incubated in NativePAGE loading buffer (5% glycerol, 50 mM TCEP, 0.02% Coomassie G-250, 1X NativePAGE Sample Buffer) then incubated on ice for 10 minutes. Recombinant K18 and full-length tau 441 were prepared in roughly equimolar concentrations of 0.2 µg K18 monomer and 0.9 µg full-length tau 441. Recombinant proteins were prepared in the same NativePAGE loading buffer and allowed to incubate at room temperature for 30 minutes. 10 µL NativeMark protein ladder (Invitrogen, #LC0725) was loaded per gel.

Samples were loaded onto a 4-16% NativePAGE Bis-Tris Gel (Invitrogen, #BN1004) using the XCell SureLock Mini-Cell Electrophoresis System. Proteins were resolved by electrophoresis at 150 V with 1X Native PAGE running buffer (Invitrogen, #BN2007) in the outer chamber dark blue cathode buffer in the inner chamber. After 15 minutes, dark blue cathode buffer was aspirated and light blue cathode buffer replaced in the inner chamber until the dye front reached the end of the gel. The gel was then incubated at room temperature in 50 mM Tris-HCl with 1% (v/v) SDS for 30 min to release proteins prior to protein transfer. Proteins were transferred to PVDF membrane at 20V for 7 min using the iBolt semi-dry transfer system. The membrane was then incubated in 8% acetic acid for 5 min to fix proteins to the membrane, rinsed with water, then allowed to air dry. The PVDF membrane was rinsed with methanol to remove excess Coomassie dye, then proceeded with standard WB procedure to detect tau-MTBR.

Thioflavin T *in vitro* aggregation assay

Recombinant K18 tau monomer (25 µM) (SPR-328B) alone, HEK293 FRET TauRD, HEK293 sTurbo TauRD P301L lysates each at 5 µg/mL with and without K18 tau monomer (5 µg/mL) were incubated in filtered PBS (pH 7.4), and 20 µM ThT. The final volume within each well was 50 µL. The assay was conducted in quadruplicates using chilled 384 well black clear bottom plates. Fluorescence was captured at 420 Ex, 480 Em for 96 hours at 15 min intervals at 37ºC using Synergy H1 (Biotek) microplate reader. Average ThT signal across 8 replicates (20 µM) was measured and subtracted from individual experimental technical replicates to remove background fluorescence. The pre-aggregation nucleation phase (t= 24 HR) and final plateau phase relative (t= 96 hr) fluorescence intensities were plotted using GraphPad Prism (v.10).

Analyses for Supplemental Figures

RNA-binding protein (RBP) module analyses and visualization: To annotate RBP types associated with RBP-enriched modules, TauRD interactome was merged with previously published data describing 356 annotated RBPs for respective function, localization, and amino acid motifs[84] and visualized via ggplot().

KEGG pathway analysis and visualization: Interactome proteins mapping to insoluble RBP modules (M18 & M5) and total list of enriched proteins in sTurbo TauRD, hTissue, or insolAD were converted to Entrez IDs using the bitr function from the clusterProfiler[138] package and org.Hs.eg.db package. KEGG pathway[139] enrichment analysis was performed for each comparison list using the enrichKEGG function from clusterProfiler. The analysis was conducted with the following parameters: organism = "hs", keyType = "ncbi-geneid", pvalueCutoff = 0.05, qvalueCutoff = 0.2, and pAdjustMethod = "BH". For each comparison, the top 5 enriched KEGG pathways based on adjusted pvalue were selected for vIsualization. Pathways diagrams were rendered with the pathview package[140]. For selected pathways, hsa03015 mRNA surveillance pathway[141-145] and hsa03040 Spliceosome[146-149], a presence-based plot was generated by mapping the identified Entrez IDs of each comparison (sTurbo TauRD, hTissue, and insolAD) to the pathway diagram. All analyses were performed in R (v 23.2.0).
